# In vivo CRISPR screen reveals regulation of macrophage states in neuroinflammation

**DOI:** 10.1038/s41593-025-02151-6

**Published:** 2025-12-04

**Authors:** Clara de la Rosa, Arek Kendirli, Seren Baygün, Franz Bauernschmitt, Anna S. Thomann, Ilgin Kisioglu, Daniela Beckmann, Yves Carpentier Solorio, Veronika Pfaffenstaller, Yi-Heng Tai, Niel Mehraein, Paula Sanchez, Lena Spieth, Lisa Ann Gerdes, Eduardo Beltran, Klaus Dornmair, Mikael Simons, Anneli Peters, Marc Schmidt-Supprian, Martin Kerschensteiner

**Affiliations:** 1 Institute of Clinical Neuroimmunology, University Hospital, Ludwig-Maximilians-Universität Munich, Munich, Germany; 2 Biomedical Center (BMC), Medical Faculty, Ludwig-Maximilians-Universität Munich, Martinsried, Germany; 3 Graduate School of Systemic Neurosciences, Ludwig-Maximilians-Universität Munich, Munich, Germany; 4 Munich Cluster of Systems Neurology (SyNergy), Munich, Germany; 5 Institute of Experimental Haematology, Center for Translational Cancer Research (TranslaTUM), School of Medicine and Health, Technical University Munich, Munich, Germany; 6 Institute of Neuronal Cell Biology, Technical University Munich, Munich, Germany; 7 German Center for Neurodegenerative Diseases (DZNE), Munich, Germany; 8 Institute of Stroke and Dementia Research, University Hospital, Ludwig-Maximilians-Universität Munich, Munich, Germany

**Keywords:** Neuroimmunology, Monocytes and macrophages, Autoimmunity, Multiple sclerosis

## Abstract

Here we established an in vivo CRISPR screening pipeline using genetically editable progenitor cells to dissect macrophage regulation in mouse models of multiple sclerosis (MS). Screening over 100 cytokine receptors and signaling molecules identified interferon-γ, tumor necrosis factor, granulocyte-macrophage colony-stimulating factor and transforming growth factor-β as essential regulators of macrophage polarization in vivo. Single-cell transcriptomics confirmed that transferred progenitor cells generate all blood-derived CNS myeloid cell populations, enabling Perturb-seq analysis of cytokine actions in neuroinflammation. Combined with biosensor expression, our approach allows monitoring cytokine effects on myeloid cell migration, debris phagocytosis and oxidative activity in vivo. Comparative transcriptomic analyses revealed conserved neuroinflammatory cytokine signatures across myeloid populations, CNS compartments and species, elucidating cytokine cues shaping myeloid function in the cerebrospinal fluid and parenchyma of individuals with MS. This versatile pipeline thus provides a scalable framework for high-resolution analysis of macrophage states and uncovers the cytokine signals that underlie their regulation in MS and MS models.

## Abstract (web summary)

De la Rosa et al. developed an in vivo CRISPR screening system to dissect macrophage regulation in multiple sclerosis models, revealing key cytokine signaling pathways that control myeloid cell behavior in neuroinflammation.

## Main

MS is an autoimmune disorder characterized by the infiltration of adaptive and innate immune cells into the CNS, resulting in demyelination and axonal loss^1^. Among the infiltrating immune cells, macrophages are the most abundant^2^, and their numbers correlate with the extent of tissue damage^3,4^. In experimental models, monocyte-derived macrophages are critical for disease manifestation and progression^5–7^ as they secrete pro-inflammatory mediators, proteolytic enzymes, and reactive oxygen and nitrogen species that exacerbate tissue damage^8,9^. Conversely, macrophages also contribute to repair by clearing myelin debris, remodeling tissue and releasing trophic factors^10,11^. These divergent roles illustrate the remarkable plasticity of macrophages, which adopt context-dependent polarization states shaped by their microenvironment and cellular condition^12–14^. In MS models, macrophages transition from an inducible nitric oxide synthase (iNOS)-positive, lesion-promoting phenotype to a lesion-resolving state characterized by arginase-1 (Arg1) expression^8,15^. However, the molecular cues that instruct these transitions in vivo remain incompletely defined, particularly within the inflamed CNS.

Recently, pooled CRISPR screens have shown to be capable of identifying the essential signaling components that underlie complex cellular phenotypes^16,17^. In vitro, such pooled CRISPR screens have helped to comprehensively dissect important macrophage functions including regulation of their survival and phagocytic capabilities^18,19^; yet, these techniques have not enabled CRISPR screening of macrophages in an inflamed tissue environment. Here, we established a rapid, versatile and scalable in vivo CRISPR screening approach that allows functional analysis of more than 100 genes in myeloid cells in a single experiment. We used this approach to deconstruct the regulation of macrophage states in MS models and identified four essential cytokines that instruct macrophage polarization in neuroinflammation.

## Results

### Hoxb8FL-derived myeloid cells enter neuroinflammatory lesions and polarize comparably to endogenous monocyte-derived macrophages

**Fig. 1:**
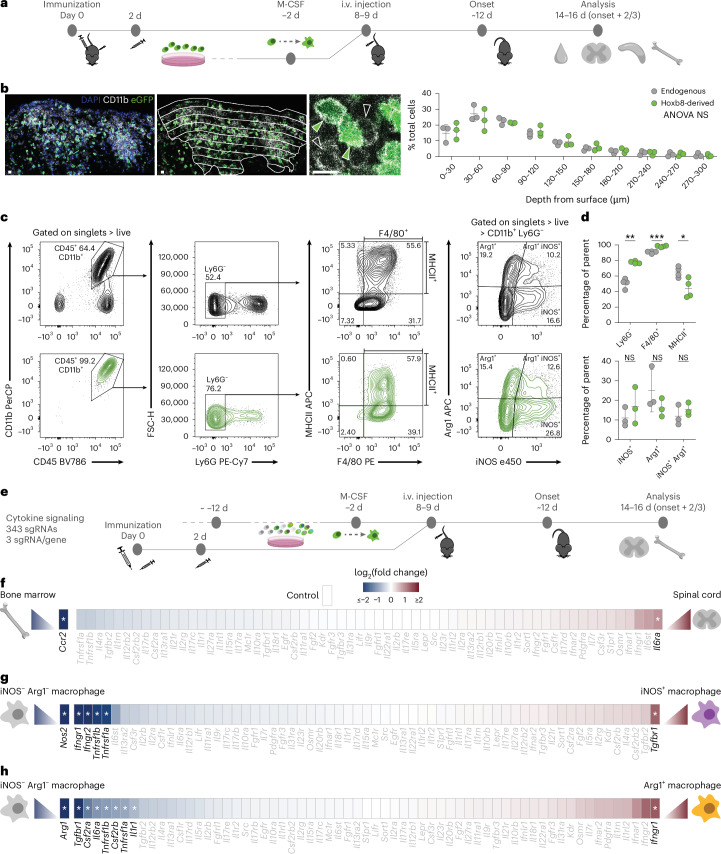
In vivo CRISPR screen identifies cytokine receptors regulating macrophage polarization in neuroinflammatory lesions. **a**, Scheme of the new experimental approach. Hematopoietic precursor (Hoxb8FL) cells were pre-differentiated in vitro for 48 h toward the myeloid lineage then i.v. injected into EAE-induced mice before disease onset. **b**, Histological characterization of the distribution of Hoxb8FL-derived and endogenous myeloid cells in EAE lesions in the spinal cord. Right, relative abundance of cells through lesions (depth from the spinal cord surface). *N* = 3 animals, 8, 11 and 11 lesions per animal. Scale bars, 10 μm. **c**, Representative flow cytometry plots of the transferred Hoxb8FL-derived cells (bottom row, green) compared to endogenous cells (top row, gray) in the spinal cord of the same mouse with EAE at the peak of disease. **d**, Quantification of the flow cytometry data. Top, myeloid cell markers; bottom, polarization markers. *N* = 4 animals for the myeloid marker panel and 3 animals for the polarization marker panel. **e**, Scheme of the pooled CRISPR screen targeting 109 cytokine receptors and downstream signaling genes with 15 control sgRNAs. **f**, Cytokine receptors regulating CNS migration, determined by comparing the spinal cord to the bone marrow compartment. **g**,**h**, Cytokine receptors regulating M-iNOS (**g**) or M-Arg1 (**h**) polarization, determined by comparing the single iNOS^+^ (**g**) or Arg1^+^ (**h**) spinal cord macrophages to the iNOS and Arg1 double-negative macrophages. Two-way analysis of variance (ANOVA) with the two-stage linear step-up procedure of Benjamini, Krieger and Yekutieli for multiple comparisons, *F* = 1.98 × 10^−16^, *P* > 0.9999 used in **b**; multiple paired two-tailed *t*-tests or Wilcoxon tests with the two-stage linear step-up procedure of Benjamini, Krieger and Yekutieli for multiple comparisons used in **d**; NS, *P* value > 0.05; *P value < 0.05, **P value < 0.01, ***P value < 0.001, **** *P* value < 0.0001; figures show the mean ± sd; not all genes included in the screen are shown for **f**–**h**. Asterisks indicate *P* values and false discovery rate (FDR) < 0.05 and absolute log_2_(fold change) > 3 times the s.d. of noise distribution (Methods). Source data

**Extended Data Fig. 1:**
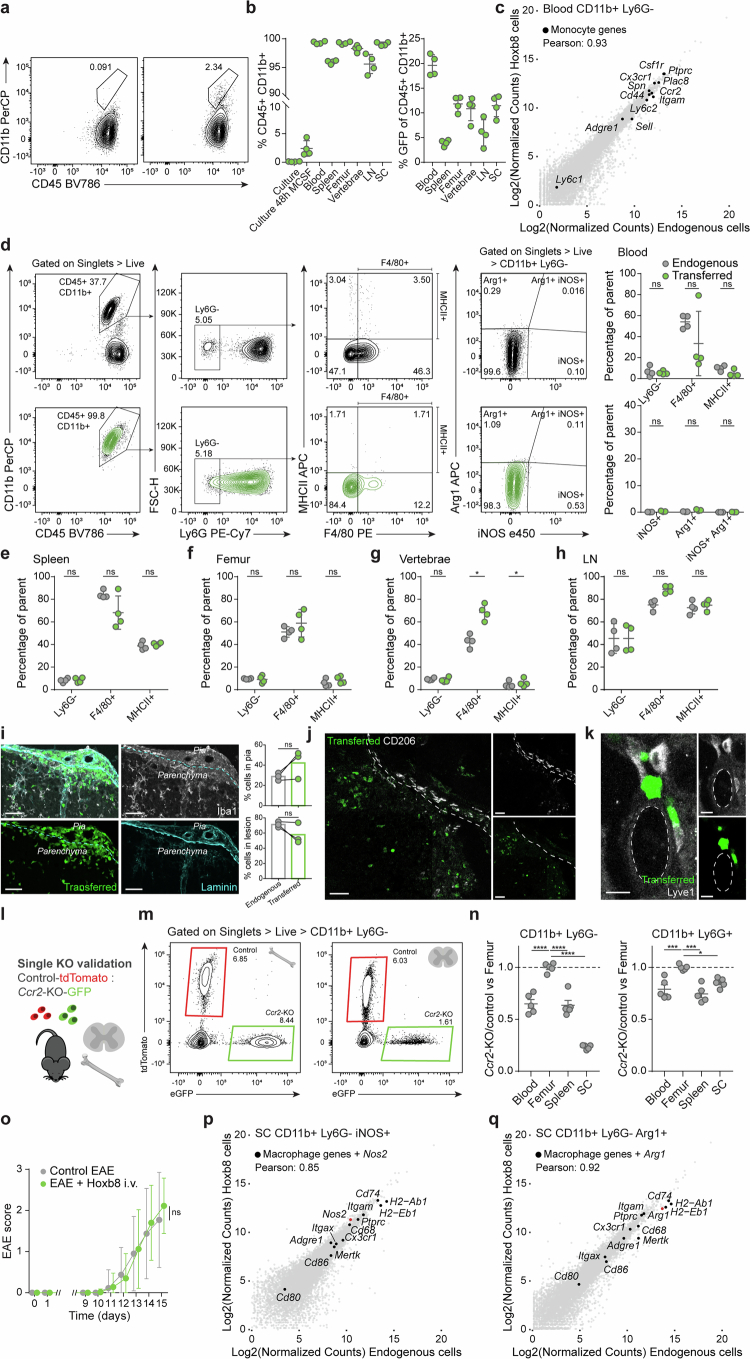
In vivo characterization of Hoxb8FL-derived myeloid cells. **a**, Representative flow cytometry plots of myeloid differentiation during the in vitro 48h treatment before i.v. injection. **b**, Percentage of myeloid Hoxb8FL-derived cells in vitro and in vivo (left) and quantification of the Hoxb8FL-derived myeloid cells in different tissue compartments in vivo. *N* = 4 animals **c**, Correlation of bulk RNA transcript counts in sorted Hoxb8FL-derived monocytes, compared to the same population of endogenous blood monocytes. *N* = 3 animals. **d**, Representative flow cytometry plots of the transferred Hoxb8FL-derived cells (bottom row, green) compared to endogenous cells (top row, gray) in the blood of the same mouse with EAE at the peak of disease, with two different panels (left) and quantification (right). *N* = 4 animals for the myeloid marker panel and three animals for the polarization marker panel. **e-h**, Percentage of Hoxb8FL-derived cells (green) and endogenous cells (gray) in the same animal in the spleen (**h**), femur bone marrow (**i**), vertebrae bone marrow (**j**) and inguinal draining lymph nodes (**k**), at the peak of EAE. *N* = 4 animals. **i**, Histological characterization of the distribution of Hoxb8FL-derived and endogenous myeloid cells in EAE lesions in the spinal cord pia matter and parenchyma, blue dashed line indicates pia-parenchyma separation. Right, percentage of total endogenous (gray) or Hoxb8FL (green) cells present in the pia (top) or parenchymal (bottom) compartments of the lesion. *N* = 3 animals, 3 sections per animal. Scale bar 100 μm. **j**, Representative image of the meningeal niche containing CD206+ resident macrophages but no infiltrated Hoxb8FL-derived myeloid cells. Dashed line indicates the dura. Scale bar 100 μm. **k**, Representative image of the perivascular niche containing Lyve+ resident macrophages but no infiltrated Hoxb8FL-derived myeloid cells. Dashed line indicates the vessel lumen. Scale bar 10 μm. **l**, Scheme of the experimental approach to study the migration phenotype of *Ccr2*-KO myeloid cells. **m**, Representative flow cytometry plots of control-tdTomato or *Ccr2*-KO GFP Hoxb8FL-derived myeloid cells in the bone marrow (left) or spinal cord (right) of a mouse with EAE at the peak of disease. **n**, Relative amounts of control-derived and *Ccr2*-KO-derived monocytes/macrophages or granulocytes across different tissues in mice with EAE at the peak of disease. *N* = 5 animals. **o**, EAE disease course of mice with (green) or without (gray) a Hoxb8FL cell transfer. *N* = 40 animals per group **p-q**, Correlation of bulk RNA transcript counts in sorted polarized Hoxb8FL-derived macrophages compared to the same population of endogenous spinal cord iNOS+ (p) or Arg1+ (q) macrophages at the peak of EAE. *N* = 3 animals. (**a**) Two-tailed paired t-test; (**d-h**) multiple two-tailed paired t-tests or Wilcoxon tests with the two-stage linear step-up procedure of Benjamini, Krieger and Yekutieli for multiple comparisons; (**n**) ordinary one-way ANOVA left *F* = 87.58 *P* < 0.0001, right *F* = 1.242, *P* = 0.3274; Kolmogorov-Smirnov test used in (**o**); ns p-value > 0.05, * p-value < 0.05, ** p-value < 0.01, *** p-value < 0.001, **** p-value < 0.0001; figures show mean ± sd. Source data

To dissect the cytokine signals that drive macrophage states in neuroinflammatory lesions, we established a new approach to perform pooled CRISPR knockout (KO) screens in monocyte-derived macrophages in vivo using Hoxb8FL cells, a hematopoietic progenitor line^20^ that can be easily expanded and gene edited in culture. We incubated hBCL2-overexpressing Hoxb8FL Cas9 cells transduced with an enhanced green fluorescent protein (eGFP) retrovirus (Hoxb8FL cells) in vitro with macrophage colony-stimulating factor (M-CSF) for 48 h to initiate myeloid differentiation, and then injected them intravenously (i.v.) into mice 8–9 days after immunization with MOG_1–125_ (Fig. 1a). This allowed the primed Hoxb8FL-derived cells to differentiate into CD45^+^CD11b^+^ myeloid cells in vivo before CNS lesions form and first experimental autoimmune encephalomyelitis (EAE) symptoms become apparent around 12 days after immunization. Indeed, Hoxb8FL-derived cells were detectable based on their eGFP expression in blood, spleen, lymph nodes and bone marrow (Extended Data Fig. 1b) and exhibited similar expression of monocyte genes in the blood, and of macrophage surface markers including F4/80 and major histocompatibility complex (MHC) class II in peripheral lymphoid tissues, as endogenous myeloid cells (Extended Data Fig. 1c–h). Notably, Hoxb8FL-derived CD11b^+^ cells also readily entered spinal EAE lesions in a CCR2-dependent manner where they made up about 15% of the CD11b^+^ cells and showed comparable distribution in the lesion to endogenous CD11b^+^ cells. The cell transfer did not affect the overall disease course (Fig. 1b and Extended Data Fig. 1i,l–o), and Hoxb8FL-derived myeloid cells did not contribute to the pool of resident meningeal and perivascular macrophages of the spinal cord (Extended Data Fig. 1j,k). In the EAE lesions, Hoxb8FL-derived myeloid cells polarized into iNOS^+^ and Arg1^+^ expressing cell populations, the transcriptomes of which closely correlated to the corresponding endogenous macrophage populations (Fig. 1c,d and Extended Data Fig. 1p,q).

### In vivo CRISPR screen identifies the essential cytokine signaling pathways that regulate migration and polarization of macrophages in MS models

To resolve the cytokine regulation of macrophage phenotypes in neuroinflammatory lesions, we performed a pooled CRISPR screen based on the transfer of Hoxb8FL-derived myeloid cells. We first constructed a CRISPR library of 343 single guide RNAs (sgRNAs), with three sgRNAs per gene and 15 non-targeting controls, to target 64 cytokine receptors and 45 of their downstream signaling mediators. We then transduced Hoxb8FL cells in vitro at a multiplicity of infection < 0.3, to ensure a single sgRNA transduced each cell. We selected infected cells, keeping the library coverage at a minimum of 100 cells containing each sgRNA (100× coverage). After in vitro pre-differentiation, we injected the cells into mice with EAE and about 6 days later, around the peak of EAE symptoms, we isolated Hoxb8FL-derived CD45 ^+^ CD11b^+^ myeloid cells from the bone marrow, and iNOS^−^ Arg1^−^, iNOS^+^ and Arg1^+^ Hoxb8FL-derived myeloid cells from the spinal cord by fluorescence-activated cell sorting (FACS; Fig. 1e). We then compared the relative distribution of sgRNAs across these cell populations as depletion of sgRNAs in a given population indicates an essential role for the respective targeted genes in the induction of the corresponding cell state. Indeed our results showed that: signaling through CCR2 alone was required for CNS entry of phagocytes (Fig. 1f); the interferon gamma (IFNγ) receptor (IFNGR) and tumor necrosis factor (TNF) receptors (TNFR) were required for iNOS^+^ polarization, which was decreased by signaling through the TGFβ receptor 1 (TGFBR1; Fig. 1g); while Arg1^+^ polarization was promoted by the TGFBR1 and granulocyte-macrophage colony-stimulating factor (GM-CSF) receptor (CSF2R), and inhibited by the IFNGR1 (Fig. 1h).

**Fig. 2:**
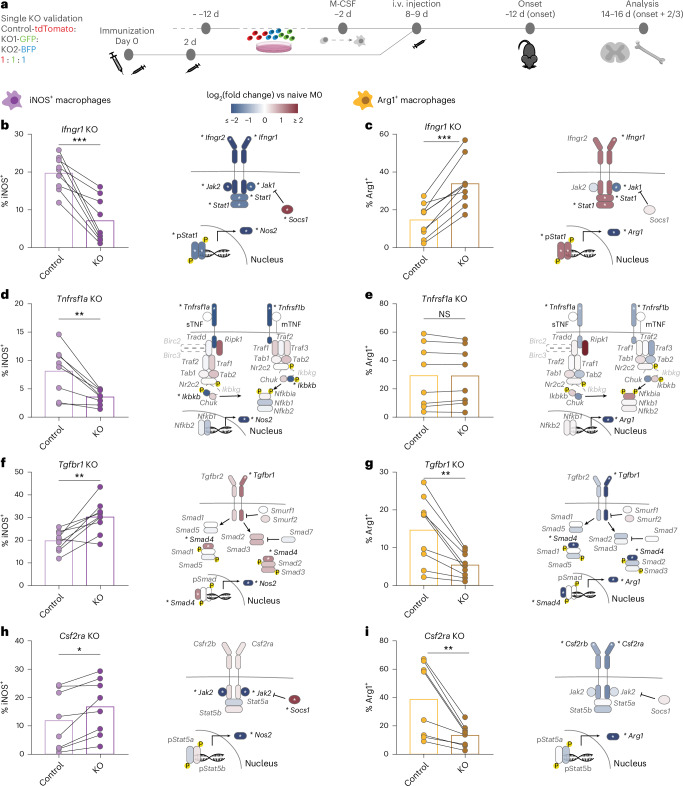
Validation of cytokine receptor effects on macrophage polarization and identification of their essential downstream mediators. **a**, Scheme of the validation experiment. Hoxb8FL control (tdTomato) cells were co-transferred with two different KO lines (GFP and blue fluorescent protein (BFP)) into the same immunized mouse before disease onset. **b**,**d**,**f**,**h**, Percentage of iNOS^+^ cell polarization in the single KO validation experiments compared to control, in the same animal (left) and scheme of the cytokine signaling pathway (right) for *Ifngr1*-KO (**b**), *Tnfrsf1a*-KO (**d**), *Tgfbr1*-KO (**f**) and *Csf2ra*-KO (**h**). The color code reflects the phenotype of M-iNOS polarization for the corresponding gene KO derived from the CRISPR screen shown in Fig. 1e–h. **c**,**e**,**g**,**i**, Percentage of Arg1^+^ cell polarization in the single KO validation experiments compared to control of the same animal (left) and scheme of the cytokine signaling pathway (right) for *Ifngr1*-KO (**c**), *Tnfrsf1a*-KO (**e**), *Tgfbr1*-KO (**g**) and *Csf2ra*-KO (**i**). The color code reflects the phenotype of M-Arg1 polarization for the corresponding gene KO derived from the CRISPR screen shown in Fig. 1e–h. **b**–**i**, *n* = 9 animals for *Ifngr1*-KO and *Tgfbr1*-KO transfer, *n* = 8 animals for *Tnfrsf1a*-KO and *Csf2ra*-KO transfer. Bar plots depict two-tailed paired *t*-test in **b**–**h** and a two-tailed paired Wilcoxon test in **i**. NS, *P* value > 0.05; *P value < 0.05, **P value < 0.01, ***P value < 0.001, ****P value < 0.0001. In **b**–**i**, asterisks indicate *P* values and FDR < 0.05 and absolute log_2_(fold change) > 3 times the s.d. of noise distribution (Methods). Source data

**Extended Data Fig. 2:**
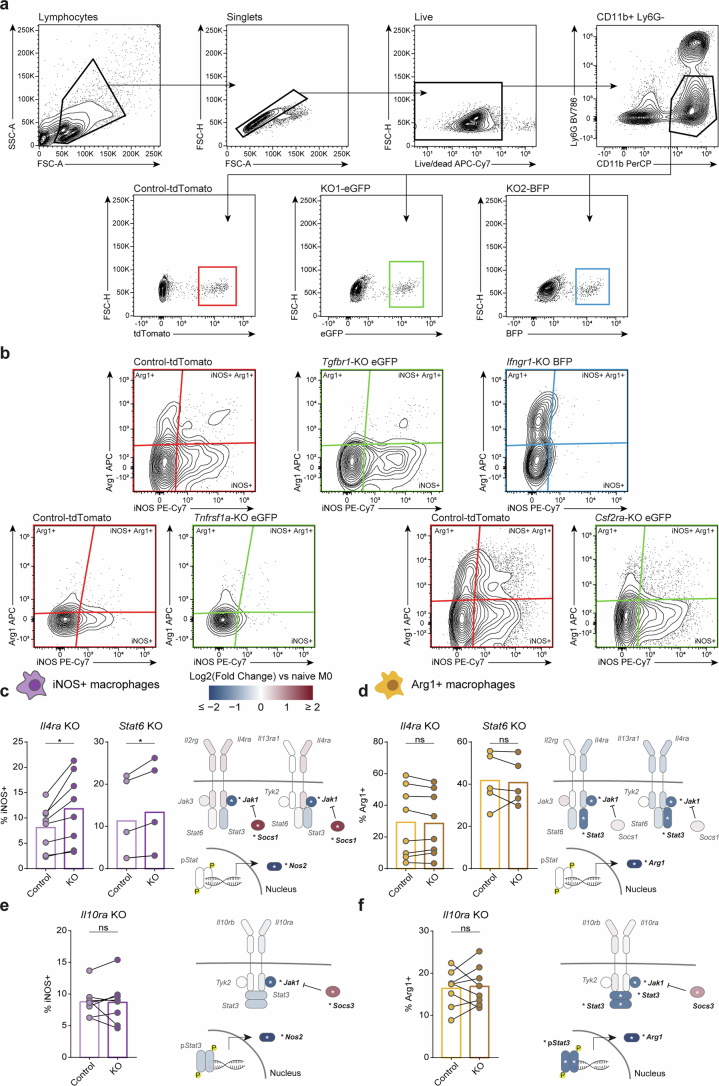
Validation of CRISPR screen results by single gene knock-out. **a**, Representative flow cytometry gating to assess gene KOs in Hoxb8FL-derived myeloid cells. **b**, Representative flow cytometry plots of myeloid cell polarization for *Ifngr1*-KO, *Tnfrsf1a*-KO, *Tgfbr1*-KO and *Csf2ra*-KO with their intra-animal control. **c-f**, Percentage of iNOS+ (**c, e**) and Arg1+ (**d, f**) cell polarization for *Il4ra*-KO and the downstream transcription factor *Stat6*-KO (**c, d**) and *Il10ra*-KO (**e, f**) compared to control (barplot). On the right, scheme of the cytokine signaling pathways, color-coded for the phenotype of the gene KO in M-iNOS (**c, e**) and M-Arg1 (**d, f**) polarization derived from the CRISPR screen shown in Fig. 1e. *N* = 8 animals for *Il4ra*-KO transfer, *n* = 5 animals for *Stat6*-KO transfer, *n* = 8 animals for *Il10ra*-KO transfer. Barplots two-tailed paired t-test in (**c-f**), ns p-value > 0.05, * p-value < 0.05, ** p-value < 0.01, *** p-value < 0.001, **** p-value < 0.0001; (**c-f**) schemes stars indicate p-value and fdr < 0.05 and absolute log2(Fold Change) > 3 x sd of noise distribution (see Methods for details). Source data

We confirmed these results by performing single-gene knockouts in Hoxb8FL cells and compared the polarization of Hoxb8FL-derived myeloid cells deficient for a given cytokine receptor with co-transferred control-edited Hoxb8FL-derived myeloid cells (Fig. 2a). These experiments confirmed that signaling via the IFNGR1 and the TNFR1 was required for iNOS^+^ polarization, while signaling through the TGFBR1 and the CSF2RA promoted Arg1^+^ polarization (Fig. 2b–i and Extended Data Fig. 2a,b). Single-gene knockouts further confirmed that cytokines including interleukin (IL)-4 and IL-10, which induce Arg1^+^ polarization in vitro^21^, are not required for Arg1^+^ polarization in vivo, at least in this model at this disease time point (Extended Data Fig. 2c–f). Next, we leveraged our CRISPR screen to define the downstream signaling pathway components that are essential for transducing cytokine effects on macrophage polarization. We found that JAK1, JAK2 and STAT1 are required to mediate the effects of IFNGR, while IKK2 (*Ikbkb*), the key kinase mediating canonical nuclear factor-κB activation, is the only essential component of TNFR1 downstream signaling; moreover, only the transcription factor SMAD4 is essential for mediating the effects of TGFBR1 on both iNOS^+^ and Arg1^+^ polarization (Fig. 2b–i). Taken together, four cytokine receptors, IFNGR1, TNFR1, TGFBR1 and CSF2RA, and defined components of their downstream signaling pathways, are essential for establishing iNOS^+^ and Arg1^+^ macrophage polarization in neuroinflammatory lesions in the active MOG EAE model.

**Extended Data Fig. 3:**
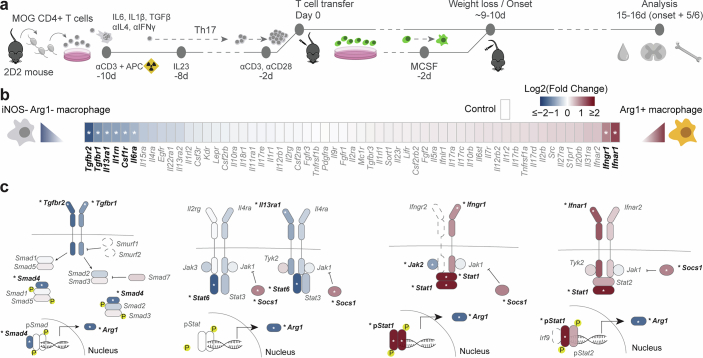
In vivo CRISPR screen of macrophage polarization in a Th17-transfer EAE model. **a**, Scheme of the Hoxb8FL cell transfer in the Th17 T cell adoptive transfer EAE model. **b**, Cytokine receptors affecting M-Arg1 polarization, determined by comparing the Arg1+ spinal cord macrophages (13–29% of total Hoxb8FL-derived macrophages, *n* = 2 experiments) to the iNOS and Arg1 double negative macrophages (71–87% of total Hoxb8FL-derived macrophages, *n* = 2 experiments). **c**, Schemes showing the signaling pathways of the cytokines driving M-Arg1 polarization in the Th17 EAE model with pathway members color coded for the phenotype of the gene KO in M-Arg1 polarization in the CRISPR screen. Stars in (**b, c**) indicate p-value and fdr < 0.05 and absolute log2(Fold Change) > 3 x sd of noise distribution (see Methods for details). Source data

To determine which of these cytokine regulation pathways were unique to this particular MS model and which might be more universal, we repeated the screen in a passive EAE model induced by the transfer of MOG-reactive IL-17-producing helper T (T_H_17) cells. We transferred CRISPR-edited Hoxb8FL cells at the onset of disease symptoms and isolated Arg1^+^ and Arg1^−^ Hoxb8FL-derived macrophages 6 days later as at this time point during lesion resolution most macrophages were Arg1^+^ (Extended Data Fig. 3a).

Our results showed that—as in the active MOG EAE model—TGFBR supported Arg1^+^ polarization, while IFNGR1 reduced Arg1^+^ polarization; similarly, the effects of TGFBR were mediated by the transcription factor SMAD4, while the effects of IFNGR1 relied on STAT1. However, in contrast to the MOG EAE model, Arg1^+^ polarization in the T_H_17 transfer model was not affected by CSF2R deletion but instead was limited by interfering with IL-4/IL-13 signaling, at the level of either IL13RA or the transcription factor STAT6. Furthermore, in the T_H_17 transfer EAE model, Arg1^+^ polarization was also inhibited by type I interferon signaling mediated by IFNAR1 (Extended Data Fig. 3b,c). Taken together, our findings indicate that IFNγ and TGFβ reciprocally regulate macrophage polarization across MS models while the regulatory effects of other cytokines—among them GM-CSF and IL-4/IL-13–depend on the inflammatory context and thus might differ between lesion types and/or disease stages.

### Perturb-seq analysis of Hoxb8FL-derived and bone-marrow chimeric macrophages in neuroinflammatory lesions reveals cytokine regulation of myeloid cell differentiation at the single-cell level

**Extended Data Fig. 4:**
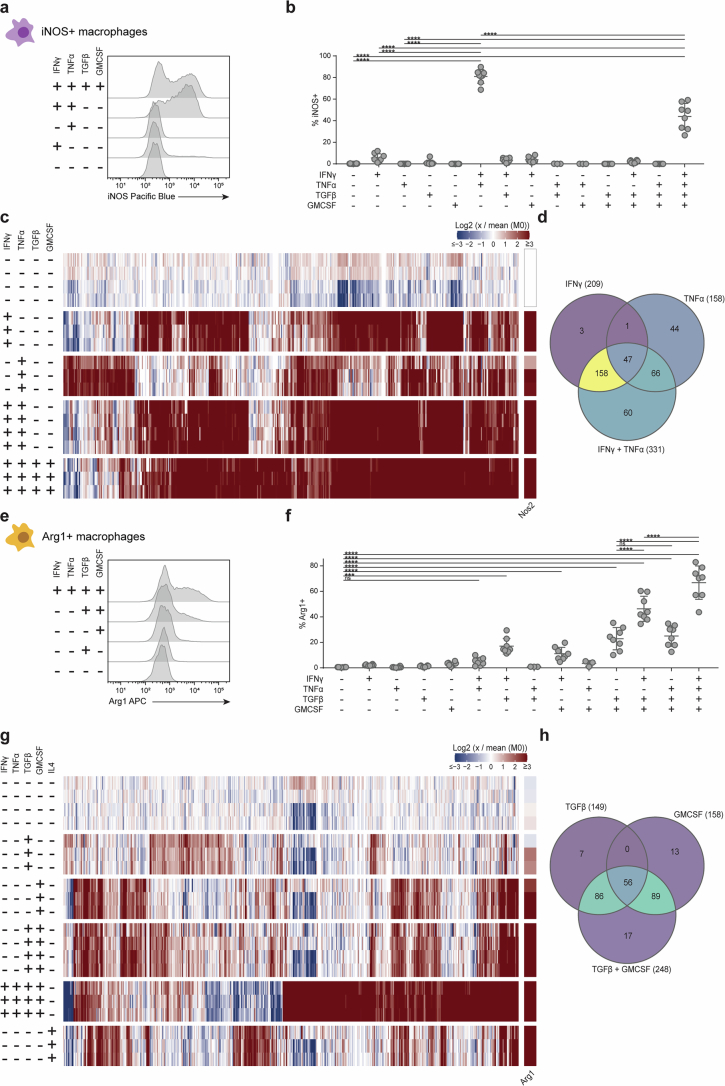
Essential regulatory cytokines act synergistically to induce macrophage polarization in vitro. **a, e**, Representative flow cytometry plots of iNOS (**a**) or Arg1 (**e**) induction in bone-marrow derived macrophages (BMDMs) in different cytokine incubation conditions. **b, f**, Percentage of iNOS (**b**) or Arg1 (**f**) positive cells among BMDMs in different cytokine incubation conditions. *N* = 8 in vitro experiments, except for conditions TNFα + TGFβ and TNFα + GMCSF which have *n* = 3. **c, g**, Heatmap of genes induced by different cytokine combinations in BMDMs during M-iNOS (**c**) or M-Arg1 (**g**) polarization across biological replicates, color coded for the log2(Fold Change) value of the replicate vs the mean of the naïve M0 macrophages. Naïve macrophage and four cytokine combination samples are repeated in both plots, showing different genes. Only the genes significantly upregulated in any of the shown cytokine combinations compared to naïve macrophages are included on the heatmap. *N* = 3–4 experiments. **d, h**, Venn diagram of the overlap in differentially regulated genes induced in BMDMs by incubation with the listed cytokines compared to naïve M0 macrophages. In brackets, total number of DEGs per cytokine condition. Purple to yellow color code indicates increasing numbers of differentially regulated genes. Ordinary one-way ANOVA with the two-stage linear step-up procedure of Benjamini, Krieger and Yekutieli for multiple comparisons used in (**b, f**); (**b**) *F* = 244.6, *P* < 0.0001, (**f**) *F* = 77.85, *P* < 0.0001; ns p-value > 0.05, * p-value < 0.05, ** p-value < 0.01, *** p-value < 0.001, **** p-value < 0.0001; figures show mean ± sd; Significantly upregulated genes in (**c, g**) were defined as having an adjusted p-value < 0.05 and log2(Fold Change) > 3 x standard deviation of all log2(Fold Change) in the comparison. Source data

To obtain a higher resolved understanding of these essential cytokine regulators, we first generated bone-marrow-derived macrophages, exposed them to different combinations of cytokines in vitro, and subjected them to transcriptional analysis (Extended Data Fig. 4). As predicted by our screen results, IFNγ and TNF acted synergistically to induce iNOS^+^ polarization, while TGFβ and GM-CSF collaborated during the induction of Arg1^+^ polarization: however, these cytokines did so by inducing distinct, cytokine-specific transcriptional modules (Extended Data Fig. 4). Notably, the highest proportion of Arg1^+^ cells was induced by the combination of all four cytokines, which may be related to the observation that myeloid cells can transition between M^iNOS^ and M^Arg1^ states in vivo and in vitro^8^.

**Fig. 3:**
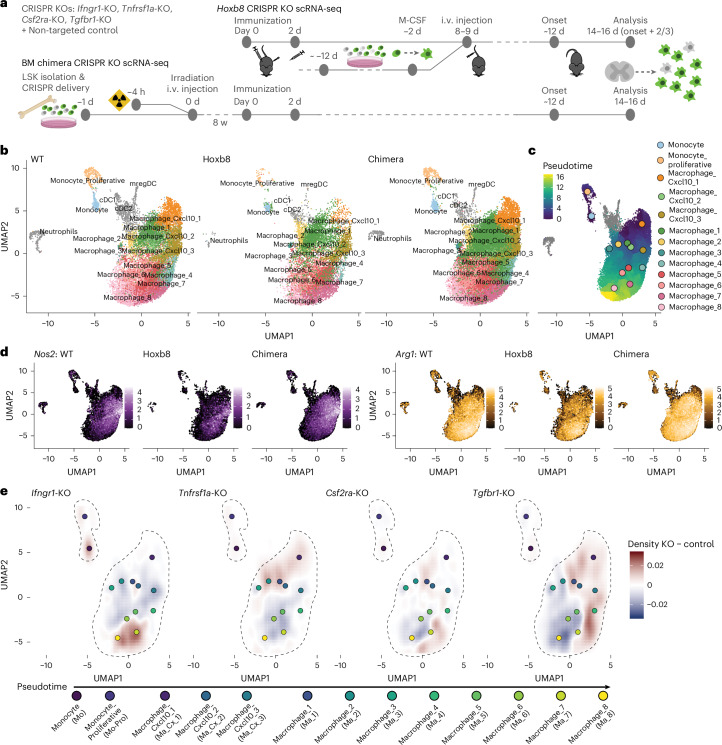
Pertub-seq reveals cytokine regulation of macrophages at the single-cell level. **a**, Scheme of the experimental design for the Hoxb8FL-derived macrophage and the bone-marrow chimera Perturb-seq experiments. Hoxb8FL (top) or LSK (bottom) cells deficient for the receptors of the four essential cytokines driving macrophage polarization in EAE as well as control-edited cells were either transferred as a Hoxb8FL cell pool, as previously described (top), or used to reconstitute the immune system after irradiation in a bone-marrow chimera model (bottom). At the peak of disease, endogenous (not shown on scheme), Hoxb8FL-derived or chimeric macrophages were harvested from the spinal cord and subjected to single-cell transcriptional sequencing. **b**, UMAP plots of the three EAE macrophage single-cell sequencing datasets after integration. Left, endogenous macrophages from WT EAE; middle, Hoxb8FL-derived macrophages; right, chimeric macrophages. **c**, Pseudotime across the different monocyte and macrophage clusters in the integrated dataset. Clusters are color coded according to the scale on the right. **d**, Feature plot of *Nos2* and *Arg1* expression in the three datasets; expression levels are color coded according to the scales on the right of the plot. **e**, Density difference plot showing differences in the distribution of myeloid cell subpopulations in the KOs compared to the control cells; density differences are color coded according to the scale on the right.

**Extended Data Fig. 5:**
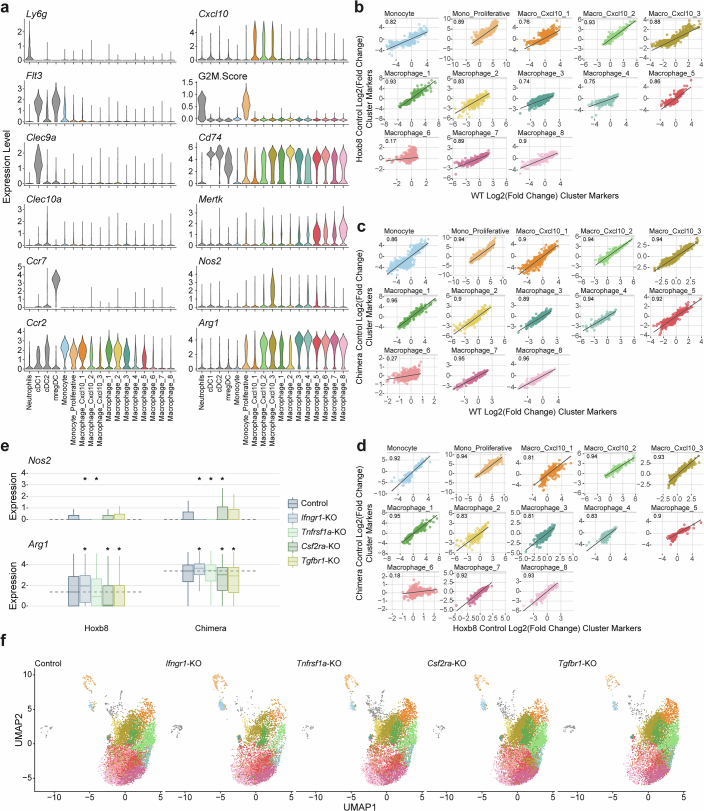
Hoxb8FL-derived and bone-marrow-chimera-derived macrophages show similar transcriptional regulation to endogenous macrophages in neuroinflammatory lesions. **a**, Expression of key genes determining cluster cell identity in the scRNAseq experiments across clusters. **b-d**, Correlation of the gene log2(Fold Change) of each cluster vs all the other clusters between the WT EAE macrophage experiment clusters and the Hoxb8FL-derived macrophage experiment clusters (**b**), the WT EAE macrophage experiment clusters and the chimeric macrophage experiment clusters (**c**) and the Hoxb8FL-derived macrophage experiment clusters and the chimeric macrophage experiment clusters (**d**). **e**, *Nos2* (top) and *Arg1* (bottom) regulation across KOs in the Hoxb8FL-derived (left) and chimeric (right) macrophages compared to control cells of the same experiment. Boxplot line shows median, box Q1 and Q3, and whiskers the interquantile range x 1.5. Dashed line shows control median. **f**, UMAP plots of control-edited and cytokine receptor deficient Hoxb8FL-derived and bone-marrow chimeric myeloid cells (**b-d**), Top left values indicate Pearson correlation; (**e**), stars indicate adjusted p-value < 0.05 in the KO vs control comparison.

Therefore, we next aimed to uncover the effect of these cytokines on macrophage differentiation and molecular specification in vivo. To do so, we conducted three separate single-cell RNA-sequencing (scRNA-seq) experiments on CD11b^+^Ly6G^−^ monocytes/macrophages isolated at the peak of disease from mice with active MOG EAE: (i) a wild-type (WT) EAE experiment as reference; (ii) Hoxb8FL-derived CRISPR KO experiments targeting the IFNGR1, TNFR1, TGFBR1 and CSF2RA; and (iii) bone-marrow chimera experiments having reconstituted the bone marrow with individual CRISPR KOs of the same receptors (Fig. 3a and Methods). When we integrated the transcriptional data from the three sets of experiments, we found that the same myeloid cell clusters were present in all of them; moreover, cells in the same clusters exhibited high transcriptional similarity independent of their origin (Fig. 3b and Extended Data Fig. 5a–d). Through analysis of marker gene expression and pseudotime analysis of the integrated object (Fig. 3c), we assigned the myeloid cell populations from monocyte-like cells to three clusters of CXCL10^+^ macrophages^22^ (‘macrophages CXCL10 1–3’) and eight clusters of other macrophages (‘macrophages 1–8’). To relate the single-cell analysis to our CRISPR screen, we mapped the expression of our screen markers *Nos2* and *Arg1* in the scRNA-seq data and found that the populations expressing these genes were consistent across experiments and remained mostly distinct from each other: *Nos2* expression was more restricted and primarily found in early-to-intermediate phenotypes (based on pseudotime), most prominently in the cluster ‘macrophages CXCL10 3’; while *Arg1* was broadly expressed in intermediate and later macrophage populations (Fig. 3d and Extended Data Fig. 5a). Furthermore, both Hoxb8FL-transfer and bone-marrow chimera-based CRISPR editing experiments confirmed the effects of cytokine receptor deletion on iNOS^+^ and Arg1^+^ polarization on the transcriptomic level as predicted by our initial CRISPR screen results (Extended Data Fig. 5e).

Next, we investigated how the lack of IFNγ, TNF, GM-CSF or TGFβ signaling in neuroinflammatory lesions affects the differentiation of myeloid cell subpopulations. We compared the distribution of monocyte/macrophage cell clusters in the uniform manifold approximation and projection (UMAP) between the individual KOs and the control-edited cells in both Hoxb8FL-transfer and bone-marrow chimera experiments. Among them only the deletion of IFNGR1 affected the monocyte-like cell phenotypes and resulted in a relative increase in the proportion of the monocyte population, as previously observed^23^. Among the macrophage clusters, deletion of IFNGR1 and TNFR1 showed opposite phenotypes, with IFNγ signaling needed for maintaining earlier—presumably more pro-inflammatory—macrophage clusters, and TNF signaling being required for progression to later macrophage cell phenotypes (Fig. 3e). In contrast, deletion of TGFBR1 and CSF2RA showed overlapping effects on the distribution of macrophage subpopulations, with both cytokines blocking the induction of CXCL10^+^ macrophages and promoting the differentiation of macrophages to the later macrophage cell phenotypes (Fig. 3e).

### Essential cytokines differentially regulate lesion-promoting and lesion-resolving actions of myeloid cells in neuroinflammatory lesions

**Fig. 4:**
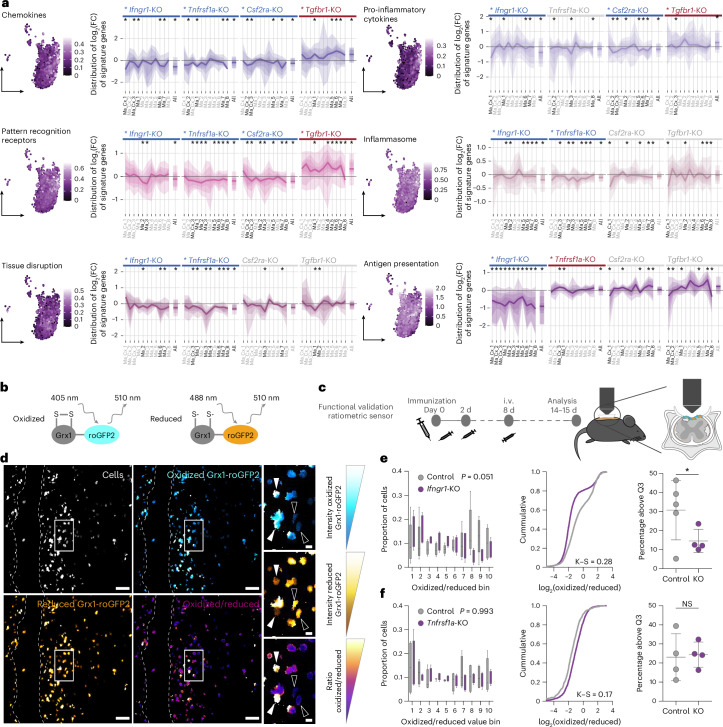
Deletion of cytokine receptors differentially regulates lesion-promoting properties of macrophages in neuroinflammation. **a**, Left, expression of each transcriptional signature related to lesion-promoting properties in Hoxb8FL control cells; right, phenotype of the KOs in Hoxb8FL-derived cells for the respective signature across macrophage clusters (Ma_Cx_1 to Ma_8) and overall (All) compared to the control cells. The shading/lines represent the distribution of log_2_(fold change or FC) of the KO compared to the control for all the genes that make up the signature, with the thick line being the median (quantile 50) log_2_(fold change), and the thinner overlaid shadows representing the lines of the adjacent quantiles: 40th and 60th quantiles of the log_2_(fold change) delimit the darkest shadow flanking the median line, in steps of 10 until the 10th and 90th quantiles delimiting the lightest shadow at the edges. For statistical analysis, gene-set enrichment analysis (GSEA) was used (Methods). **b**, Scheme illustrating the detection of oxidized/reduced cellular states by excitation ratiometric imaging of the Grx1-roGFP2 sensor. **c**, Experimental design of the intravital imaging experiments based on the transfer of Hoxb8FL-derived myeloid cells expressing the Grx1-roGFP2 sensor. **d**, Representative images of the dorsal lumbar spinal cord along the midline vein, showing a neuroinflammatory lesion infiltrated by Hoxb8FL-derived myeloid cells expressing the Grx1-roGFP2 sensor. Filled white arrowheads indicate myeloid cells in an oxidized state, while outlined arrowheads indicate myeloid cells in a reduced state. Scale bars, 50 μm (overview image) and 10 μm (inset). **e**,**f**, Quantification of the redox state of *Ifngr1*-KO (**e**) or *Tnfrsf1a-*KO (**f**) Hoxb8FL-derived myeloid cells expressing the Grx1-roGFP2 sensor. Left, proportion of control (gray) or KO (purple) cells per animal that fall in each oxidation/reduction Grx1-roGFP2 ratio bin, where lower bin numbers mean low ratio; the whole range is divided into ten bins and each bin is equally sized; middle, cumulative distribution of the oxidized/reduced Grx1-roGFP2 ratios of all cells; right, percentage of cells per animal that show an oxidized/reduced ratio above the 75th percentile ratio of their experiment. *N* = 5 control and 4 *Ifngr1*-KO animals from two independent experiments; control, 883, 587, 95, 172 and 677, and *Ifngr1-*KO, 2,394, 1,585, 96 and 99 cells per animal analyzed (**e**); *N* = 4 control and 4 *Tnfrsf1a*-KO from two different independent experiments; control, 33, 128, 36 and 342, and *Tnfrsf1a-*KO, 145, 1612, 123 and 828 cells per animal analyzed (**f**). K–S, Kolmogorov–Smirnov statistic. In the box plots, the line shows median, the box extends from the first quartile (Q1) to the third quartile (Q3), and the whiskers extend to the smallest and largest values within 1.5 times the interquartile range from Q1 and Q3. In **a**, KO names marked by an asterisk at the top of the plots and black ‘All’ labels indicate that the phenotype of the signature for that KO versus control in the global Hoxb8FL-derived cells is significant. Blue indicates downregulated and red indicates upregulated in the KO compared to the control. Asterisks and black cluster name labels indicate the phenotype is significant for that cluster for the Hoxb8FL-derived KO versus the control. Significance was determined with the GSEA pathway analysis algorithm for a nominal (NOM) *P* value < 0.05 or an FDR *q* value < 0.25 and absolute normalized enrichment score (NES) > 1.5 (Methods); **e**,**f**, (left), Ordinary two-way ANOVA with a single pooled variance, *F* = 2.009, *P* = 0.0509 (**e**) and *F* = 0.2027, *P* = 0.9929 (**f**) for the interaction of genotype × bin. **e**,**f** (middle) K–S statistic. **e**,**f**, (right) one-tailed unpaired *t*-test. NS, *P* value > 0.05, *P value < 0.05, **P value < 0.01, ***P value < 0.001, ****P value < 0.0001; figures show the mean ± s.d. Source data

**Extended Data Fig. 6:**
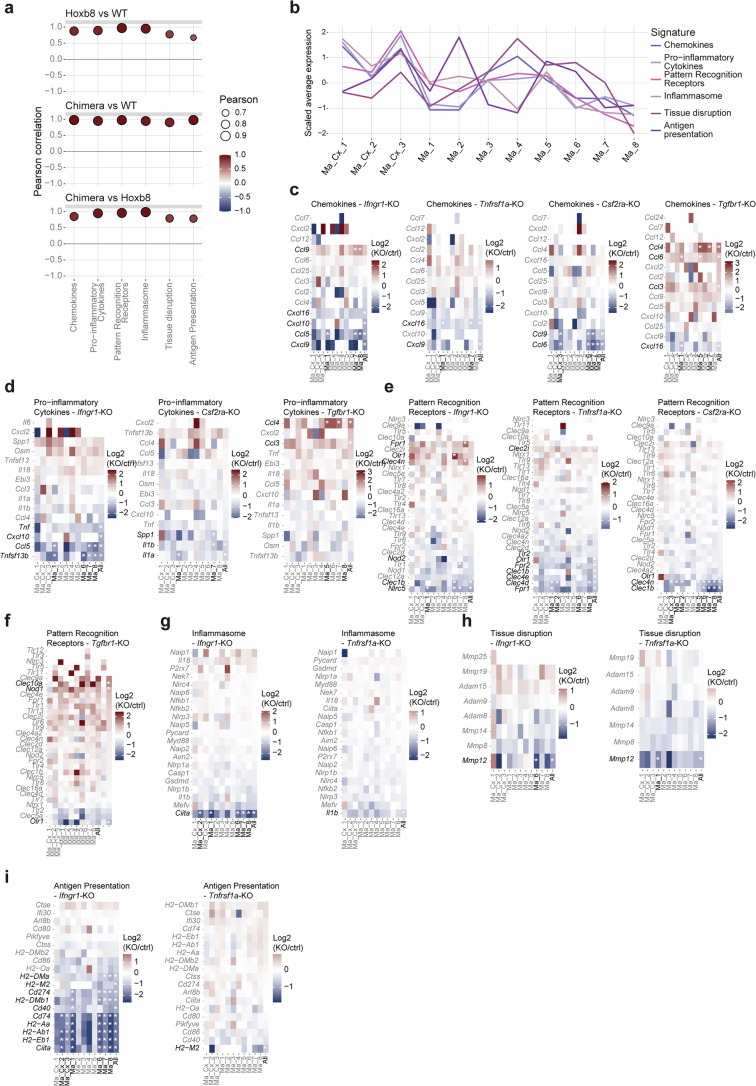
Individual genes related to lesion-promoting macrophage actions are differentially regulated by deletion of cytokine receptors. **a**, Pearson correlation of the individual signatures’ average gene expression across clusters between Hoxb8FL control and WT EAE macrophages (top), chimeric control and WT EAE macrophages (middle), and chimeric control and Hoxb8FL control macrophages (bottom). **b**, Expression pattern of curated transcriptional signatures related to lesion-promoting properties of macrophages across macrophage clusters derived from Hoxb8FL control-edited cells. **c-i**, Heatmaps of the log2(Fold Change) of individual genes comparing Hoxb8FL-derived KO cells to control, for each transcriptional signature in which the corresponding KO has a significant phenotype (as shown in Fig. 5). Stars in (**c-i**) indicate adjusted p-value < 0.05 and absolute log2(Fold Change) > 3 x standard deviation of the log2(Fold Change) distribution of the cluster as analyzed by Seurat FindMarkers. The genes are arranged from highest to lowest log2(Fold Change) in the overall KO vs control comparison, so their order changes between plots of the same signature. Source data

**Extended Data Fig. 7:**
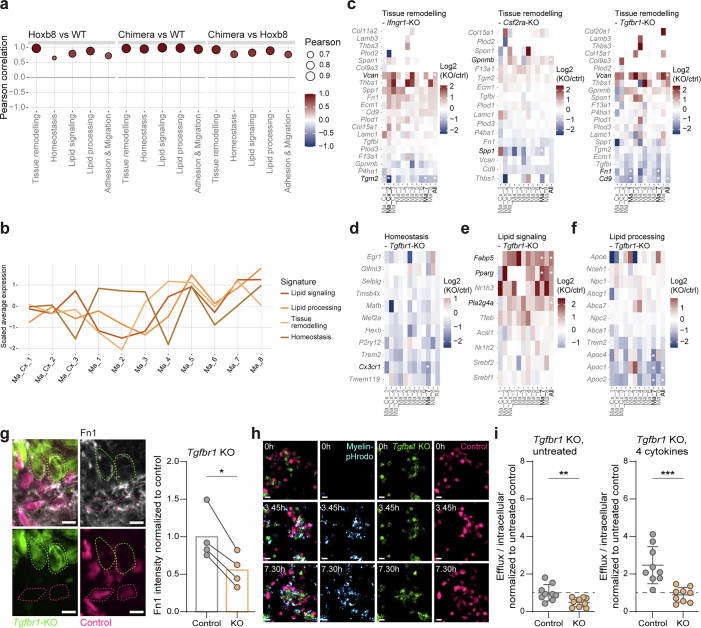
Individual genes related to lesion-resolving macrophage actions are differentially regulated by deletion of cytokine receptors. **a**, Pearson correlation of the individual signatures’ average gene expression across clusters between Hoxb8FL control and WT EAE macrophages (top), chimeric control and WT EAE macrophages (middle), and chimeric control and Hoxb8FL control macrophages (bottom). **b**, Expression pattern of curated transcriptional signatures related to lesion-resolving properties of macrophages across macrophage clusters derived from Hoxb8FL control-edited cells. **c-f**, Heatmaps of the log2(Fold Change) of individual genes comparing Hoxb8FL-derived KO cells to control, for each transcriptional signature in which the corresponding KO has a significant phenotype (as shown in Fig. 6). **g**, Left, representative image of fibronectin (Fn1) immunolabeling in control-edited and *Tgfbr1*-KO Hoxb8FL-derived myeloid cells in EAE lesions at peak of disease. Scale bars are 10 μm. Right, quantification. *N* = 4 animals. **h**, Representative images of myelin-pHrodo uptake over time by control-edited or *Tgfbr1*-KO Hoxb8FL-derived macrophages in vitro. Scale bars are 10 μm. **i**, Quantification of cholesterol-TMR in the supernatant of untreated (left) or IFNγ, TNFα, GM-CSF and TGFβ treated (right) control or *Tgfbr1*-KO Hoxb8FL-derived macrophages. *N* = 3 independent experiments with 3 technical replicates each, data plotted from the technical replicates normalized to untreated control average. (**c-f**) Stars indicate adjusted p-value < 0.05 and absolute log2(Fold Change) > 3 x standard deviation of the log2(Fold Change) distribution of the cluster as analyzed by Seurat FindMarkers. The genes are arranged from highest to lowest log2(Fold Change) in the overall KO vs control comparison, so their order changes between plots of the same signature. (**g**) two-tailed paired t-test; (**i**) two-tailed unpaired t-test; ns p-value > 0.05, * p-value < 0.05, ** p-value < 0.01, *** p-value < 0.001, **** p-value < 0.0001; figures show mean ± sd. Source data

To further characterize the effects of the essential cytokine regulators on the molecular specification of macrophages, we next manually curated gene signatures that represent distinct lesion-promoting and lesion-resolving properties of macrophages (Methods). In line with the transcriptional similarity of macrophage clusters across experimental settings, these signatures were highly conserved between endogenous macrophages and macrophages derived from bone-marrow chimera or Hoxb8FL-progenitor cells (Extended Data Figs. 6a and 7a). When we investigated the expression of these signatures in our single-cell transcriptomic data, we found that the expression of signatures related to lesion-promoting functions across macrophage clusters was highly correlated, and decreased from early to later macrophage populations in neuroinflammatory lesions (Extended Data Fig. 6b). We next assessed how each cytokine receptor deletion affected the expression of the gene signatures in each of the individual macrophage clusters. Our results showed that overall lesion-promoting actions of macrophages are underpinned by IFNGR1, TNFR1 and CSF2RA signaling, and inhibited by TGFBR1 signaling—with the exception of ‘antigen presentation’, which primarily depends on IFNGR1 signaling (Fig. 4a and Extended Data Fig. 6c–i). To further investigate the effect of these cytokine regulators on a defined lesion-promoting function of myeloid cells, we focused on the regulation of oxidative bursting, a key contributor to tissue damage in experimental and human neuroinflammatory lesions^9,24–26^. To directly read out the redox state of myeloid cells, we expressed the Grx1-roGFP2 sensor^27^ in CRISPR-edited Hoxb8FL-derived myeloid cells. Our results showed that in neuroinflammatory lesions in vivo IFNγ signaling appears to be a primary driver of oxidative bursting (Fig. 4b–f).

**Fig. 5:**
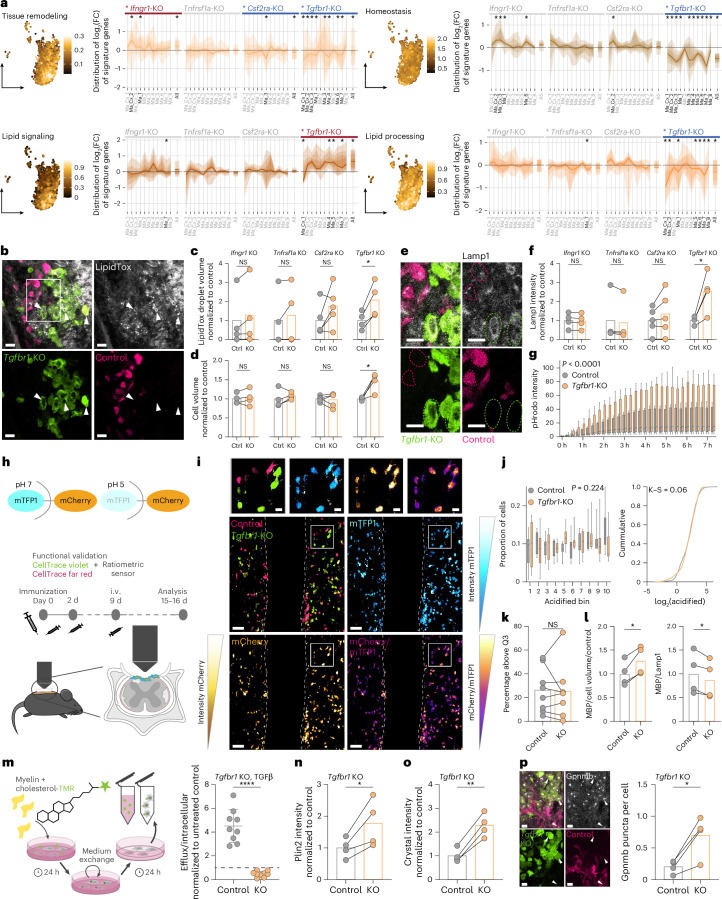
Deletion of cytokine receptors differentially regulates lesion-resolving properties of macrophages in neuroinflammation. **a**, Same representation as in Fig. 4a for lesion-resolving gene signatures. **b**,**e**, Representative image of neutral-lipid loaded (**b**) or Lamp1-positive (**e**) control and *Tgfbr1*-KO Hoxb8FL-derived myeloid cells in EAE lesions at peak of disease. Scale bars, 10 μm; white arrows indicate macrophages with a foamy appearance (**b**); inset indicates area in **e**. **c**,**d**,**f**, Quantification of neutral-lipid droplet volume (**c**), cell volume (**d**) and Lamp1 intensity (**f**) in control (gray) and cytokine receptor KO (orange) Hoxb8FL-derived myeloid cells. *N* = 4 animals for *Ifngr1*-KO, *Tnfrsf1a*-KO and *Tgfbr1*-KO, *n* = 5 animals for *Csf2ra*-KO. **g**, Quantification of pHrodo intensity over time in vitro upon pHrodo-myelin treatment of control and *Tgfbr1*-KO Hoxb8FL-derived macrophages. *N* = 3 independent in vitro experiments with 6 technical replicates per experiment. Data are plotted from all technical replicates. In the box plots, the line shows median, the box extends from Q1 to Q3, and the whiskers extend to the smallest and largest values within 1.5 times the interquartile range from Q1 and Q3. **h**, Scheme illustrating the detection of lysosomal acidification using the mTFP1-mCherry sensor and the experimental design of the intravital imaging experiment co-transferring dye-labeled mTFP1-mCherry Hoxb8FL control-edited or *Tgfbr1*-KO cells. **i**, Representative image of the dorsal lumbar spinal cord along the midline vein, showing a neuroinflammatory lesion infiltrated by Hoxb8FL-derived dye-labeled control-edited and *Tgfbr1*-KO myeloid cells expressing mTFP1-mCherry. Scale bars, 50 μm (overview image) and 10 μm (inset). **j**,**k**, Quantification of the lysosome acidification state in Hoxb8FL-derived myeloid cells expressing mTFP1-mCherry. **j**, Left, proportion of control (gray) or *Tgfbr1*-KO (orange) myeloid cells per animal that fall in each acidification mCherry/mTFP1 ratio bin, where lower bin numbers mean a low ratio, the whole range is divided into ten bins and each bin is equally sized; right, cumulative distribution of the acidification mCherry/mTFP1 ratio of all cells. **k**, Percentage of cells per animal that have an acidification ratio above the 75th percentile ratio of their experiment. **j**,**k**, *N* = 8 animals; control, 71, 167, 68, 369, 21, 1,179, 142 and 624; and *Tgfbr1-*KO, 57, 55, 30, 205, 460, 115, 486 and 365 cells per animal analyzed. In the box plots, the line shows median, the box extends from Q1 to Q3, and the whiskers extend to the smallest and largest values within 1.5 times the interquartile range from Q1 and Q3. **l**,**n**, Quantification of MBP absolute intensity (**l**, left), MBP intensity normalized to Lamp1 intensity (**l**, right) or Plin2 intensity (**n**) in Hoxb8FL-derived control (gray) or *Tgfbr1*-KO (orange) myeloid cells in EAE lesions at the peak of disease. *N* = 4 animals. **m**, Scheme of the cholesterol efflux assay (left) and quantification of cholesterol-TMR in the supernatant of TGFβ-treated control or *Tgfbr1*-KO Hoxb8FL-derived macrophages*. N* = 3 independent experiments with 3 technical replicates each; data are plotted from the technical replicates normalized to untreated control average. **o**, Quantification of the presence of cholesterol crystals by confocal reflection microscopy in control or *Tgfbr1*-KO Hoxb8FL-derived myeloid cells in EAE lesions at the peak of disease. *N* = 4 animals. **p**, Representative images showing Gpnmb labeling in control and *Tgfbr1*-KO Hoxb8FL-derived myeloid cells in EAE lesions at the peak of disease. Scale bars, 10 μm. White arrowheads indicate Gpnmb puncta. Right, quantification. *N* = 4 animals. **a**, See Fig. 4a for details; **c**, **d**, **f**, **k**, **l**, **m**, **o** and **p**, Two-tailed paired *t*-test or Wilcoxon test; **g** and **j**, ordinary two-way ANOVA with a single pooled variance. **g**, *F* = 55.05, *P* < 0.0001 for the genotype. **j**, *F* = 1.336, *P* = 0.2237 for the interaction of genotype × bin; **j** (right), K–S statistic. **n**, Two-tailed unpaired *t*-test; NS, *P* value > 0.05; *P value < 0.05, **P value < 0.01, ***P value < 0.001, ****P value < 0.0001; figures show the mean ± sd. Source data

In line with these results and the antagonistic actions of IFNγ and TGFβ signaling observed in the CRISPR screens across MS models, transcriptional signatures related to lesion-resolving actions of macrophages were primarily induced by TGFBR1 signaling and often inhibited by IFNGR1 signaling. Notably, both TGFBR1 signaling and CSF2RA signaling appear to drive the expression of genes associated with ‘tissue remodeling’, indicating that—as observed in the CRISPR screens and for macrophage polarization in vitro—CSF2RA receptor signaling can synergize with TGFBR1 signaling in distinct functional domains (Fig. 5a and Extended Data Fig. 7b–f). Furthermore, we observed prominent effects of TGFBR1 ablation on transcriptional signatures related to lipid signaling and lipid processing. As clearing the lipid-rich myelin debris is an important prerequisite for the resolution of neuroinflammatory lesions, we further investigated how essential cytokine regulators affect lipid uptake and digestion. In line with the regulation of related transcriptional signatures, we observed that specifically Hoxb8FL-derived myeloid cells lacking TGFBR1 but not those lacking IFNGR1, TNFR or CSF2RA showed an increase in cell size, lipid content and lysosomal marker expression (Fig. 5b–f). Notably, our subsequent analysis revealed that this lipid accumulation is not related to an impairment of phagocytosis or lysosomal acidification but rather due to an alteration of the lipid efflux capacity of myeloid cells that appear to depend on TGFβ signaling (Fig. 5g–m and Extended Data Fig. 7h,i). In line with these results, TGFBR1-deficient Hoxb8FL-derived myeloid cells showed an increased content of lipid droplets (Fig. 5n) and cholesterol crystals (Fig. 5o) that can drive a pro-inflammatory foamy cell phenotype^28^. Indeed, myeloid cells lacking TGFBR1 showed an increased expression of the foamy cell marker GPNMB^29,30^ in the inflamed spinal cord (Fig. 5p).

**Extended Data Fig. 8:**
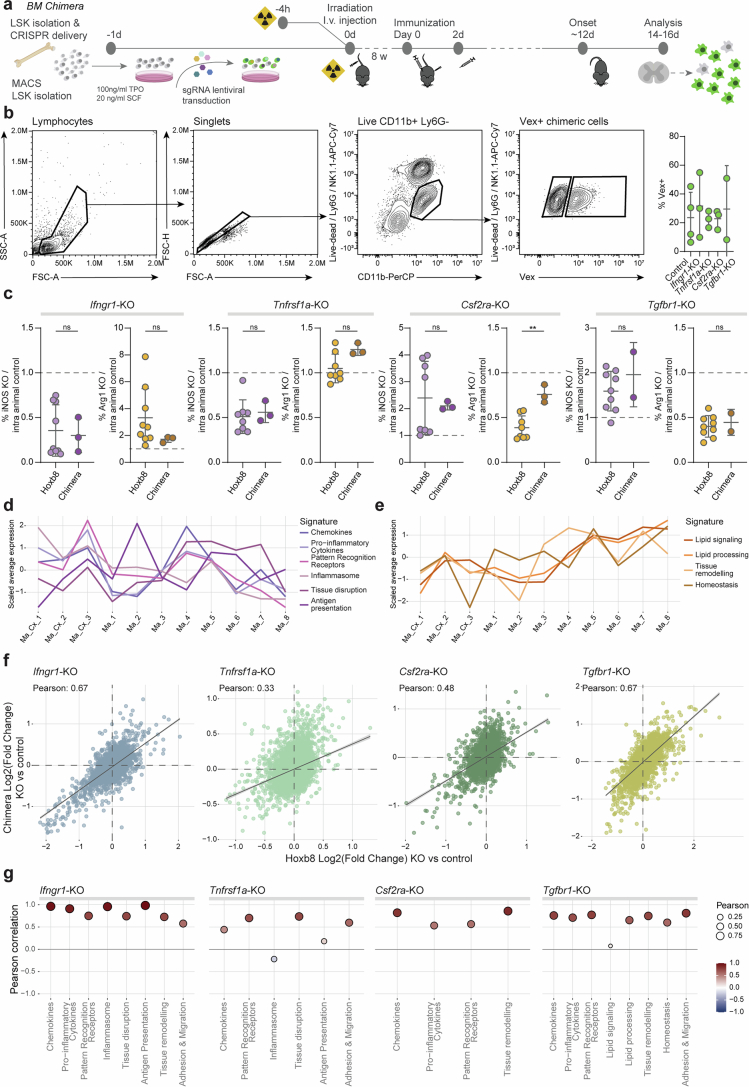
Cytokine receptor KOs in bone-marrow chimera-derived macrophages recapitulate the KO phenotypes of Hoxb8FL-derived macrophages. **a**, Scheme of the experimental approach to perform CRISPR editing of myeloid cells in bone marrow chimeras. **b**, Gating strategy for the sorting of CRISPR-edited Vex+ chimeric cells (left) and quantification of total CRISPR-edited cells amongst the monocyte/macrophages in the spinal cord at peak of EAE. *N* = 4 control, 3 *Ifngr1*-KO, *Tnfrsf1a*-KO and *Csf2ra*-KO and 2 *Tgfbr1*-KO animals. **c**, Comparison of the iNOS+ and Arg1+ polarization phenotype in flow cytometry between single-gene KOs in Hoxb8FL-derived and the chimera-derived macrophages. The polarization is expressed as polarization in the KO normalized to the intra-animal control. The data derived from Hoxb8FL-cells are the same as shown in Fig. 2, for chimera *n* = 3 *Ifngr1*-KO, *Tnfrsf1a*-KO and *Csf2ra*-KO and 2 *Tgfbr1*-KO animals. **d, e**, Expression pattern of curated transcriptional signatures reflecting distinct properties of macrophages presumably contributing to tissue destruction (**c**) or lesion resolution (**d**) across macrophage clusters derived from bone-marrow chimeras. **f**, Correlation of the log2(Fold Change) phenotype of all genes between the chimera-derived and the Hoxb8FL-derived macrophage KOs compared to controls. **g**, Pearson correlation of the individual signatures’ gene regulation in the KO macrophages compared to control between Hoxb8FL-derived and chimera-derived macrophage phenotypes. Only significant signatures (as shown in Figs. 4 and 5) per KO are shown. (**b, c**), two-tailed unpaired t-test or Wilcoxon test; (**e, f**), Pearson correlation. *P* < 0.0001; ns p-value > 0.05, * p-value < 0.05, ** p-value < 0.01, *** p-value < 0.001, **** p-value < 0.0001; figures show mean ± sd. Source data

Taken together, these analyses demonstrate how combining CRISPR gene editing with single transcriptomics and in situ and in vivo imaging can be applied to comprehensively reveal the regulation of macrophage states, their differentiation and molecular specification in vivo. In this context, it is interesting to note that similar regulatory patterns with regard to both the temporal evolution of gene signatures and their regulation by individual cytokine receptor KOs were observed between Hoxb8FL-derived and bone-marrow chimeric macrophages (Extended Data Fig. 8), supporting the view that Hoxb8FL-derived myeloid cells are a valid tool to dissect the molecular regulation of macrophages in vivo.

### Gene-set enrichment analysis reveals TGFβ as a regulator of CNS tissue invasion

**Fig. 6:**
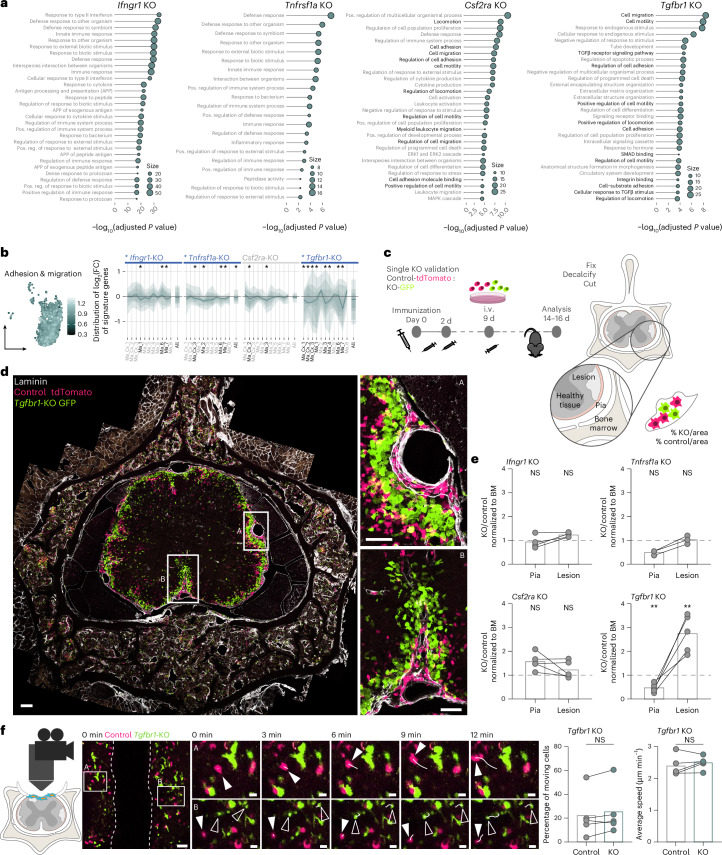
TGFβ signaling instructs macrophage localization but not their mobility within spinal EAE lesions. **a**, Unbiased gene-set analysis of GO biological process gene sets downregulated by cytokine receptor KOs (see Methods for details on the calculation of the KO-specific differentially regulated genes). GO terms related to cell migration or adhesion are highlighted in bold. For statistical analysis, gprofiler analysis was used (Methods). **b**, Left, expression of the transcriptional signature ‘Adhesion & Migration’ in the Hoxb8FL-derived control cells; right, phenotype of the KOs in control Hoxb8FL-derived cells for this signature across clusters (Ma_Cx_1 to Ma_8) and overall (All) compared to the control cells. The shading/lines represent the distribution of log_2_(fold change) of the KO compared to the control for all the genes that make up the signature, with the thick line being the median (quantile 50) log_2_(fold change), and the thinner overlaid shadows representing the lines of the adjacent quantiles: 40th and 60th quantiles of the log_2_(fold change) delimit the darkest shadow flanking the median line, in steps of 10 until the 10th and 90th quantiles delimiting the lightest shadow at the edges. For statistical analysis, GSEA was used (Methods). **c**, Scheme of the experimental approach to study the migration phenotype of the *Tgfbr1*-KO myeloid cells. A mix of tdTomato^+^ control cells and eGFP^+^ KO Hoxb8FL cells was transferred into an immunized mouse before disease onset; at the peak of disease the animal was perfused, and then the spinal column was decalcified and cut coronally. **d**, Representative confocal microscopy image of a cross-section of the whole spinal column of an EAE animal co-transferred with control (red) and *Tgfbr1*-KO (green) cells. Higher-magnification image on the right shows myeloid cell infiltration in the pia delimited by laminin staining, and in adjacent parenchymal lesions. Scale bars, 50 μm. **e**, Quantification of the distribution of the Hoxb8FL-derived cytokine receptor KO myeloid cells relative to the control cells in the same animal across compartments. *N* = 4 animals for *Ifngr1*-KO, *n* = 3 animals for *Tnfrsf1a*-KO and *n* = 5 animals for *Csf2ra*-KO and *Tgfbr1*-KO. **f**, Scheme of the experiment for tracking the movement of Hoxb8FL-derived myeloid cell in neuroinflammatory lesions (left); time-lapse images showing cell movement along the midline vein in the dorsal spinal cord at peak of EAE of control-edited and *Tgfbr1*-KO Hoxb8FL-derived myeloid cells (middle); and quantification of movement parameters (right). Filled white arrowheads indicate control cells, outlined white arrowheads indicate *Tgfbr1*-KO cells, and lines denote cell tracks. Scale bars, 50 μm (overview image) and 10 μm (insets). Pathways shown in **a** include only GO_BP and GO_MF terms, with an adjusted *P* value < 0.01, an intersection size ≥ 5 and term size < 2,000; **b**, KO names marked by asterisks at the top of the plots and black ‘All’ labels indicate that the phenotype of the signature for that KO versus control in the global Hoxb8FL-derived cells is significant. Blue indicates downregulated in the KO compared to the control. Asterisks and black cluster name labels indicate the phenotype is significant for that cluster for the Hoxb8FL-derived KO versus the control. Significance was determined with the GSEA pathway analysis algorithm for a NOM *P* value < 0.05 or an FDR *q* value < 0.25 and absolute NES > 1.5 (Methods); **e**, Two-tailed paired *t*-test or Wilcoxon test for KO versus control for pia and for parenchyma separately; data are shown as a ratio for convenience. **f**, Two-tailed paired *t*-test or Wilcoxon test. NS, *P* value > 0.05; *P value < 0.05, **P value < 0.01, ***P value < 0.001, ****P value < 0.0001; bars show the mean. Source data

**Extended Data Fig. 9:**
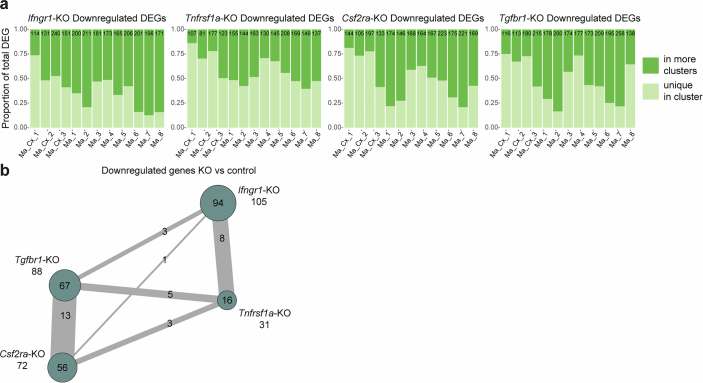
Cytokine-induced genes are consistent across macrophage clusters. **a**, Proportion of downregulated genes in the KO vs the control that are unique to the cluster or shared between more than one cluster. Numbers at the top of each column indicate the total number of downregulated DEGs per cluster. Downregulated DEGs per KO are defined as genes significantly downregulated (adjusted p-value < 0.05 and log2(Fold Change) < 2 x standard deviation of the log2(Fold Change) distribution of the comparison as analyzed by Seurat FindMarkers) for the same cluster in either the Hoxb8FL-derived KO cells compared to the Hoxb8FL control or the chimeric KO cells compared to the chimeric control. **b**, Downregulated gene overlap across KOs. See Methods for definition of downregulated genes in the KO. Numbers below the KO name indicate total genes downregulated in that KO, numbers inside of the circles indicate genes uniquely downregulated in that KO, and numbers on the edges indicate genes regulated by the two connected KOs. Source data

To reveal additional regulatory aspects of cytokine signaling beyond the curated signatures, we performed an unbiased analysis of the differentially regulated genes in the cytokine receptor-deficient compared to the control-edited macrophage populations (Methods). We observed that most of the regulated genes in any of the cytokine receptor deletion experiments were similarly altered in multiple macrophage clusters, suggesting that the cytokine regulators induce common transcriptional responses across macrophage subpopulations (Extended Data Fig. 9a). Notably, we again saw a sizable overlap in the genes differentially regulated by TGFβ and GM-CSF, while IFNγ induced expression of a distinct gene set (Extended Data Fig. 9b). An unbiased biological processes Gene Ontology (GO) term analysis of the differentially regulated genes revealed that GO terms related to cell adhesion and migration were among the top biological processes regulated by CSF2RA and TGFBR1 (Fig. 6a). Indeed, a curated signature including genes related to macrophage adhesion and migration identified TGFBR1 as a putative regulator of these functions (Fig. 6b). To further investigate how the migration pattern of myeloid cells in neuroinflammation might be modulated by the essential cytokine regulators, we co-transferred Hoxb8FL-derived myeloid cells deficient for IFNGR1, TNFR1, CSF2RA or TGFBR1 with control-edited cells and characterized the distribution of transferred cells within neuroinflammatory lesions. Here we observed a striking difference in the distribution of TGFBR1-deficient macrophages between leptomeninges and CNS parenchyma with TGFBR1-deficient cells being basically absent from the pia and enriched in the CNS lesion parenchyma. This altered distribution appeared to be specific for TGFBR1-deficient macrophages as it was not observed for any of the other cytokine receptor-deficient cells (Fig. 6c–e). To assess whether the differential distribution of TGFBR1-deficient myeloid cells in the inflamed CNS results from an altered mobility of these cells, we used in vivo microscopy to track the movement pattern of control-edited and TGFBR1-deficient Hoxb8FL-derived myeloid cells in EAE lesions in the superficial dorsal spinal cord. Our results showed that neither the proportion of cells that move nor the speed at which they move are affected by the absence of TGFBR1 (Fig. 6f). Taken together, these experiments thus reveal that TGFBR1 signaling instructs the distribution of macrophages in the inflamed CNS without affecting their ability to move within neuroinflammatory lesions.

### Perturbation-derived neuroinflammatory cytokine signatures reveal regulation of myeloid cells across CNS compartments and lesion types in MS

**Fig. 7:**
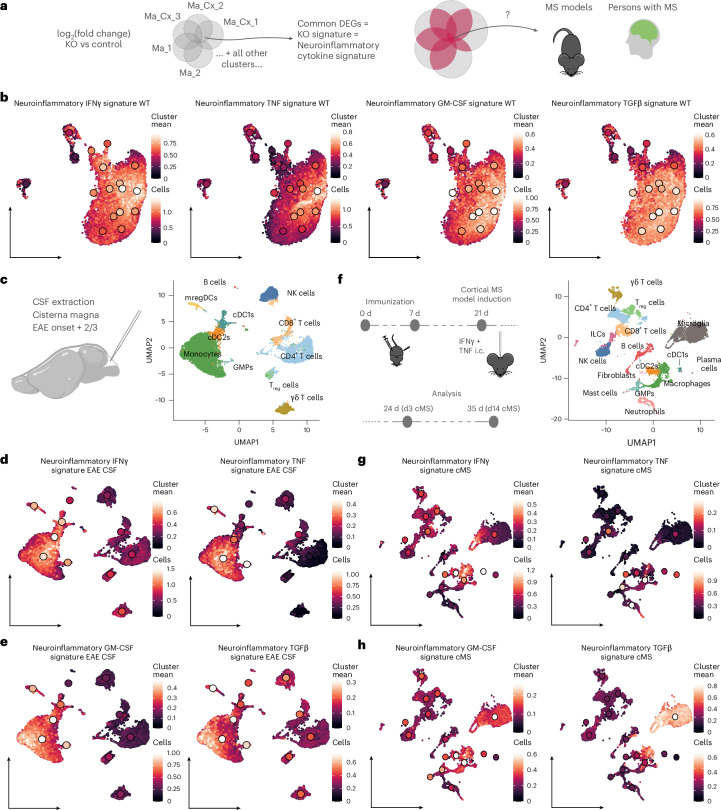
Neuroinflammatory cytokine signatures connect myeloid cell regulation across MS models. **a**, Scheme illustrating how we derived neuroinflammatory cytokine signatures. **b**, Expression of the averaged neuroinflammatory cytokine signatures in the WT EAE UMAP. **c**, Scheme of the CSF extraction procedure from the cisterna magna (left) and UMAP of the immune cells present in CSF of EAE animals at the peak of disease (right). **d**,**e**, Expression of the averaged neuroinflammatory cytokine signatures in the CSF EAE UMAP. **f**, Scheme of the induction of the cortical MS (cMS) disease model (left) and UMAP of the immune cells present in the cortex of cMS animals at the peak of inflammation (right). i.c., intracortically. **g**,**h**, Expression of the averaged neuroinflammatory cytokine signatures in the cMS UMAP. DEGs, differentially expressed genes.

**Extended Data Fig. 10:**
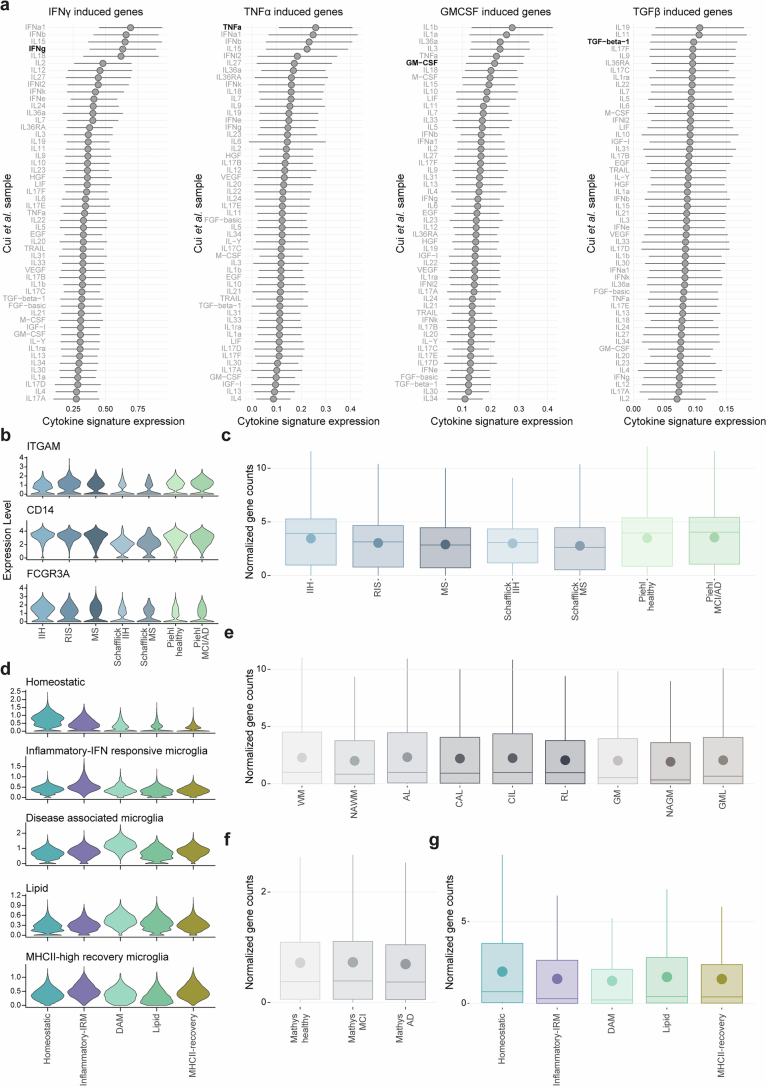
Characterization of neuroinflammatory cytokine signatures and myeloid cells from human CSF and tissue samples. **a**, Neuroinflammatory cytokine signature expression across different cytokine injected samples based on Cui et al. (ref. ^31^). Dots represent the mean, lines the standard deviation **b**, Expression of myeloid markers in CSF monocyte/macrophages **c**, Distribution of the gene counts of all detected genes across CSF datasets. **d**, Characterization of human microglial clusters. **e**, Distribution of the gene counts of all detected genes across Macnair et al. (ref. ^35^) lesion types. **f**, Distribution of the gene counts of all detected genes across Mathys et al. (ref. ^36^) pathology. **g**, Distribution of the gene counts of all detected genes across Macnair et al. (ref. ^35^) microglial clusters. **b, e-g**, Dots represent the mean. Boxplot line shows median, box Q1 and Q3, and whiskers the interquantile range x 1.5. Source data

We next examined whether insights from experimental neuroinflammatory lesions could reveal cytokine-driven regulation of myeloid cells in individuals with MS. Using genes induced by a given cytokine across mouse myeloid populations, we defined ‘neuroinflammatory cytokine signatures’ representing cytokine-regulated programs in inflammatory contexts (Fig. 7a and Methods). These signatures only partially overlapped with those from homeostatic conditions^31^ (Extended Data Fig. 10a) and were prominently expressed in myeloid clusters within MOG EAE white matter lesions, particularly in populations dependent on the respective cytokine (Figs. 3 and 7b). We further analyzed immune cells from the cerebrospinal fluid (CSF) and gray matter of mice with MOG EAE and cortical MS-like pathology. In both compartments, IFNγ, TNF, GM-CSF and TGFβ signatures were strongly enriched in myeloid but not adaptive immune cells, with high expression of the signatures induced by IFNγ, GM-CSF and TGFβ also detected in microglia (Fig. 7c–h). These findings indicate that neuroinflammatory cytokine signatures are conserved across myeloid populations and CNS compartments, including gray matter and CSF.

**Fig. 8:**
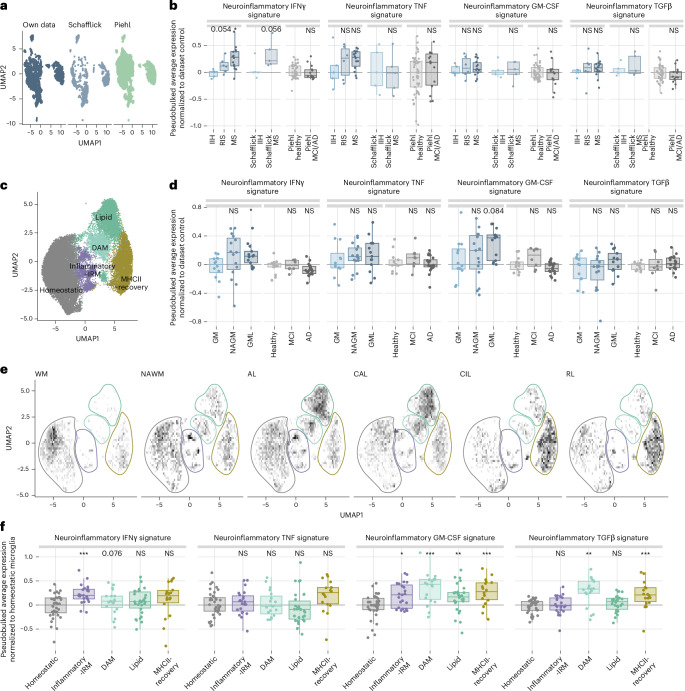
Neuroinflammatory cytokine signatures reveal myeloid cell regulation in MS across CNS compartments. **a**, UMAP of human CSF monocyte/macrophages across datasets after integration. **b**, Pseudobulked average expression per individual of the neuroinflammatory cytokine signatures across human MS CSF datasets and disease stages, and a control CSF dataset from MCI/AD. *N* = 5 idiopathic intracranial hypertension (IIH), 6 radiologically isolated syndrome (RIS) and 14 individuals with MS, *n* = 5 individuals with IIH and 5 individuals with MS from ref. ^32^, *n* = 45 healthy individuals and 14 individuals with MCI/AD from ref. ^33^. **c**, UMAP of human microglia from ref. ^35^. IRM, interferon-responsive microglia; DAM, disease-associated microglia. **d**, Pseudobulked average expression per individual of the neuroinflammatory cytokine signatures in microglia derived from gray matter of control individuals or people with MS or MCI/AD. *N* = 12 samples for control gray matter (GM), 15 for normal-appearing gray matter (NAGM) in MS and 13 for gray matter lesion (GML) in MS, *n* = 13 healthy individuals, 8 individuals with MCI and 20 individuals with AD from ref. ^36^. **e**, Density distribution of microglial clusters across white matter lesion types in human MS. WM, control white matter; NAWM, normal-appearing white matter; AL, active lesion; CAL, chronic active lesion; CIL, chronic inactive lesion; RL, remyelinating lesion. **f**, Pseudobulked average expression per individual of the neuroinflammatory cytokine signatures across microglial clusters in the white matter. Respectively, *n* = 36, 23, 19, 30 and 18 samples in homeostatic to MHC class II-recovery clusters. **b**, One-way ANOVA, IFNγ *F* = 4.9165, *P* = 0.0171, TNF *F* = 0.7544, *P* = 0.4821, GM-CSF *F* = 0.2334, *P* = 0.7938, TGFβ *F* = 0.727, *P* = 0.5088, followed by two-tailed *t*-tests or Wilcoxon tests comparing RIS/MS/MCI-AD to their dataset control (IIH or healthy); **d**, One-way ANOVA IFNγ *F* = 2.0908, *P* = 0.1379, TNF *F* = 0.2422, *P* = 0.7861, GM-CSF *F* = 2.0325, *P* = 0.1454, TGFβ *F* = 1.5998, *P* = 0.2156, followed by two-tailed *t*-tests NAGM and GML to healthy GM for ref. ^35^ MS data; IFNγ *F* = 1.803, *P* = 0.168, TNF *F* = 1.1469, *P* = 0.3284, GM-CSF *F* = 6.0476, *P* = 0.0052, TGFβ *F* = 0.7814, *P* = 0.465, followed by two-tailed *t*-tests comparing MCI/AD to healthy for ref. ^36^ MCI/AD data; **f**, one-way ANOVA TNF *F* = 2.3413, *P* = 0.0588, Kruskal–Wallis test IFNγ *P* = 0.0039, GM-CSF *P* < 0.0001, TGFβ *P* < 0.0001, followed by two-tailed *t*-tests or Wilcoxon tests comparing all clusters to the homeostatic cluster, adjusting the *P* values by the FDR method; In the box plots, the line shows median, the box extends from Q1 to Q3, and the whiskers extend to the smallest and largest values within 1.5 times the interquartile range from Q1 and Q3. NS, *P* value > 0.1, *P* value shown if 0.05 < *P* < 0.1, *P value < 0.05, **P value < 0.01, ***P value < 0.001, ****P value < 0.0001. Source data

We next investigated whether neuroinflammatory gene signatures could reveal cytokine actions on myeloid cells in individuals with MS. To this end, we analyzed induction of these signatures in CSF monocytes from people with multiple sclerosis. A published scRNA-seq dataset^32^ was integrated with our own dataset comprising untreated individuals early in the course of MS (Methods), together with a control set from individuals with mild cognitive impairment (MCI) and Alzheimer’s disease (AD^33^; Fig. 8a and Extended Data Fig. 10b,c). Pseudobulk analysis across monocyte populations revealed that CSF monocytes from people with multiple sclerosis—unlike those from MCI/AD controls—showed clear induction of the IFNγ signature (Fig. 8b). This is consistent with the reported induction of IFNγ-dependent genes in neurons as previously observed, for example, in the cortex of people with multiple sclerosis^34^.

To investigate cytokine activity in MS lesions, we analyzed a large single-nucleus RNA-seq dataset from postmortem brain samples of people with multiple sclerosis^35^. Tissue myeloid cells, predominantly microglia, were grouped into five clusters based on established molecular markers (Fig. 8c and Extended Data Fig. 10d). We then examined the presence of neuroinflammatory cytokine signatures in microglia from gray matter lesions and normal-appearing gray matter and compared them with microglia from MCI/AD donors^36^. Pseudobulk analysis per individual revealed induction of IFNγ and GM-CSF signatures in MS gray matter—most prominently within lesions—whereas no such induction was observed in microglia from MCI/AD samples (Fig. 8d).

These results suggest that essential cytokine regulators are active in the MS brain, prompting us to examine whether distinct microglial subsets are differentially influenced by IFNγ, TNF, GM-CSF and TGFβ. In MS white matter lesions^35^, microglial cluster frequencies varied by pathology stage: homeostatic microglia dominated healthy tissue, whereas inflammatory/interferon-responsive (IRM), disease-associated (DAM), lipid-processing and MHC class II-high microglia were enriched in MS lesions (Fig. 8e). IRM cells were abundant in active lesions and surrounding white matter, DAM and lipid-processing microglia clustered in active and chronic active lesions, while MHC class II-high cells appeared in inactive and remyelinating areas, indicating a recovery-associated phenotype^37^. Differential expression of cytokine signatures (relative to homeostatic microglia) revealed strong induction of an IFNγ signature in IRM, broad GM-CSF activation across lesion-associated populations, absent enrichment of a TNF gene signature and robust induction of a TGFβ signature in DAM and MHC class II-high microglia (Fig. 8f).

Taken together, these observations highlight that the neuroinflammatory cytokine signatures we delineated and validated in MS models are selectively induced in myeloid cells in neuroinflammatory but not in neurodegenerative conditions and can be used to uncover the differential regulation of myeloid cell states in MS across disease locations and stages.

## Discussion

We developed a CRISPR-based screening platform enabling systematic dissection of molecular factors underlying macrophage state regulation in inflamed tissues. Compared to classical conditional gene deletion or bone-marrow chimera approaches^38^, our method expedites experimental timelines, reduces animal use and ensures uniform sgRNA distribution through consistent myeloid cell expansion, facilitating large-scale analysis of over 100 genes in a single screen. Applying this strategy to cytokine receptor screening identified IFNγ, TNF, GM-CSF and TGFβ as key in vivo drivers of macrophage polarization, whereas IL-4, IL-10 and IL-13, which modulate polarization in vitro^21,39^, do not direct macrophage polarization in vivo at least in the active MS model at the studied time point. Using naive C57BL/6 recipients and in vitro-edited precursors enhances experimental flexibility, enabling integration with biosensor technologies and avoiding confounds from irradiation or genetic drift^40–42^. As Hoxb8FL-derived myeloid populations were detected in all analyzed tissues, this methodology is broadly applicable for interrogating myeloid cell regulation across diverse tissues and disease settings.

Successful application of this approach relies on an understanding of its inherent limitations. The method targets monocyte-derived myeloid cells, as microglia and most CNS-resident macrophages largely persist independently of blood-derived monocytes^43^. It is optimally suited for probing acute, cell-autonomous phenotypes, since Hoxb8FL-derived cells replace only ∼15% of endogenous populations, minimizing capacity to affect overall tissue pathology or disease trajectory. Nevertheless, this framework enables direct analysis of myeloid cell phenotypes within largely unaltered lesion contexts, isolating cell-intrinsic signaling pathways. The efficiency of large-scale CRISPR screens remains contingent on the yield of genetically modified cells from tissue; given the short lifespan of monocyte-derived cells, rapid immune infiltration models are preferable. Additionally, our approach relies on a finite set of sortable phenotypes—here, iNOS^+^ for lesion-promoting and Arg1^+^ for lesion-resolving myeloid cells, reflecting established roles in MS models and MS tissue linking iNOS^+^ polarization to active lesion formation, with subsequent transition to Arg1^+^ during recovery in MS models^8,15,25,44^. While this binary scheme encapsulates extremes, macrophage phenotypes likely span a broader continuum influenced by disease context, cell development and analytical methodology^13,14,45^. Importantly, our screening strategy can be extended to alternative markers, and cytokine-based regulation provides a foundation for dissecting additional regulatory pathways influencing myeloid states.

To delineate cytokine-induced macrophage states in neuroinflammation with single-cell resolution, we implemented Perturb-seq using scRNA-seq for CRISPR perturbation readout^46,47^. To validate the utility of the Hoxb8FL transfer approach for single-cell analyses, we performed parallel Perturb-seq in bone-marrow chimeras optimized for CRISPR editing of myeloid cells. Both methods demonstrated that cytokine receptor deletion induces similar shifts in macrophage subpopulation composition and transcriptional profiles (Fig. 3 and Extended Data Figs. 5–8), confirming the broad utility of CRISPR-edited progenitors for comprehensive, unbiased analysis of macrophage state regulation.

A key insight from our Perturb-seq analysis is that macrophage polarization is chiefly governed by the balance of IFNγ and TGFβ signaling. Both pathways not only promote distinct polarization states but also reciprocally inhibit the alternative phenotype, a phenomenon consistent across MS models. These findings implicate IFNγ as a central mediator of pro-inflammatory signatures, tissue damage and oxidative burst in myeloid cells, aligning with previous studies in MS models and people with MS^48–50^. Nonetheless, IFNγ actions are highly context dependent, potentially varying with EAE model, lesion stage, cofactors and responding cell type^48,51–53^.

Similarly to IFNγ, TNF signaling promoted pro-inflammatory, tissue-damaging macrophage signatures, corroborating studies where TNF blockade improved outcomes in MS models^54,55^ and TNFR1-deficient mice were protected from EAE^56^. However, neuroinflammatory TNF signatures were not prominently detected in lesion-associated microglia in MS. Clinically, anti-TNF therapies, effective in other autoimmune diseases^57,58^, have exacerbated MS pathology, a dichotomy partially explained by an MS-risk single nucleotide polymorphism (rs1800693) in *TNFRSF1A* that yields a soluble TNFR1 antagonizing TNF^59^. These converging findings suggest that TNF’s protective effects in MS may be mediated through non-macrophage populations, such as its known support for myelin repair via TNFR2 on oligodendrocyte precursor cells^60^.

Our CRISPR screen revealed that both GM-CSF and TGFβ are essential for Arg1^+^ macrophage polarization in EAE. This was unexpected given GM-CSF’s established pro-inflammatory role in neuroinflammation: mice lacking CSF2R in monocytes are resistant to EAE due to impaired induction of inflammatory and oxidative stress-related genes^23,61^. Perturb-seq confirmed these pro-inflammatory effects yet uncovered an unanticipated contribution of GM-CSF to tissue remodeling, resembling TGFβ activity. The considerable overlap between GM-CSF-regulated and TGFβ-regulated gene sets (Extended Data Fig. 9c) mirrors their reported synergy in promoting scar-associated macrophages^62^, highlighting the complexity of GM-CSF as both a pro-inflammatory and reparative cytokine—an important consideration given its candidacy as a therapeutic target in MS and other inflammatory conditions^63–65^.

Consistent with earlier studies, TGFβ induced transcriptional programs of homeostasis, debris clearance and tissue repair. Loss of TGFBR1 impaired lipid efflux, resulting in foamy macrophages expressing GPNMB and accumulating lipids and cholesterol crystals, a phenotype linked to progressive MS pathology^66–68^. An intriguing aspect of this regulation is that it appears to be distinct in monocyte-derived macrophages^69^ compared to microglia in which TGFβ was shown to limit effective lipid clearance at least in a model of toxic myelin damage^70^. Beyond its established homeostatic functions, we identified a critical role for TGFβ signaling in regulating myeloid cell infiltration during neuroinflammation. Although TGFβ has long been associated with cell motility and epithelial–mesenchymal transition in cancer^71–73^, our data indicate a distinct mechanism in the CNS: TGFβ does not affect macrophage movement within lesions but instead governs their progression from meninges into the parenchyma, an effect that might well contribute to the worsening of CNS inflammation observed in the absence of TGFβ signaling in myeloid cells^74^.

Single-cell analysis of cytokine actions revealed broadly consistent effects of individual cytokines on myeloid cell specification across macrophage clusters, indicating that response intensity rather than direction depends on the differentiation state of the cell. However, cytokine-induced gene programs were partially shaped by the local inflammatory milieu, as transcriptional responses in neuroinflammatory lesions only partly overlapped with those observed after cytokine stimulation in lymph nodes^31^ (Extended Data Fig. 10a). The uniform cytokine responsiveness within lesions suggests that core signaling mechanisms are conserved across myeloid cell types and possibly species, providing a framework for cross-species comparison of myeloid activation in disease. From our Perturb-seq data, we defined neuroinflammatory cytokine signatures for IFNγ, TNF, GM-CSF and TGFβ, and confirmed their enrichment across murine myeloid populations not only in inflamed white matter, but also in gray matter lesions and the CSF compartment.

Analysis of human CSF monocytes revealed selective upregulation of IFNγ-responsive genes in people with MS, paralleling IFNγ presence in CSF^75^ and its induced transcriptional signatures in MS neurons^34^ and gray matter microglia as we observe here. In white matter, IFNγ signatures predominated in inflammatory microglia, while GM-CSF—but not TNF—signatures extended across lesion-associated clusters, implicating GM-CSF in sustained microglial activation^76,77^. In contrast, TGFβ signatures were enriched in MHC class II^+^ microglia from inactive and remyelinating lesions. This indicates that TGFβ not only maintains the homeostatic phenotype of microglia^78^, but also provides an important signal that drives a distinct microglial phenotype associated with tissue recovery^37^. Collectively, our findings demonstrate that neuroinflammatory cytokine signatures can delineate which cytokines influence specific myeloid cell populations across CNS compartments and neurological conditions. This analytical approach is adaptable to diverse mediators and transcriptomic datasets, offering a powerful tool for uncovering molecular drivers of myeloid cell heterogeneity in humans.

In conclusion, we established a rapid, scalable and versatile in vivo CRISPR screening approach to dissect the signals driving myeloid cell states across tissue compartments and models, providing insights into myeloid cell regulation in human disease, an important step toward the rational design of therapies targeting innate immune cells.

## Methods

### Compliance with ethical regulations

Animal care and experiments were carried out in accordance with the regulations of the applicable animal welfare acts and using protocols approved by the responsible regulatory authority (Regierung von Oberbayern, commission number 15). For human data, the collection of samples was approved by the local ethics committees of the LMU, Munich (ethical vote: 163-16, equivalent of the Institute Review Board (IRB) number for the ethics committee). Written informed consent was obtained from all individuals according to the Declaration of Helsinki.

### Contact for reagent and resource sharing

Further information and requests for resources and reagents should be directed to and will be fulfilled by the corresponding authors, M.K. and A.K. Plasmids generated in this study are available upon reasonable request.

### Experimental model and animal details

#### Plasmids

All primer sequences are listed in Supplementary Table 1.

The MSCV-v2-U6-(BbsI)-Pgk-Puro-T2A-eGFP (with v2 improved scaffold) vector was generated as follows: first, the pU6-Pgk-Puro-T2A construct was PCR amplified from pKLV2-U6gRNA5(BbsI)-PGKpuro2ABFP-W (Addgene), and the eGFP construct was PCR amplified from pMSCV-Cas9-eGFP (in house); then, the PCR product was assembled into the SalI (NEB) + XhoI (NEB) digested pMSCV-neo (Takara Clontech) vector using the Gibson Assembly Master Mix (NEB). The pMSCV-v2-U6-(BbsI)-Pgk-Puro-T2A-BFP (with v2 improved scaffold) vector was generated as follows: the pU6-Pgk-Puro-T2A-BFP construct was PCR amplified with overhangs for SalI + XhoI from pKLV2-U6gRNA5(BbsI)-PGKpuro2ABFP-W, then the PCR product was digested and ligated into SalI + XhoI digested pMSCV-neo vector with Quick Ligase (NEB). The pMSCV-v2-U6-(sgNon-Targeted)-Pgk-Puro-T2A-Tdtomato (with v2 improved scaffold) vector was generated as follows: first, the pU6-Pgk-Puro-T2A construct was PCR amplified from pKLV2-U6-(sgNon-Targeted)-PGKpuro2ABFP-W, and the Tdtomato construct was PCR amplified from pAAV-CAG-tdTomato (Addgene); then, the PCR product was assembled into the SalI + XhoI digested pMSCV-neo vector using the Gibson Assembly Master Mix. The pXPR_053-hPgk-VEX vector was obtained from Addgene^68^. The pMSCV-v2-U6-(BbsI)-Pgk-Grx1-roGFP2 vector was generated as follows: the pLPCX cyto Grx1-roGFP2 (Addgene) and pMSCV-v2-U6-(BbsI)-Pgk-Puro-T2A-BFP vectors were digested with BglII + ClaI and the Grx1-roGFP2 and pMSCV-v2-U6-(BbsI)-Pgk constructs were ligated with Quick Ligase. The pMSCV-v2-U6-(BbsI)-Pgk-mTFP1 vector was generated as follows: the mTFP1 construct was PCR amplified from pLJM1-FIRE-pHLy (Addgene), then the PCR product was assembled into the NotI + XhoI digested pKLV2-U6-(sgRNA)-PGKpuro2ABFP-W vector using the Gibson Assembly Master Mix.

#### Animals

C57BL/6 mice were purchased from Charles River or Janvier and bred in the Core Facility for Animal Models of the Biomedical Center, LMU. R26-Cas9-eGFP animals were ordered from The Jackson Laboratory (024858) and backcrossed onto a C57BL/6 background several times. BiozziABH mice, F1 C57BL/6J mice crossed with BiozziABH mice, and 2D2 mice were bred in house. Animal care and experiments were carried out in accordance with the regulations of the applicable animal welfare acts and using protocols approved by the responsible regulatory authority (Regierung von Oberbayern, commission number 15). All animals had free access to food and water. Animals were kept at a room temperature of 22 °C ± 2 °C and humidity of 55% ± 10%, with a 12-h/12-h light–dark cycle (6:30–18:30). Male and female mice between 2 and 8 months old at the start of the experiment were used.

#### Cell lines and primary cells

All cells were incubated in a humidified incubator at 37 °C and 5% CO_2_ in air. All media were supplemented with 10% FBS (Bio&SELL) and 1% penicillin–streptomycin (Thermo Fisher). HEK293T cells (American Type Culture Colllection) were kept in DMEM GlutaMAX (Thermo Fisher). Primary bone marrow cells and Hoxb8FL cells were kept in RPMI GlutaMAX (Thermo Fisher). Hoxb8FL cells were additionally supplemented with 0.1% 2-mercaptoethanol (Thermo Fisher), 1 μM β-estradiol (Sigma) and supernatant from a Flt3L-producing B16 melanoma cell line, to a final concentration of 35 ng ml^−1^. During macrophage differentiation the RPMI medium was additionally supplemented with M-CSF (10–20 ng ml^−1^; PeproTech). T cells were kept in RMPI 1640 (Sigma) and additionally supplemented with 10 mM HEPES, 2 mM L-glutamine, 1% non-essential amino acids, 1 mM sodium pyruvate and 50 µM β-mercaptoethanol. For freezing cells, a 10% dimethylsulfoxide (Sigma) and 90% FBS (Bio&SELL) mixture was used, and cryovials were kept in a freezing box for several days at −80 °C before transferring to liquid nitrogen. Cell lines were tested for the absence of mycoplasma. HEK239T cells were detached by using 0.05% Trypsin-EDTA (Thermo Fisher). Macrophages were detached with Accutase solution (Sigma).

#### Hoxb8FL generation

hBCL2-overexpressing Hoxb8FL Cas9 (Hoxb8FL) lines were generated as follows. Bone marrow cells were harvested from femurs and tibias of 6–10-week-old animals and cultured in RPMI supplemented with recombinant mouse IL-3 (5 ng ml^−1^), IL-6 (20 ng ml^−1^) and 1% cell culture supernatant from SCF-producing B16 melanoma cells. After 2 days, the cells were spin infected with MSCV-ERHBD-Hoxb8FL-carrying retrovirus. A day after spin-infection, the cells were cultured in Hoxb8FL medium until infected cells were enriched in the culture in the presence of β-estradiol^20^. The medium was replaced every 2–3 days. The hBCL2 overexpression improved the survival of these cells both during in vitro differentiation experiments and in vivo.

#### Individual sgRNA cloning

All sgRNA sequences are listed in Supplementary Table 1.

For individual sgRNA cloning, 20-nucleotide-long sgRNAs were picked from the GPP sgRNA designer tool from the Functional Genomics Consortium of The Broad Institute, Massachusetts, USA. sgRNA sequence and reverse complemented sequence were ordered as two separate oligonucleotides from Metabion with overhangs on the 5′ side of CAAC for forward and AAAC for reverse. The nucleotide ‘G’ was added as a first nucleotide to increase the efficiency of the gRNA expression by the hU6 promoter^79^. Complementary oligonucleotides with overhangs were phosphorylated and annealed in the presence of 10x T4 Ligation Buffer (NEB) and T4 PNK (NEB) by increasing the temperature to 95 °C and ramping down to 25 °C at 5 °C min^−1^. Annealed oligonucleotides were ligated into Bpil- or BsmbI-digested (Thermo Fisher) gRNA cargo plasmid by Quick Ligase (NEB) for 6 min at room temperature. Ligated plasmids were then transformed into Stellar competent cells (Takara Clontech) with heat shock at 42 °C for 55 s. Bacterial plates were incubated overnight, and single clones were picked and prepped with the Qiagen plasmid miniprep kit. The correct ligation product was confirmed with Sanger sequencing (Sequencing service, LMU Biozentrum) using the hU6 primer.

#### Oligonucleotide pool library cloning

sgRNAs were picked from the GPP sgRNA designer tool from Functional Genomics Consortium of The Broad Institute. All sgRNA sequences that were selected, and the library cloning primers, as well as protocols, are listed in Supplementary Table 1.

sgRNAs for the mouse Cytokine Receptor library were ordered from Integrated DNA Technologies as custom oPools (50 pmol per oligonucleotide) as a 79-mer with a sequence of 5’-GCAGATGGCTCTTTGTCCTAGACATCGAAGACAACACCGN_20_GTTTTAGTCTTCTCGTCGCC-3’, with N_20_ indicating the sgRNA sequence. Each gene was targeted with three different sgRNAs (*Stat6* with 4 sgRNAs), and 15 non-targeted (from here onward, control) control gRNAs were also included (343 oligonucleotides in total). The oligonucleotide pool was dissolved in Qiagen TE buffer to get a 100 μM stock concentration, and then the single-stranded oligonucleotides (100 ng) were PCR amplified for two cycles with Q5 High-Fidelity DNA Polymerase (NEB) using Oligo_Amp_F and Oligo_Amp_R primers to generate double-stranded DNAs. The PCR products were purified with the Nucleotide Removal Kit (Qiagen). Amplified double-stranded DNAs were digested with FastDigest BpiI (BbsI, Thermo Fisher) for 2 h at 37 °C in a total of two reactions, and then purified with the Nucleotide Removal Kit (Qiagen). Ligation was performed with a T4 DNA Ligase (NEB) using a 3 ng insert and 40 ng BpiI-digested MSCV-v2-U6-(BbsI)-Pgk-Puro-T2A-eGFP for 16 h at 16 °C per reaction in a total of two reactions. The ligated product was cleaned with a PCR Purification Kit (Qiagen), and the concentration was measured with Qubit 4 (Thermo Fisher). Ten nanograms of the ligated product was transformed into 50 μl of NEB Stable Competent cells in a total of eight reactions and incubated at 30 °C overnight. A library representation above 100× was confirmed by plating transformed competent cells in serial dilutions. The plasmid DNA was prepared with an Endofree Plasmid Maxi Kit (Qiagen).

#### Gene editing of Hoxb8FL cells

For viral transduction of Hoxb8FL cells, HEK293T cells were plated into a six-well plate 18–24 h before transfection. For the transfection per well of six-well plate, 1.5 µg pMSCV retroviral plasmid and 1.5 µg pCL-Eco packaging vector were added in 400 µl of RPMI medium without serum and antibiotics. Then 7.5 μl of the transfection reagent TransIT-LT1 were added into the mix, vortexed, and then incubated at room temperature for 30 min. After 30 min, the solution was added dropwise to HEK293T cells. After 4 h, the medium was replaced with Hoxb8FL medium. Retrovirus-containing supernatant was collected at 48–72 h after transfection. Hoxb8FL cells were spin infected with freshly harvested virus at 1,200*g* for 30 min at room temperature. For individual KO cell line generation, individual sgRNA viruses were produced and transduced. For the CRISPR screen experiments, the library oligonucleotide pool was used to generate a virus mix containing all the library sgRNAs, which was then transduced at a maximum multiplicity of infection of 0.3 or below (< 30% transduction efficiency) to prevent multiple integrations of sgRNAs into a single cell, and enough cell numbers were always kept ensuring a minimum 1,000× coverage (1,000 cells having the same sgRNA). For the Perturb-seq experiment, viruses for six individual non-targeted sgRNAs and two different sgRNAs per gene targeted were produced individually, and single KO or control cell lines were generated in parallel and mixed in equal proportions before expansion before i.v. injection. On the next day, puromycin (Thermo Fisher) was added at a final concentration of 5 µg ml^−1^ to select for transduced cells. After 4 days of puromycin selection, cells expressing fluorescent markers (for example, eGFP) were sorted to purity to ensure high expression of the fluorescent marker by using a FACS Aria III (BD) or FACS Fusion (BD) at the Flow Cytometry Core Facility of the Biomedical Center, LMU. The combination of positive selection of the infected cells by puromycin with sorting of the pure fluorescent population ensured all the Hoxb8FL transferred cells in the in vivo experiments were gene edited.

#### Tide assay

All single KO lines used in validation experiments were assessed for KO efficiency before the experiment with the TIDE assay. This assay works as follows: to assess the extent of genetic editing at DNA level for single sgRNAs, genomic DNA from sgRNA transduced (see above) Hoxb8FL cells was isolated with the DNeasy Blood and Tissue Kit (Qiagen), and the DNA region targeted by the sgRNA was amplified with Q5 High-Fidelity DNA Polymerase (NEB) and specific PCR primers for each different sgRNA. All TIDE primers are listed in Supplementary Table 1. The samples from KO and control cells were submitted to Sanger Sequencing with either the forward or the reverse PCR primer (Sequencing service, LMU Biozentrum). The Cas9-dependent generation of insertion–deletion repair errors and the subsequent KO efficiency (frame shifts that are not a multiple of 3 are considered to generate a protein KO) were assessed by the ICE v2 software tool^80^.

#### Intravenous transfer of Hoxb8FL cells

In vitro-expanded Hoxb8FL cells were washed twice with PBS to remove β-estradiol and cultured in RPMI medium (with 10 ng ml^−1^ M-CSF) for 2 days to initiate myeloid differentiation. On day 8–9 after active EAE induction, or at disease onset in T_H_17 adoptive transfer EAE (onset of clinical symptoms or >1 g of weight loss, or else the Hoxb8FL transfer stopped disease development), cells were washed twice with PBS and 10–15 × 10^6^ cells in 200 μl of PBS were injected i.v. into the tail vein of the mice.

For the intravital imaging experiments, Hoxb8FL cells were labeled before transfer with CellTrace Far Red (Thermo Fisher Scientific, C34564), CellTrace Yellow (Thermo Fisher Scientific, C34567) or CellTrace Violet (Thermo Fisher Scientific, C34571) for in vivo detection. After reconstituting the CellTrace according to the manufacturer’s instructions, cells were collected and the residual medium was washed once with PBS. Then, the cells were incubated 30 min at 37 °C in the dark in a 1:500 dilution of CellTrace solution in PBS at a concentration of 5 × 10^6^ cells per ml. Staining was stopped by adding five times the volume of cell medium and incubating for 5 min at 37 °C in the dark, followed by two PBS washes before injection.

#### Flow cytometry

##### Tissue isolation from EAE animals

Animals with EAE clinical signs were euthanized by isoflurane overdose. When CSF was collected, it was collected immediately after euthanasia from the cisterna magna. Briefly, the animal was head-fixed in a stereotactic frame, and the cisterna magna was exposed by keeping the head bent down at a 45° angle and removing skin and muscle from the neck. Then, a pulled glass pipette was used to carefully penetrate the cisterna magna and suction the CSF. Between 10 μl and 25 μl of CSF was collected per animal and immediately resuspended on FACS buffer (0.5% BSA, 1 mM EDTA, 25 mM HEPES on calcium and magnesium-free PBS) on ice. Blood samples were collected immediately after euthanasia or after CSF extraction by cardiac puncture, and the animal’s body was perfused with PBS-heparin before collecting the spleen, inguinal lymph nodes, bone marrow (femur and vertebra), spinal cord and brain cortex through microdissection. Cells from bone marrow were collected by flushing with ice-cold PBS, whereas cells from vertebrae were isolated after crushing with a pestle and mortar. Spleen, lymph nodes, spinal cord and brain tissues were homogenized with a glass dounce homogenizer (Wheaton) with a loose pestle, transferred into a PBS solution containing collagenase D (0.8 mg ml^−1^; Roche) and DNase I (10 ng ml^−1^; Roche) and incubated for 20 min at 37 °C with shaking (1,000 rpm) for dissociation of cells. For scRNA experiments, cells were incubated in digestion buffer for only 5 min to minimize changes to the transcriptome. Cells from spleen, lymph nodes (after digestion) and bone marrow were treated with ACK Lysing Buffer (Thermo Fisher) for 5 min, whereas cells from blood were incubated for 20 min on ice for red blood cell lysis. All cell suspensions were passed through a 100-μm-pore-diameter cell strainer (Corning). Cells from spinal cord and brain were isolated with Percoll (Sigma) gradient to remove the myelin. Cells were resuspended with 1 ml of 100% FBS and 9 ml of 33% Percoll (in PBS), and 1 ml of 10% FBS (in PBS) was added on the top slowly to form a layer. Samples were centrifuged without brakes at 800*g* for 15–30 min at 4 °C. The myelin layer was carefully sucked with a vacuum, and pelleted cells were washed with PBS to remove the Percoll solution.

##### CRISPR library experiments

For the cytokine library experiment, around ten animals were combined for one replicate to ensure sufficient cells (as, depending on the disease score, cell numbers found in the spinal cord vary). Before starting flow cytometry labeling, monocytes/macrophages from the spleen and bone marrow were enriched with MACS using anti-CD11b microbeads (Milteny) to reduce sorting time. All cells were blocked with TruStain fcX (anti-mouse CD16/CD32, BioLegend) and stained with Live/Dead Near-IR (Thermo Fisher) to exclude dead cells during blocking for 30 min at 4 °C. After washing with PBS, cells were incubated with antibodies recognizing extracellular markers for 30 min at 4 °C, with anti-CD11b-PerCP antibody to include monocytes/macrophages and anti-Ly6G-BV786 antibody to exclude granulocytes. Cells from the spinal cord were then fixed and permeabilized using Cytofix/Cytoperm kit (BD Biosciences) for 15 min at 4 °C, before intracellular labeling. Cells were incubated with anti-Arg1-APC and anti-iNOS-Pacific Blue antibodies for 30 min at 4 °C in Perm/Wash buffer solution. Hoxb8FL-derived cells were identified by their eGFP fluorophore expression. Populations of interest were isolated using a FACS Aria III (BD) or FACS Fusion (BD) at the Flow Cytometry Core Facility of the Biomedical Center. Enough cells were sorted for each population to ensure a library coverage of a minimum of 100× (100 cells containing each sgRNA). Genomic DNA from sorted cells was isolated by using the QIAamp DNA Micro Kit. Cells were lysed in the presence of Proteinase K at 56 °C overnight (modified from the manufacturer’s protocol) for more efficient de-crosslinking of fixed DNA before isolation of genomic DNA.

##### In vitro polarization

For in vitro macrophage polarization experiments, bone marrow-derived cells were cultured in RPMI medium with 10 ng ml^−1^ M-CSF for 5–7 days to allow differentiation. Differentiated cells were then re-seeded and the respective recombinant mouse cytokines were added at a concentration of 10–20 ng ml^−1^ as reported in the literature^81^ for 48 h before FACS: IL-4 (R&D Systems), TGFβ (BioLegend), GM-CSF (PeproTech), IFNγ (R&D Systems) or TNF (PeproTech) were used. Cells were detached with Accutase followed by two washing steps with PBS. Cells were fixed and permeabilized using the Cytofix/Cytoperm kit for 15 min at 4 °C before incubation with anti-Arg1 and anti-iNOS antibodies for 30 min at 4 °C in Perm/Wash buffer solution. Cells were analyzed with a Fortessa (BD) or a Cytoflex S (Beckman Coulter).

##### In vivo Hoxb8FL-derived cell characterization

For characterization experiments of Hoxb8FL-derived cells, nonspecific binding was blocked with TruStain fcX (anti-mouse CD16/32, BioLegend) and cells were stained with Live/Dead reagent (Thermo Fisher) to exclude dead cells, for 30 min at 4 °C. After washing with PBS, cells were incubated with antibodies recognizing extracellular markers for 30 min at 4 °C: anti-CD11b, anti-CD45, anti-Ly6G, anti-Ly6C, anti-MHC class II and anti-F4/80 were used. Cells then were incubated with anti-Arg1 and anti-iNOS antibodies for 30 min at 4 °C in Perm/Wash buffer solution. All antibodies—unless otherwise stated—were used at 1:100 dilution. Hoxb8FL-derived cells were identified by eGFP fluorophore expression, and eGFP-negative cells were assigned as the endogenous population. Cells then were analyzed using a FACS Aria III (BD) or FACS Fusion (BD) at the Flow Cytometry Core Facility of the Biomedical Center. Neutrophils were excluded from all analyses by expression of Ly6G.

For single KO validation experiments, before the transfer of Hoxb8FL cells into animals, Hoxb8FL cells expressing tdTomato and control sgRNA were mixed at a 1:1:1 ratio with Hoxb8FL cells expressing either BFP or eGFP and the gene targeting sgRNA to minimize the inter-animal experimental variation.

##### scRNA-seq experiments

For the 10x scRNA-seq experiments, before euthanasia, 3 μg per mouse of anti-CD45 antibody was injected i.v. in the tail vein to permit exclusion of cells derived from blood. Cells from 7 mice were pooled from a single EAE round for the WT EAE experiment; cells from 6 (round 1) and 12 (round 2) mice were pooled from two independent Hoxb8FL cell generation and EAE rounds for the Hoxb8FL-derived cell experiment; for the chimera experiment, three mice each with control, *Ifngr1*-KO, *Tnfrsf1a*-KO and *Csfr2ra*-KO BM chimera, and two mice with *Tgfbr1*-KO BM chimera were pooled; for CSF sequencing, 11 (round 1) and 12 mice (round 2) were pooled; for cEAE, 3 healthy, 3 d3 cEAE and 3 d14 cEAE mice were pooled. All cells were incubated with TruStain fcX (anti-mouse CD16/32, BioLegend) and Live/Dead Near-IR (Thermo Fisher) to exclude dead cells for 30 min at 4 °C. After washing with PBS, cells were incubated with anti-CD11b-PerCP antibody to include monocytes/macrophages and anti-Ly6G-BV786 antibody to exclude granulocytes, for 30 min at 4 °C. For the cEAE experiment, cells were additionally incubated with CD45-PE antibody to include all immune cells. Hoxb8FL-derived cells were identified by eGFP fluorophore expression, and eGFP-negative cells were assigned as the endogenous population. Chimeric cells were identified by Vex fluorophore expression. Populations of interest (WT, Hoxb8FL and chimeric EAE: CD11b^+^Ly6G^−^; CSF and cEAE CD45^+^Ly6G^−^) were sorted for purity using a FACS Aria III (BD) or FACS Fusion (BD) at the Flow Cytometry Core Facility of the Biomedical Center. Sorted cells were immediately prepared for 10x single-cell experiments according to the 10x protocol. The gating strategy to sort for Ly6G^−^ cells resulted in minimal numbers of neutrophils in the spinal EAE scRNA-seq experiments: 310 cells, 2% of total, in the WT dataset, 31 cells, 0.28% of total, in the Hoxb8FL-transfer dataset and 174 cells, 0.53% of total, in the bone-marrow chimera dataset.

### Statistical analysis and software

No statistical methods were used to predetermine sample sizes, but our sample sizes are similar to those reported in previous publications^8,82,83^. For statistical analyses and plotting, GraphPad Prism versions 7 and 9 (GraphPad Software) and R^84^ (version 4.0.0^+^) were used. For next-generation sequencing sample demultiplexing, Je-Demultiplex^85^ was used in Galaxy^86^. For analysis of CRISPR screens, cutadapt^87^, trimmomatic^88^, the MAGeCK software^89^, Galaxy^86^ and R^84^ were used. For analysis of bulk Galaxy^86^, RNA STAR^90^ (version 2.7.2b in Galaxy), HTSeq-count^91^ (version 1.0.0 in galaxy), DESeq2 (ref. ^92^; version 2.11.40.7 + galaxy1) and R^84^ were used. For image analysis, Fiji/ImageJ^93^ and Imaris (Oxford Instruments) were used. For scRNA-seq data, Seurat^94–98^ (versions 4+) and R^84^ were used. Figures were made using the aforementioned software and Adobe Illustrator 2025. Data are represented as the mean ± standard deviation unless otherwise stated; box plots show the median and quartiles 1 and 3 for the box limits, and whiskers extend to 1.5 times the interquartile range. Sample sizes are reported in the figure legends. All replicates are biological unless otherwise stated, measurements were not repeated, and all tests are two tailed unless otherwise stated. The choice to do a one-tail or two-tailed test depended on whether there were prior data suggesting the expected effect would be in a specific direction. Asterisks indicate *P* value > 0.05 (NS), *P value < 0.05, **P value < 0.01, ***P value < 0.001 and ****P value < 0.0001, unless otherwise specified. Samples were tested for a normal distribution using the Shapiro–Wilk normality test, and the statistical test used were chosen so the data met the assumptions of the test. Animals were randomly assigned to experiments or, when not possible, the conditions were appropriately blocked such that there were animals of the same condition in all cages. Due to the experimental design and animal laws, no blinding was possible during FACS experiments and Hoxb8FL characterization histology. The analysis of all histological data was done blinded. scRNA-seq exclusion criteria for low quality control are detailed in the Supplementary Methods. No other data were excluded. For statistical testing of the cell distribution in the lesion (Fig. 1b), an ordinary two-way ANOVA with the two-stage linear step-up procedure of Benjamini, Krieger and Yekutieli for multiple comparisons was used. For the Hoxb8FL transfer experiments (Figs. 1, 2, 5 and 6 and Extended Data Figs. 1, 2 and 7), multiple paired *t*-tests or Wilcoxon tests (when the sample was not normally distributed) with the two-stage linear step-up procedure of Benjamini, Krieger and Yekutieli for multiple comparisons with FDR (*q*) of 5% when needed were used. For comparison of EAE disease course in WT and Hoxb8FL transferred animals (Extended Data Fig. 1o), the Kolmogorov–Smirnov test was used. For the intravital imaging experiments (Figs. 4 and 5), for the proportion of cells in each ratio bin, an ordinary two-way ANOVA with a single pooled variance, for the interaction of genotype and bin was run; a Kolmogorov–Smirnov test was run for cumulative distributions, and a one-tailed unpaired (Fig. 4) or two-tailed paired (Fig. 5) *t*-test for the proportion of cells above Q3 of the ratio. For the histological quantifications, paired *t*-test or Wilcoxon tests were run. For the cholesterol efflux assay, unpaired *t*-tests were run. For the pHrodo-myelin phagocytosis assay, an ordinary two-way ANOVA with a single pooled variance for the genotype differences was run. For the single KO phenotype differences in polarization between Hoxb8FL and chimera, unpaired *t*-tests or Wilcoxon tests were run. For the KO/control distribution phenotype (Fig. 6), paired *t*-tests or Wilcoxon tests comparing KO and control were used, but data are plotted as the KO/control ratio for visualization purposes only. For the motility analysis, paired *t*-tests were run. For CRISPR screen results (Figs. 1 and 2 and Extended Data Figs. 2 and 3), the MAGeCK software was used as described above. Asterisks indicate *P* value and FDR < 0.05 and absolute log_2_(fold change) > 3 times the standard deviation of the noise distribution, as previously described. For comparison of in vitro bone marrow-derived macrophage polarization conditions (Extended Data Fig. 4), an ordinary one-way ANOVA with the two-stage linear step-up procedure of Benjamini, Krieger and Yekutieli for multiple comparisons was used. Bulk RNA-seq differentially expressed genes (Extended Data Fig. 4) were defined as having an adjusted *P* value < 0.05 and log_2_(fold change) > 3 times the standard deviation of all log_2_(fold change) in the comparison. For individual gene KO-versus-control phenotypes in the scRNA-seq experiments (Extended Data Figs. 5, 6 and 7), the FindMarkers function of the Seurat package (version 5) was used with default parameters other than logfc.threshold = 0 and min.pct = 0.1, and differentially expressed genes were defined as adjusted *P* value < 0.05 and absolute log_2_(fold change) > 3 times the standard deviation of the log_2_(fold change) distribution of the cluster and as described above for Fig. 7. To evaluate the significance of the enrichment of the signatures in the KOs versus the controls (Figs. 4 and 5), the GSEA preranked function of the GSEA software^99,100^ (version 4.2.3) was used, with a custom-made database of the signatures, as described elsewhere. A signature was considered significantly enriched when the NOM *P* value < 0.05 or the FDR *q* value < 0.25 and absolute NES > 1.5. For unbiased pathway analysis (Fig. 6), the pathways depicted in the figure were filtered based on the output parameters of gprofiler^101^ as described above. For statistical analysis of human samples (Fig. 8), the Shapiro normality test was used to evaluate the normal distribution of the sample, and after, according to the results, a one-way ANOVA was performed for each cytokine signature for newly generated CSF samples followed by *t*-tests or Wilcoxon tests comparing disease conditions against controls. For datasets from Schafflick et al.^32^ and Piehl et al.^33^, *t*-tests or Wilcoxon tests comparing disease conditions against controls were used. For samples from Macnair et al.^35^ and Mathys et al.^36^, the Shapiro normality test was used to evaluate the normal distribution of the sample, and after, according to the results, a one-way ANOVA or Kruskal–Wallis test per cytokine-induced signature per dataset was ran, followed by *t*-tests or Wilcoxon tests comparing all disease/lesion conditions to control (gray matter or healthy, respectively) and all clusters to the homeostatic cluster.

### Reporting summary

Further information on research design is available in the Nature Portfolio Reporting Summary linked to this article.

## Online content

Any methods, additional references, Nature Portfolio reporting summaries, source data, extended data, supplementary information, acknowledgements, peer review information; details of author contributions and competing interests; and statements of data and code availability are available at 10.1038/s41593-025-02151-6.

## Supplementary information

Supplementary Information Supplementary Methods.

Reporting Summary

Supplementary Table 1 Primer, oligonucleotides, sgRNAs and PCR protocols for cloning, library preparations and sequencing.

Supplementary Table 2 All CRISPR screen data.

Supplementary Table 3 Bulk RNA-seq data.

Supplementary Table 4 scRNA-seq cluster markers, gene signatures used for analysis for mouse and human data, and human clinical data.

Supplementary Table 5 Cell Ranger version information for human data scRNA-seq.

Supplementary Code 1 Code for all scRNA-seq analysis, mouse and human.

## Source data

Source Data Fig. 1 Source data and statistical analysis when applicable.

Source Data Fig. 2 Source data and statistical analysis when applicable.

Source Data Fig. 4 Source data and statistical analysis when applicable.

Source Data Fig. 5 Source data and statistical analysis when applicable.

Source Data Fig. 6 Source data and statistical analysis when applicable.

Source Data Fig. 8 Source data and statistical analysis when applicable.

Source Data Extended Data Fig. 1 Source data and statistical analysis when applicable.

Source Data Extended Data Fig. 2 Source data and statistical analysis when applicable.

Source Data Extended Data Fig. 3 Source data and statistical analysis when applicable.

Source Data Extended Data Fig. 4 Source data and statistical analysis when applicable.

Source Data Extended Data Fig. 6 Source data and statistical analysis when applicable.

Source Data Extended Data Fig. 7 Source data and statistical analysis when applicable.

Source Data Extended Data Fig. 8 Source data and statistical analysis when applicable.

Source Data Extended Data Fig. 9 Source data and statistical analysis when applicable.

Source Data Extended Data Fig. 10 Source data and statistical analysis when applicable.

## Data Availability

All data generated or analyzed during this study is included in the Supplementary Information, on Zenodo 10.5281/zenodo.15808138 (ref. ^102^) or via the corresponding authors upon reasonable request. Source data are provided with this paper.

## Code availability

All custom code used for analysis during this study is included in the published article as Supplementary Information or is available from the corresponding authors upon reasonable request.
