# Supplementary material for: In vivo CRISPR screen reveals regulation of macrophage states in neuroinflammation
Source: Nat Neurosci. 2025 Dec 4;29(2):493–509. doi: 10.1038/s41593-025-02151-6 (PMC12880918; doi:10.1038/s41593-025-02151-6)
Supplement: Supplementary file 1 — Supplementary Methods. [file 41593_2025_2151_MOESM1_ESM.pdf]

nature neuroscience

Supplementary information https://doi.org/10.1038/s41593-025-02151-6

In vivo CRISPR screen reveals regulation of macrophage states in neuroinflammation

In the format provided by the authors and unedited

SUPPLEMENTARY METHODS

Mouse bone marrow derived macrophages (BMDMs)

Bone marrow cells were isolated from the bones of front and hind limbs of C57BL/6 mice aged 2-5 months, by flushing with ice-cold PBS. The cell suspension was passed through a 100µm pore diameter cell strainer (Corning), resuspended in 1 mL ACK Lysing Buffer (Thermo Fisher) and incubated on ice for five minutes to lyse red blood cells. Cells were cultured in RPMI medium supplemented with 10 ng/ml M-CSF; fresh medium with M-CSF was added every two days. BMDMs were reseeded on Days 5 or 6 for polarization experiments.

In vitro differentiation of T effector cells

Naïve CD4+ T cells were purified from spleen and lymph nodes of 6-15-week-old 2D2 mice using the naïve CD4+ T cell isolation Kit (Miltenyi Biotec) as per the manufacturer's instructions. The flow-through from T cell purification was irradiated at 35 Gy (Multirad 225) and used for co-stimulation of differentiating T cells as APCs. Naïve T cells were cultured at a concentration of 1.5-2x10^6/ml in T cell medium, in the presence of irradiated APCs at a 1:5 T cell:APC ratio, with 2.5 µg/ml soluble anti-CD3 antibody (clone 145-2C11, BioXCell). Th17 cells were generating by adding 30 ng/ml of IL-6, 3 ng/ml of TGFβ, 20 ng/ml of IL-1β and IFNγ (clone XMG1.2, BioXCell) and IL-4 antibodies (clone 11B11, BioXCell) at 10 µg/ml. After 48 hours, Th17 cells were split into medium containing 10 ng/ml of IL-23. All cytokines were purchased from Biolegend, except IL-23 (Miltenyi Biotec). After four days, the production of cytokines by the T cells was assessed by intracellular cytokine staining and subsequent flow cytometry. After 5-8 days of primary culture, T cells were restimulated at a concentration of 2x10^6/ml for 48 hours in the presence of plate-bound

anti-CD3 (clone 145-2C11, BioXCell) and anti-CD28 (clone PV-1; BioXCell) antibodies at 2 µg/ml in fresh medium without any cytokines.

LSK isolation and lentiviral delivery for Bone Marrow Chimera

LSK isolation and transduction of LSK cells with lentivirus was performed as previously described[1] with minor modifications to the protocol. In short, the bones of front and hind limbs and pelvis were isolated from R26-Cas9-eGFP donor mice, flushed with ice cold PBS and ACK-lysed. LSK cells were enriched with a Direct Lineage Cell Depletion MACS isolation kit (Miltenyi) and seeded into a Retronectin- (Takara Clontech) coated flat-bottom 96-well plate (10 µg/ml per well) in StemSpan SFEM medium (StemCell Technologies) supplemented with 100ng/ml TPO (PeproTech) and 20ng/ml SCF (PeproTech). This combination of cytokines yielded the highest chimerism in myeloid cells in our hands and highest transduction of long lived progenitor cells. For viral transduction of LSK cells, HEK293T cells in 6-well plates were transfected with 1.5 µg pXPR_053-hPgk-VEX lentiviral plasmid, 1.1 µg psPax and 0.7 µg pMD2.G with 7.5µl of the transfection reagent TransIT-LT1. Viral particles were concentrated using Amicon-Ultra-15 Centrifugal Filter Units (Sigma) and immediately used to spin-infect LSK cells on the same day as isolation. 24h post infection, the LSK cells were injected i.v into recipient C57BL/6 mice that had been 2 x 4Gy (with four hours interval) irradiated (Multirad 225). Chimeric cells in the recipient mice were identified by their expression of VEX fluorophore from the viral plasmid. Unlike in the Hoxb8FL transfer, the lack of positive selection by antibiotic resistance or sorting of the infected cells meant the chimerism was mixed between non-CRISPR-edited and CRISPR-edited cells (CRISPR-edited VEX+ chimerism efficiency in **Extended Data Fig. 8**). After LSK injection in the irradiated recipients, we waited 8-10 weeks for bone marrow reconstitution before inducing EAE.

EAE induction

Active EAE was induced in 8-30-weeks-old C57BL/6 mice of both sexes under ketamine-xylazine (KX, respectively 87mg/kg and 13mg/kg) or medetomidine–midazolam–fentanyl (MMF, respectively 0.5mg/kg, 5mg/kg and 0.05mg/kg) anesthesia by immunizing them with a 1:1 volume emulsion of in-house-purified recombinant MOG1-125 (400µg) and Complete Freund's Adjuvant (Sigma) containing 10mg/ml Mycobacterium tuberculosis (BD Difco) in 250µl, injected subcutaneously in the flanks and at the base of the tail. Pertussis toxin (Sigma) dissolved in PBS (350ng) was administered intraperitoneally (i.p.) on Day 0 and Day 2 post-immunization. Disease developed 10-15 days post-immunization.

For Th17 adoptive transfer EAE, $4x10^6$ IL-17A-producing Th17 T cells were injected i.p. into C57BL/6 recipients; animals of both genders were used as donors, but male cells were injected only into male recipients. Disease developed 8-15 days post-transfer.

For the cMS model, animals of both sexes with an F1 background of C57BL6/J x BiozziABH (BiozziABH/RijHsd, Harlan Laboratories) of 2-6 months of age were used. Animals were immunized with a 1:1 volume emulsion of in-house-purified recombinant MOG1-125 (30-50µg) and Complete Freund's Adjuvant (Sigma) containing 10mg/ml Mycobacterium tuberculosis (BD Difco) in 200µl, injected subcutaneously near the axillary and inguinal lymph nodes at Day 0 and Day 7, and injected with 200 ng of Pertussis toxin (Sigma) dissolved in PBS at Days 0, 1, 7 and 8. Cortical lesion induction was performed under MMF anesthesia 3 weeks after the initial immunization. Mice were injected intracortically (coordinates: 1.2mm lateral, 0.6mm caudal to bregma, depth 0.8mm) with 2µl of a cytokine mix of 0.25 mg/ml of recombinant mouse TNF-a (R&D Systems) and 750U/ml of recombinant murine interferon-g (Peprotech) in PBS/0.1% bovine

serum albumin (BSA, Sigma). After the surgery, animals were injected with 250µl of saline/glucose 5% solution for rehydration and anesthesia antagonist (naloxone, 1.2mg/kg; flumazenil 0.5mg/kg; atipamezole, 2.5mg/kg). Analgesia (buprenorphine, 0.1 mg kg-1) was applied every 8-12h on the days following the surgery.

Animals were scored daily for weight loss and as follows for clinical symptoms: 0, no clinical signs; 0.5, partial tail weakness; 1, tail paralysis; 1.5, gait instability or impaired righting ability; 2, hind limb paresis; 2.5, hind limb paresis with dragging of at least one foot; 3, total hind limb paralysis; 3.5, hind limb paralysis and forelimb paresis; 4, hind limb and forelimb paralysis; 5, death. The first day of a clinical score above zero was considered as onset, with peak of disease designated as 2-3 days later.

Due to the variability across EAE rounds and animals, it is possible that the data shown in the Figures regarding expression of polarization markers in macrophages shows high variability across animals, especially if the data points come from independent EAE experiments. For this reason, when possible, we designed the experiments to include intra-animal controls. Thus, the CRISPR screens always included a pool of control sgRNAs transferred into all animals, the single KO validation experiments were designed as a cotransfer of control and KO cells, the Hoxb8FL Perturb Seq experiment was designed as a pool of different KOs and controls transferred into all animals, and the histological characterizations and intravital imaging experiments also consisted in co-transfer of control and KO cells. The only instances in which this was not possible were in the BM chimera experiments, in which individual animals only had one genetic perturbation, and in the Grx1-roGFP2 experiments in which differential labelling of control and KO cells in the same animal was not possible (see below).

pHrodo-labeled myelin phagocytosis assay

Myelin was isolated as previously described[2]. Myelin was labeled with amine reactive pHrodo™ Deep Red TFP ester (ThermoFisher, P35358) for 45 min at RT (protected from light) according to the manufacturer's protocol. Labeled myelin was washed with PBS and stored in aliquots at −80°C.

Tgfbr1-KO eGFP and control tdTomato or BFP Hoxb8FL-derived macrophages were differentiated for 6 days in macrophage medium with 10 ng/ml MCSF. On day 6, they were detached and reseeded on a black flat-bottom 96-well plate with clear bottom (Greiner Bio-one, 675090) at a concentration of 150.000 cells/well and polarized for 24h with a cytokine mix (TGF-β, G-MCSF, IFN-γ, TNF-α, all 15ng/ml). On day 7, the medium was changed and pHrodo-labeled myelin (4 µg/ml) was added to the co-seeded eGFP labelled *Tgfbr1*-KO and tdTomato or BFP labelled control Hoxb8FL derived macrophages. Single plane images were taken from the same three random regions per well every 15min for ~7-8h on an inverted Leica SP8X STED 3D DLS WLL confocal microscope with an HCPL FLUOTAR 10x/0.30 objective at a 512 x 512 resolution with counting mode in the HyD detectors and 12-bit configuration. Pixel size was 1.13 µm. The focus plane was found in every individual image using the Leica software Autofocus function. The following fluorescence settings were used: BFP excitation 405 nm, emission 415 – 480 nm; eGFP excitation 488 nm, emission 500 – 540 nm; tdTomato excitation 561 nm, emission 575 – 615 nm; and pHrodo excitation 633 nm, emission 650 – 700 nm. Image analysis was performed with FIJI/ImageJ with a macro as follows: the eGFP and tdTomato or BFP channels were thresholded (1-5 lower threshold depending on the image, no upper thrshold) to create a mask and a selection in which the intensity/area (IntDen) of pHrodo signal within was measured for every region and every timepoint. The three regions of

each individual well were averaged, and each technical replicate of all biological replicates was treated as an independent data point.

Cholesterol efflux assay

Tgfbr1-KO and control Hoxb8FL cells were differentiated into macrophages for 6 days with 10 ng/m MCSF and plated into poly-L-lysine coated 6-well plates. Cells were pretreated with recombinant murine TGF-β (15 ng/ml) or a cytokine mix (TGF-β, G-MCSF, IFN-γ, TNF-α, all 15ng/ml) for 24 hours, afterwards cells were exposed to purified myelin (extracted from C57 mice as described above, 5µg/ml) and TopFluor TMR cholesterol (810385P, Avanti polar lipids, 1µg/ml) for 24 hours. Myelin and TMR cholesterol were washed away and fresh medium was added; cells were let to secrete cholesterol for 24 hours. Supernatant was strained using a 40µm cell strainer to remove debris and concentrated using Amicon columns (UFC5050BK, Sigma). 10µl of concentrated medium was diluted in buffer (1:5 ethanol:PBS) and measured at 550nm (ClarioSTAR plate reader). Intracellular cholesterol content was measured by collecting washed and pelleted cells, one freeze and thaw cycle and vortex and sonication in 20µl PBS and diluted the same way as supernatant.

Histology

Animals were euthanized seven days after Hoxb8FL i.v. injection, and perfused transcardially using a peristaltic pump (velocity 6 mL/min), first with ~20 ml of Heparin (5000 U/ml) in PBS (phosphate buffered saline, 0.01M) then with 25 ml of 4% paraformaldehyde (PFA, Morphisto, 11762.05000) in PBS. Tissue was post-fixed o/n in 4% PFA. For analyses where the meningeal

compartment was relevant (**Figs. 5** and **6** and **Extended Data Figs. 1** and **7**), after post fixation, the whole spinal column was cleared of muscle as much as possible and then incubated for one week in 0.3M EDTA in water, pH 7.5, with buffer exchanges every 2-3 days until full decalcification. For analyses where the meningeal compartment was not relevant (**Fig. 1**), after post fixation the spinal cord was isolated. Then, the tissue cryoprotected in 30% sucrose for 72 hours. Depending on the staining, a free-floating method or staining on slide was used to visualize selected markers. For free floating-stainings (**Fig. 1**), samples were embedded in Tissue-Tek (O.C.T. Sakura Finetek Europe B.V.), frozen immediately to -20°C and cut into 40 μm-thick coronal sections on a cryostat (Leica, CM1950). Sections were then rinsed with 1xPBS and incubated for 30 minutes in 10% blocking solution (10% fish gelatin (Sigma-Aldrich, G7765), 10% FBS (Thermo Fisher Scientific, 10500064) and 10% BSA (Sigma, A7030) in 0.5% Triton X in 1xPBS) at room temperature; anti-CD11b antibody conjugated to Alexa Fluor 647 (Biolegend, catalog no. 101218) was diluted 1:100 in 1% blocking solution in 0.5% Triton X in 1xPBS and incubated with samples o/n at 4°C, before finally staining with DAPI (1:10,000, Invitrogen, catalog no. 62247) for five minutes. Sections were mounted with Vectashield (VectorLaboratories, catalog no. H-1000-10). For on-slide stainings (**Figs. 5** and **6** and **Extended Data Figs. 1** and **7)**, full vertebrae were pre-cut with a scalpel and embedded in Tissue-Tek at room temperature for one hour to allow for full penetration into the spinal canal, then frozen at -20°C and cut in 25-30 μm-thick coronal sections onto SuperFrost Ultra Plus Gold adhesive slides (Fisher Scientific, 11976299). Samples were dried for 30 minutes at RT. Sections were then rinsed twice with 1xPBS and incubated for 1 hour in 10% blocking solution (10% fish gelatin (Sigma-Aldrich, G7765), 10% FBS (Thermo Fisher Scientific, 10500064) and 10% BSA (Sigma, A7030) in 0.5% Triton X in 1xPBS) at room temperature. After blocking, sections were washed once with 1x PBS for 5 minutes and then incubated overnight at 4°C with the relevant primary antibodies diluted in 1% blocking

solution in 1x PBS containing 0.1% Sodium Azide: rabbit anti-Laminin (LAMA1) (Sigma-Aldrich, # L9393, 1:200), rat anti-LYVE1 (ALY7) (Thermo Fisher Scientific, #14-0443-82, 1:100), rat anti-CD107a (LAMP-1) (BioLegend, # 121602, 1:200), rabbit anti-Fibronectin (Sigma-Aldrich, # AB2033, 1:100), rat anti-GPNMB (CTSREVL) eFluor™ 660 (Thermo Fisher Scientific, #50-5708-82, 1:100), chicken anti-MBP (Thermo Fisher Scientific, # PA1-10008, 1:200), rabbit anti-Perilipin-2 (Plin2) (Novus-Biologicals, # NB110-40877, 1:200), rat anti-CD206 (Bio-Rad, # MCA2235, 1:100), and rat anti-IBA1 (Synaptic Systems, #HS-234017, 1:200). For Plin2 staining, acidic antigen retrieval was performed with prewarmed citric acid (pH 6) at 70°C in a water bath for 10 minutes prior to blocking. Sections were then incubated with citric acid for an additional 15 minutes at room temperature and washed three times, 10 minutes each with 1xPBS. For MBP staining, sections were treated with ice-cold 100% methanol for 10 minutes, followed by a 5-minute wash with ice-cold 1xPBS before blocking. Following primary antibody incubation, the sections were washed three times for 10 minutes each with 1xPBS, then incubated with relevant secondary antibodies diluted 1:1000 in 1x PBS for two hours at room temperature in the dark: goat anti-rabbit Alexa Fluor™ 647 (Thermo Fisher Scientific, # A-21245), donkey anti-rat Alexa Fluor™ Plus 647 (Thermo Fisher Scientific, # A48272), donkey anti-rat Alexa Fluor™ Plus 405 (Thermo Fisher Scientific, # A48268), and goat anti-chicken Alexa Fluor™ 647 (Thermo Fisher Scientific, # A-21449).For neutral lipid staining, HCS LipidTOX™ Deep Red Neutral Lipid Stain (Thermo Fisher Scientific, # H34477, 1:200) was used during the secondary antibody incubation. After the secondary antibody staining, sections were washed either three times, 10 minutes each, with 1x PBS, or stained for 10 minutes with DAPI (InvitrogenTM, # 62247, 1:10.000) followed by two 10-minute washes with 1x PBS. The sections were then mounted with Vectashield (Vector Laboratories, # H-1000-10).

Stained tissue sections were imaged with a Leica SP8 confocal microscope, using a 40x/1.30 NA oil immersion objective, or with an RS-FV4000 confocal microscope, using a 20x/0.8 NA air objective. Image pixel size was 284 nm for **Figs. 1** and **5** LipidTox/Lamp1 stainings, 621 nm for Fig. **5** other stainings, **Extended Data Figs. 1** and **7,** and 569 nm for **Fig. 6**; z-step size was 1 µm for lesion localization and functional marker evaluation (**Figs. 1** and **5**, **Extended Data Figs. 1** and **7**) and 3 µm for the pia localization (**Fig. 6**). The following fluorescence settings were used: DAPI / Alexa Fluor-405 excitation 405 nm, emission 415 – 480 nm; eGFP excitation 488 nm, emission 500 – 540 nm; tdTomato excitation 561 nm, emission 575 – 615 nm; Alexa Fluor 647 / LipidTox excitation 633 nm, emission 650 – 700 nm. Image analysis for **Figs. 1** and **6,** and **Extended Data Fig. 1i** was performed with FIJI/ImageJ as follows: first lesion areas were defined by outlining densely infiltrated areas based on z-projections of DAPI channel or the Hoxb8FL-gray colored cells. For the lesion distribution, lesions were segmented into layers of 30 µm depth, starting from the pial surface, and endogenous macrophages (DAPI+/CD11b+/eGFP-) and transferred macrophages ((DAPI+/CD11b+/eGFP+) were counted in the z-stack for each depth segment, using the CellCounter plugin. Data are represented as % of total endogenous or transferred macrophages residing in a specific depth segment. One data point represents one mouse. A minimum of n=8 lesions was analysed per mouse. For the KO distribution by compartment phenotype, other anatomical regions of the spinal column were ROIed in addition to lesions (pia lesion and non-lesion, parenchyma lesion and non-lesion, bone marrow). The pia was defined based on the laminin staining of the basal membrane of the meningeal layer. eGFP and tdTomato channels were thresholded to define eGFP+ and tdTomato+ areas. The same thresholds were used for all images. Total area for each ROI was measured as well as eGFP+ and tdTomato+ area within it. To eliminate bias from transferring unequal numbers of eGFP+ and tdTomato+ cells the eGFP/tdTomato area ratio of each ROI was normalized to the ratio in the bone marrow, considered the neutral

compartment as no KO showed a migration phenotype when exiting the bone marrow (**Fig. 1f**). For the analysis of **Extended Data Fig. 1i**, the Iba1+ tdTomato- (endogenous) and the tdTomato+ (Hoxb8FL-derived cells) areas were measured for the lesions and the pia immediately adjacent to the lesions, and the proportion of area occupied by the cells in the pia or in the parenchymal lesion out of the total area occupied by the cells was calculated for quantification. Image analysis for **Fig. 5** and **Extended Data Fig. 7** was performed as follows: Images were exported to Imaris software (V9.7.2 Bitplane, Oxford Instruments group). Fluorescent signal was first thresholded to remove background noise based on the signal's histogram. The same threshold was applied equally to all images within the same group. The threshold was applied as follows: Fn1 (1250), Gpnmb (35), Lamp1 (60), LipidTox (14), Plin2 (1000). Hoxb8FL cells were then identified based on their expression of eGFP (KO) or tdTomato (control). Surfaces were created on these cells with a surface grain size of 0.5 µm for all images and touching cells were separated using the Region Growing algorithm with a seed point diameter of 6-7.5 µm. Quality threshold was applied on each image and kept consistent within the groups. Surfaces were filtered by volume (between 100 µm3 and 8250 µm3) or by number of voxel number (300-1000) to discard any debris or misrendered cells. For volume or puncta analysis within Hoxb8FL cells, rendered surfaces on eGFP and tdTomato were used to mask the desired channel to extract only the signal within Hoxb8FL cells. Then, surfaces were created on both masked channels with a surface grain size of 0.5 µm and with a consistent quality threshold within the groups (LipidTox 25; Gpnmb 3.3). The absolute intensity for the signal of interest was then measured per control or KO cell, and averaged by individual tissue section and then by animal. Whether the intensity was normalized by cell volume or not is indicated in the individual figure panels. For visualization and statistical analysis, one data point represents one mouse. One to three sections from different vertebrae were analysed per mouse for each staining.

In vivo imaging of Hoxb8FL-derived macrophages

For *in vivo* evaluation of oxidative bursting, Grx1-roGFP2[3], a redox biosensor, was expressed in *Ifngr1*-KO, *Tnfrsf1a*-KO, and control Hoxb8FL cells. These cells were exposed to 10 ng/ml M-CSF for two days *in vitro* before being transferred into EAE-induced animals on day 8. For *in vivo* evaluation of lysosomal acidification, mTFP1-mCherry, a pH-sensitive biosensor for probing lysosomal pH[4], was expressed in *Tgfbr1*-KO and control Hoxb8FL cells. After two days of *in vitro* exposure to 10 ng/ml M-CSF, the cells were co-transferred into EAE-induced animals on day 9.

MMF anesthetized mice were positioned on a heating pad set for 37°C throughout the procedure (Stoelting Rodent Warmer 2x, Ugo-Basile). The lumbar dorsal spinal cord was then surgically exposed by performing a laminectomy of two lumbar vertebrae and durectomy, continuously superfused with artificial cerebrospinal fluid (aCSF, 148.2 mM NaCl, 3.0 mM KCl, 1.4 mM CaCl2, 0.8 mM MgCl2, 0.8 mM Na2HPO4 and 0.2 mM NaH2PO4), and stabilized horizontally using pins screwed onto a metal holding frame (custom made). A low-meting point agarose and play-doh well was created to contain the aCSF. Animals were imaged with a Leica SP8 WLL DIVE FALCON confocal microscope using a 20x/0.75 NA water immersion objective, with a 1.08 µm pixel size, counting mode in the HyD detectors and 12-bit configuration, and a 1.5 µm step size for the z-stack. The following fluorescence settings were used: CellTrace™ Violet excitation 405 nm, emission 412 – 475 nm; CellTrace™ Yellow excitation 552 nm, emission 677-607 nm; CellTrace™ Far Red excitation 633 nm, emission 695-780 nm for Grx1-roGFP2 experiments and 645-710 nm for mTFP1-mCherry experiments; oxidized roGFP2 excitation 405 nm, emission 500-530 nm; reduced roGFP2 excitation 488 nm, emission 500-530 nm; mTFP1 excitation 462 nm, emission 492-549 nm; mCherry excitation 570 nm, emission 580-624 nm. Two independent experiments

with several mice were done per KO, always including control and KO transferred animals to minimize technical variation. For *Ifngr1*-KO and *Tnfrsf1a*-KO Grx1-roGFP2 redox sensor experiments, initially animals were co-transferred control and KO cells labelled in either CellTrace™ Far Red or CellTrace™ Yellow to indicate the genotype, however CellTrace™ Yellow was impossible to separate from autofluorescent debris signals and thus only the Far Red labelled cells were analysed and no intra animal paired analysis was possible. For *Tgfbr1*-KO mTFP1-mCherry lysosomal acidification sensor experiments, control and KO cells were labelled respectively with Far Red and Violet in half of the mice and vice versa in the other half of the mice of each experimental round to control for inter-animal variability and potential effects of the different CellTrace™ versions. Thus, intra-animal paired analysis was possible.

For analysis, images were exported to Imaris software (V9.7.2 Bitplane, Oxford Instruments group). A median filter with a filter size of 3 x 3 x 3 was applied to smooth the signal prior to further processing. Then, surfaces were created on the CellTrace™ Far Red channel (Grx1-roGFP2 experiments) or on the mCherry channel (mTFP1-mCherry experiments) with a surface grain size of 1 µm for all images and touching cells were separated using the Region Growing algorithm with a seed point diameter of 8 µm. Surfaces were filtered manually to remove debris based on incompatible co-expression patterns of the different imaged fluorophores, and then based on volume (surfaces smaller than 50 μm^3 or being a statistical outlier bigger than the Q3 + 1.5*IQR of the range of volumes in the experiment were discarded). The absolute intensity for the signals of interest (roGFP2, mTFP1, mCherry and CellTrace™ Violet and Far red) was then measured per surface. For the Grx1-roGFP2 sensor experiments, the 405 excitation / 488 excitation ratio was calculated per cell. For the mTFP1-mCherry experiment the cells were first assigned to control/KO based on the signal intensity for the CellTrace™ such that all cells with the bottom 2% of signal intensity for the CellTrace™ in each image were discarded, and the remainder of the cells were

assigned to one color or the other if the intensity of the color normalized to the cell volume and scaled from 0 (no signal) to 1 (highest possible intensity) was at least twice the intensity of the other color normalized to the cell volume and scaled from 0-1. Then, the mCherry / mTFP1 ratio was calculated per cell. For bin plots (**Fig. 4e** left and **f** left and **Fig. 5j** left), the whole range of values of the sensor ratio across all experiments was divided into 10 equally-sized bins, and the proportion of cells that fall within each bin per animal was calculated. For the proportion of cells above the 75th percentile / 3rd quantile (Q3) of the ratio, the Q3 was calculated per independent experiment (always containing KO and control cells, to account for possible technical variations between rounds in sensor intensity measurements) as a single value and the percentage of cells above that value was calculated per animal. For representative image preparation to enhance cell visibility, either the CellTrace^{TM} Far Red channel or the mCherry channel were thresholded to create a mask and exclude the background outside of the mask of all the channels, and the images were edited with only the information inside of the masks. To plot the sensor ratios, the image calculator function of Fiji was used, dividing one channel by the other after having filtered out the background outside the masked area.

As Hoxb8FL cells can differentiate into neutrophils in a low proportion, after the *in vivo* imaging animals were processed for FACS analysis as mentioned above to control for the percentage of Hoxb8FL-derived neutrophils. For the *Ifngr1*-KO Grx1-roGFP2 experiment there was 17.8 % ± 13.2 % of neutrophils, for the *Tnfrsf1a*-KO Grx1-roGFP2 11.7 % ± 6 %, and for the *Tgfbr1*-KO mTFP1-mCherry 11.8 % ± 7 %.

Hoxb8FL *in vivo* migration tracking

Movies were taken every 1 minute interval in a 1.5 μm step size z-stack for a total of 15 minutes to 1 hour. Images were exported to Imaris software (V9.7.2 Bitplane, Oxford Instruments group). A reference frame was added at the position of the anterior spinal vein to correct for positional changes during *in vivo* imaging and drift was corrected with the translational and rotational drift correction algorithm within Imaris. Hoxb8FL movements were tracked using Imaris surface object algorithm based on the CellTrace™ channels to create tracks for each cell. Surface grain size of 1 μm was used for all images and touching cells were separated using the Region Growing algorithm with a seed point diameter of 8-10 μm. Quality threshold was applied on each image individually and surfaces were filtered by number of voxels (75) to discard any debris. Tracking was then performed with Autoregressive Motion algorithm with a maximum distance of 8 μm between two time points and with a maximum gap size of 3. Tracks exhibiting less than 180s (3 time points) duration in the imaged field were excluded. All cell tracks were manually corrected using the manual editor when misdetection or non-detection were observed.

NGS sgRNA library preparation

Genomic DNA (gDNA) from iNOS+, Arg1+ or negative sorted Hoxb8FL-derived macrophages was isolated with the DNeasy Blood and Tissue Kit (Qiagen). Library PCR for Illumina was prepared as previously described[5]. A one-step PCR amplification was performed with Q5 High Fidelity DNA Polymerase with Fwd-Lib (mix of eight staggered primers) and Rev-Lib (consists of 8 bp of unique barcode) primers for a total of 25 cycles. Illumina adapters were introduced together with the amplification primers. All primer sequences are listed in **Supplementary Table 1**. The amplified DNA amplicons were purified with SPRIselect (Beckman Coulter) at a ratio of 1 DNA:0.8 beads and eluted in nuclease-free water. The presence of ~250 bp DNA amplicons was

confirmed and the concentration was measured with an Agilent Bioanalyzer using DNA 1000 Chips. Library samples were sent to The Laboratory for Functional Genome Analysis (LAFUGA) at the Gene Center Munich for single-end 60 bp sequencing on a NextSeq 1000. All results from the CRISPR screens are included in **Supplementary Table 2**.

3` bulk mRNA sequencing

For *in vivo* bulk RNA sequencing, Hoxb8FL-derived and endogenous monocytes (CD11b+ Ly6G- from blood, and fixed iNOS+ and Arg1+ macrophages (CD11b+ Ly6G-) from spinal cord, were sorted for purity and washed twice with PBS before RNA isolation. For *in vitro* bulk RNA sequencing, BMDMs were incubated with 20ng/ml of the respective cytokines (IFN-γ, TNF-α, TGF-β, GM-CSF, IL-4) for six hours. Total RNA from cells was isolated with either a RNeasy Plus Mini (Qiagen) or a Micro (Qiagen) (for less than 100k cells) kit according to the manufacturer's protocol. For isolating RNA from fixed cells, RNasin Plus (Promega) RNase inhibitor was present at a 1:1 dilution during fixation, antibody labeling and sorting. Proteinase K treatment in AL buffer (Qiagen DNA micro kit) was performed for one hour at 56 °C prior to the start of isolation with Qiagen RNeasy kits.

For 3` bulk mRNA sequencing, the library was prepared from total RNA using the Collibri 3' mRNA Library Prep Kits for Illumina Systems (Thermo Fisher). The amplification of transcripts was confirmed with an Agilent Bioanalyzer using DNA 1000 Chips and sent to LAFUGA (Gene Center, LMU Munich) for single-end 60 bp sequencing on a NextSeq 1000.

scRNA library preparation

The 10x Genomics Chromium Next GEM Single Cell 3' v3.1 (Dual Index) was used for library preparation for WT cells. 10x Genomics Chromium Next GEM Single Cell 5' v2 combined with Feature Barcode technology for CRISPR screening was used to detect sgRNAs in Perturb Seq experiments. Library preparation was done according to the manufacturer's protocols. Library traces were confirmed by Agilent Bioanalyzer on DNA 1000 Chips and samples were sent to LAFUGA (Gene Center, LMU Munich) for sequencing on NextSeq 1000 or NextSeq 2000 with settings recommended by 10x.

Bioinformatic analysis

CRISPR library analysis

The Galaxy platform[6] was used for data analysis. Je-Demultiplex-Illu[7] was used for de-multiplexing raw fastq files, followed by Cutadapt[8] and Trimmomatic[9] to extract the 20 bp sgRNA sequence. MAGeCK[10] count (version 0.5.7.1+) was used to obtain single sgRNA counts. The samples were normalized with R[11] (version 4.3.1+) after a 50 raw count threshold using the geometric mean per sgRNA for normalization. sgRNAs with counts in only one replicate were still considered for analysis, as the individual sgRNAs are then averaged for each gene. The MAGeCK test was run without normalization or zero removal since the input had already been normalized in R, and otherwise default parameters on Galaxy, uploading the information about the control Non-Targeted sgRNAs for noise correction during the MAGeCK test run. All further data processing was done with R. All results from the CRISPR screens are included in **Supplementary Table 2**.

CRISPR library significance

To calculate the log2(Fold Change) noise threshold per comparison, 5000 control "gene" log2(Fold Changes) were calculated by calculating the median of three Non-Targeted sgRNAs sampled with replacement from the Non-Targeted pool values of each sample, and then calculating the log2(Fold Change) for the specific comparison. This gave the noise distribution of control genes, and log2(Fold Changes) were considered meaningful when their absolute value was greater than three times the standard deviation of the control noise log2(Fold Change) distribution. To consider a gene as significant from the results of the CRISPR screen, in addition to the above mentioned log2(Fold Change) threshold, the gene had to have a p-value < 0.05 ("neg|p-value" for negative log2(Fold Change) genes and or "pos|p-value" for positive log2(Fold Change) genes) and an fdr value < 0.05 ("neg|fdr" or "pos|fdr" respectively), and at least two good sgRNAs ("neg|goodsgrna" or "pos|goodsgrna", defined as sgRNAs with an individual log2(Fold Change) of the same sign as the gene log2(Fold Change)).

Bulk RNAseq analysis

Fastq files were processed in Galaxy[6], aligned with RNA STAR[12] (version 2.7.2b) to the reference genome mm10 with default parameters and without trimming, followed by HTSeq-count[13] (version 1.0.0) and DESeq2[14] (version 2.11.40.7+galaxy1) to calculate differentially expressed genes, with "estimateSizeFactors" = poscounts and otherwise default parameters. Batch correction per animal was performed when applicable. All further analysis was run with R[11] (version 4.3.0+). Monocyte and macrophage genes were selected based on the literature.

Significant DEGs were defined as those with an adjusted p-value < 0.05 and an absolute log2(Fold Change) greater than three times the standard deviation of the log2(Fold Changes) distribution of the comparison. All results from the bulkRNAseq are included in **Supplementary Table 3**.

scRNAseq preprocessing

Raw fastq files were demultiplexed in Galaxy platform[6] using the Je-Demultiplex tool[7] based on the information from the barcodes used in the library prep and aligned to the mouse genome mm10 with 10x Genomics cellranger multi (version 6.1.2), including introns, and otherwise default parameters. For the Hoxb8FL and BM chimera experiments, the sgRNA information was given as a Feature Reference csv file as per the requirements of cellranger multi. The following genes were manually added to the reference to help with Hoxb8FL-derived cell and chimeric cell detection: eGFP, BCL2, and Cas9, both for name and sequence, and a new reference was created using cellranger mkref. The cellranger output feature and barcode matrices were then processed with the R package Seurat[15-19] (version 5.0.0). The data from each experiment were processed individually first. After loading the data, background RNA correction was applied using the SoupX[20] R package (version 1.6.2), followed by normalizing RNA and GDO assays with NormalizeData, computing the variable features by FindVariableFeatures with the "vst" method, scaling the data to all genes by running ScaleData with features = rownames(object). Then, RunPCA was run. For all objects, 35 dimensions were chosen for running the following functions FindNeighbours and RunUMAP.

GDO assay feature calling per cell was assigned manually. For the GDO assay, the proportion out of total detected sgRNA counts was calculated for each detected sgRNA, and the top detected sgRNA was assigned if it exceeded 28% of total counts of the cell in the Hoxb8FL dataset and 40% of total counts of the cell in the BM chimera dataset. These percentages were determined visually to be the noise threshold of the sgRNA detection. This resulted in each individual cell containing the information for the sgRNA it was transduced with, which allowed us in further analysis to always individually match transcriptome and KO information. The cells with sgRNAs targeting the same gene, as there were two sgRNAs per gene, were grouped in a gene KO group and analysed together. The sgRNA information used for the Perturb Seq experiments is included in **Supplementary Table 1**.

For quality control (QC), each object was then independently clustered after the pre-processing steps described above with high resolution (res = 6-7) and the clusters were visually evaluated for nFeature, nCount and percent.mito (calculated based on mitochondrial genes) distributions with violin plots. Any clusters that were outliers in any of these metrics were discarded whole, that is, too high or too low nFeature and nCount compared to the bulk of the dataset, and too high percent.mito were discarded.

To define Hoxb8FL-derived cells, the expression of EGFP, *Hoxb8FL*, BCL2 and Cas9 were individually assessed per cell, and the cell was considered a Hoxb8FL-derived cell if it had detected expression of any of those genes. To account for the sparsity of the scRNAseq data, if the cell had expression of one out of the three genes it was already considered a Hoxb8 cell, as for example the EGFP transcript is never detected in a 100% of the FACS sorted fluorescence+ cells. This was necessary as endogenous cells had also been sorted in the same experiment to control for integration of Hoxb8FL-derived cells and the endogenous population. No such filter was necessary in the chimera experiment, as only Vex+ cells were sorted. For both chimera and Hoxb8FL experiments, all chimera- and Hoxb8FL-derived cells without detected sgRNAs were discarded for downstream analysis, and in the Hoxb8FL experiment all non-Hoxb8FL-derived cells were discarded post-integration from further analysis.

After preprocessing and QC, the total number of cells was 15,626 for the WT dataset, 11,249 for the Hoxb8FL dataset (3,535 control, 2,287 *Ifngr1*-KO, 2,800 *Tnfrsf1a*-KO, 1,964 *Csf2ra*-KO and 663 *Tgfbr1*-KO) and 32,793 for the bone marrow chimera dataset (6,145 control, 5,399 *Ifngr1*-KO, 5,207 *Tnfrsf1a*-KO, 8,675 *Csf2ra*-KO and 7,367 *Tgfbr1*-KO); 13,993 cells for the mouse CSF object; and 31,895 cells for the cMS object.

Once all samples had been individually QC-ed, they were integrated at the 10x lane level across samples using FindIntegrationAnchors with 40 dimensions and IntegrateData from the Seurat package, except the CSF object, which was integrated with harmony[21] RunHarmony. The object was then renormalized and rescaled, and the dimensionality reduction and UMAP recalculated with 40 dimensions. The cluster identity assignation was done on the WT object for the spinal EAE datasets (WT, Hoxb8FL and Chimera). First, the resolution for clustering was fixed at 1.1 by checking the cluster divergence and convergence in a range of resolutions with clustree[22] (version 0.5.1). Then the cell identity was determined by expression of classical cell specific markers (**Extended Data Fig. 5a**). The cluster names were then transferred to the full integrated object by TransferData of Seurat. The CSF and cEAE objects' cluster names were manually assigned per object. All results from the scRNAseq are included in **Supplementary Table 4** and as supplementary materials.

Cluster markers and KO vs control DEGs

Cluster markers for the WT EAE subset and the control Hob8 and chimera subsets were computed with the FindAllMarkers function, and KO vs control DEGs with the FindMarkers function. The logfc.threshold was set to 0 and min.pct to 0.1. We observed some differences in the frequency of the Cxcl10+ macrophage clusters across datasets (21.6 % of total monocyte/macrophages in the WT, 11.8 % in the Hoxb8FL-derived cells and 14.9 % in the bone marrow chimera dataset, **Fig. 3** and **Extended Data Fig. 5**), so we confirmed that the top genes upregulated in Cxcl10+ macrophages compared to the rest of the monocyte/macrophages show similar expression patterns across datasets (data not shown) and that the genes regulated in the KOs vs control positively correlate between the Hoxb8FL and chimera datasets (data not shown), suggesting the regulation of these cells is not affected.

Pseudotime analysis

Pseudotime was calculated for the integrated object using monocle3[23-25] (version 1.3.4), with the Monocyte cluster as root cells.

Population density differences by KO

To calculate the population density differences between the KOs and the controls, the data from both Hoxb8FL-derived KOs and controls and chimeric KOs and controls was used in combination. Then, using the UMAP embeddings for both KO and control, the kde2d function from the MASS[26] package was applied to perform kernel density estimation, and the difference was computed KO – control.

Correlation of cluster markers across WT, Hoxb8FL and chimera experiments

For calculating the correlation between cluster markers in any two experiment comparisons, a linear model was used.

Signature scores

To calculate the signature expression, a customized version of the AddModuleScore function was used, where the normalization to control genes was removed (see available code for details, change consisted in commenting out "- ctrl.scores" in line 100 of the function). Thus, the average expression of the genes belonging to each signature was calculated per cell per signature, and this value was used for plotting in the FeaturePlots.

To evaluate the significance of the enrichment of the signatures in the KOs vs the controls, the GSEA pre-ranked function of the GSEA software[27,28] (version 4.2.3) was used, with a custom-made database of the signatures. GSEA was run without collapse in classic mode and min. size = 3, and otherwise default parameters. A signature was considered significantly enriched when the NOM p-

value < 0.05 or the FDR q-value < 0.25 and absolute NES (normalized enrichment score) > 1.5. GSEA was run for all individual cluster KO vs control comparisons in the Hoxb8FL and chimera datasets, as well as for the full object KO vs control ("All" in Figs. 5 and 6), without clustering separation, but excluding non-macrophage clusters form the analysis.

A signature was considered to have a phenotype in the KO if the "All" KO vs control comparison was significant in the same enrichment direction in both the Hoxb8FL and the chimera data. In the interest of space only the Hoxb8FL data is shown in the figures, the BM chimera data is not.

The genes included in the individual functional signatures are included in **Supplementary Table 4.**

*Plotting of signatures across KOs (**Figs.** 4 and **5**)*

To better illustrate the GSEA per cluster that was used to determine whether signatures were significantly regulated by KO, the distribution of Log2(Fold Changes) of the genes of the signature was plotted, as it is the data on which the GSEA runs. Therefore, the 10-90 in steps of 10 quantile values were computed per cluster and per signature. The quantile values were then plotted as semi-transparent shadings spanning the most extreme to the closest to the median, 10-90, 20-80, etc, with the quantile 50, the median, plotted as a single line. Overall shading that falls preferentially below zero indicates negative enrichment in the KO compared to the control; while shading preferentially above zero indicates positive enrichment in the KO compared to the control.

For **Fig. 4** and **5**, only signatures in which at least one KO had a significant phenotype in both Hoxb8FL and chimera experiments were chosen. All the data included in the figures however comes from the Hoxb8FL results, including the statistical significance.

Correlation of the signatures across experiments

To calculate the correlation coefficient of the signatures across WT, Hoxb8FL and chimera experiments, each signature value was averaged for all the cells of a cluster, and the Pearson correlation was computed for any given signature values across clusters between two experiments. For the correlation of KO phenotypes between the Hoxb8FL and chimera experiments, the Log2(Fold Changes) with the FindMarkers function (logfc.threshold = 0, min.pct = 0.1) were computed in the whole Hoxb8FL-derived of chimeric-derived KO vs control populations and the gene values were correlated.

Differentially expressed genes and neuroinflammation cytokine-specific gene signatures

DEGs per KO were calculated as explained above, using the FindMarkers (logfc.threshold = 0, min.pct = 0.1) function. To define a gene that was differentially regulated in the KO vs control for a given cluster, the gene had to have the same sign of Log2(Fold Change) in the Hoxb8FL and the chimera results, and the absolute Log2(Fold Change) had to be greater than twice the standard deviation of the Log2(Fold Change) distribution of its condition (either Hoxb8FL or chimera) in either Hoxb8FL or chimera, or both, results. DEGs were considered unique to the cluster when they were regulated only in one cluster, and common if they were regulated in more than one cluster. The common DEGs were considered as the KO vs control signature, and when downregulated in the KO vs the control, or "neuroinflammation cytokine signature". The genes comprising these signatures are included in **Supplementary Table 4.**

To plot the overlap of DEGs between different KOs, the R package igraph (ref, v1.5.1) was used.

Unbiased GO term pathway analysis

The gprofiler webtool g:GOSt[29] tool was used on the cytokine-induced gene lists, with Mus musculus organism and default parameters. The results were filtered based on an adjusted p value

< 0.01, having an intersection size of at least five. Terms with more than 2000 genes were excluded. Only GO Biological Processes and GO Molecular Function terms were plotted.

Reanalysis of Cui et al.[30]

The macrophage object was downloaded from the interactive web portal (https://www.immune-dictionary.org/app/home in the downloads tab). Our cytokine-induced gene lists were used with the customized AddModuleScore function described above to detect in which samples they were more highly expressed. Some less-classical cytokines were excluded for plotting. Then, per sample, the mean ± standard deviation was plotted.

Human data

Study design

Clinical characteristics are summarized in **Supplementary Table 4**. CSF sampling was performed either to confirm the diagnosis of Relapsing-Remitting MS according to the 2017 revision of the McDonald criteria[31] (RRMS), for diagnostic evaluation of suspected Radiologically Isolated Syndrome (RIS) according the proposed RIS-diagnostic criteria[32], or for therapeutic CSF removal in people with Idiopathic Intracranial Hypertension (IIH). Persons with RRMS had not received prior disease modifying therapy. They had experienced a clinical relapse in a median of 59 days (interquartile range 21,5 – 170,25) before CSF sampling. 5 patients had received steroid-based relapse therapy up until a median of 92 days (minimum 24, maximum 1621) in advance. RRMS in all individuals was considered "active" according to Lublin *et al.*, 2014[33], as either a clinical relapse, or a new or unequivocally enlarging or Gadolinium-enhancing lesion on brain or spinal cord magnetic resonance imaging was present within 365 days prior to sampling. Of 7 people with

white matter lesions referred for CSF analysis to evaluate RIS 5 showed signs of intrathecal immunoglobulin-synthesis, as shown by CSF-restricted oligoclonal bands. 4 of which showed signs of an active MRI (new or enlarging T2 hyperintense lesion, or Gadolinium-enhancing lesion). The diagnostic criteria for RIS in line with Okuda *et al.*, 2009[34], were met in 3 cases, 4 were detected by the revised criteria[32]. CSF samples from five individuals diagnosed with idiopathic intracranial hypertension who received therapeutic lumbar puncture were included. Samples were collected at the Institute of Clinical Neuroimmunology at the LMU Klinikum Munich, Germany. Recruitment of individuals who fulfilled the inclusion criteria took place from August 2020 to January 2021. Collection of CSF was approved by the local ethics committees of the LMU, Munich (ethical vote: 163-16, equivalent of the Institute Review Board (IRB) number for the ethics committee). Written informed consent was obtained from all subjects according to the Declaration of Helsinki. A total of five people with IIH, seven people with RIS and 22 people with MS donated samples that were sequenced for this study.

Cerebrospinal Fluid (CSF)

Samples of human CSF (3-6 ml) were processed within one hour of lumbar puncture. After centrifugation at 300 g for 10 minutes, the cell pellet was incubated in a 2 ml tube with TotalSeq-C Antibodies: anti-human CD4, anti-CD8A and mouse IgG1 isotype control (Biolegend, 0.5 µg of each). Then, the Cell Surface Labelling Protocol from 10x Genomics was followed, but with all centrifugations done at 300 g for 10 minutes. All cells were loaded on the 10x Chip, with a maximum target cell number of 10,000.

10x library preparation and sequencing

Further processing followed the manufacturer's protocol using the Chromium Next GEM Single Cell VDJ v1.1. The Feature Barcoding technology for Cell Surface Protein steps was also

performed. Libraries were sequenced on an Illumina NovaSeq6000 S4 using read lengths of 150 bp read 1, 8 bp i7 index, 150 bp read 2.

Processing of single cell sequencing data

Sequencing results were demultiplexed and aligned to the human GRCh38 reference genome using Cell ranger (10X Genomics, v4.0.0 and 5.0.1, see **Supplementary Table 5**). Cell barcodes with unique molecular identifier (UMI) counts that reached the threshold for cell detection were included in subsequent data analysis.

Public CSF Datasets

In addition to the internal dataset, two public human CSF datasets were used to evaluate the expression of the identified signatures in human cells. To compare to MS samples, the dataset by *Schafflick et al.*[35] was downloaded, consisting of six MS cases with six IIH cases as controls. To compare to a non-autoimmune neurodegenerative disease, the dataset from *Piehl et al.*[36] was used, containing 14 cases of mild cognitive impairment or Alzheimer's disease, and 45 control samples.

CSF data processing

All data analysis was performed using the software packages Seurat[15-19] (version 4.3.0) for general processing and harmony[21] (version 0.1) for sample integration. Data from all samples were merged and data from all cells with more than ten percent mitochondrial reads were removed. Genes that were not expressed in any of the three datasets were dropped. To perform quality control and cluster identification, four rounds of processing were performed, each followed by selection of cells of interest. Each round consisted of running NormalizeData, FindVariableFeatures, ScaleData and RunPCA with default parameters, followed by RunHarmony, RunUMAP and FindNeighbors with the top 20 principal components. The RunHarmony function was run with the parameter

“group.by.vars” set as the individual person metadata column. The clustering resolutions used for the four rounds were 0.1, 0.1, 0.03 and 0.03. Clusters were evaluated on the distribution of the percentage of mitochondrial UMIs, total number of UMIs, as well as coexpression of multiple cell-type-specific markers, to allow the removal of low-quality cells and clusters of unclear identity. Cluster identification for removal was performed by visual inspection, using the Seurat functions DotPlot and VlnPlot with parameter “add.noise” as False. Monocyte clusters were selected based on the expression of ITGAM, CD14 and FCGR3A and subsetted. Clusters expressing either CD3E or CD79A were removed as potential doublet clusters. A total of 10,729 cells (2,671 from our own data, 1,402 from *Schafflick et al.*[35] and 6,656 from *Piehl et al.*[36]) were used for further analysis.

Public brain parenchyma datasets

To evaluate the signature behavior in human parenchymal lesions, the public dataset of *Macnair et al.*[37] was analysed. This consisted of data from 173 single nuclei from different MS lesion types (n = 55 donors) in the brain parenchyma, as well as samples from healthy controls (n = 30). To compare to a non-autoimmune neurodegenerative disease, the dataset from *Mathys et al.*[38] was used, containing 48 samples of individuals with diagnoses ranging from no cognitive impairment to Alzheimer’s disease. Based on the cognitive status at time of death (cogx) for each individual, people with a value of 1 (no cognitive impairment) were used as healthy controls for a total of 14 samples, people with values of 2 and 3 (respectively, mild cognitive impairment (one impaired domain) and NO other cause of CI; and mild cognitive impairment (One impaired domain) AND another cause of CI) were used as MCI for a total of 11 samples and people with values of 4 and 5 (respectively, Alzheimer’s disease and NO other cause of CI (NINCDS PROB AD); and Alzheimer’s disease AND another cause of CI (NINCDS POSS AD)) were used as AD for a total of 22 samples. A single individual with a value of 6 (other dementia) was excluded.

For the *Macnair et al.*[37] dataset, the cells annotated as Microglia in the original dataset were selected for further processing. Two rounds of processing were performed by running NormalizeData, FindVariableFeatures, ScaleData and RunPCA with default parameters, followed by RunHarmony, RunUMAP and FindNeighbors with the top 20 principal components. The clustering resolution was 0.2 in both cases. UMAP clusters that were visually separate from most microglia were inspected and removed based on marker expression, as identified by the FindMarkers Seurat function. Clusters were excluded if they expressed CR1 (perivascular macrophages), GFAP (astrocytes), OLIG1 (oligodendrocytes) or TOP2A (cycling cells). Clusters that were outliers through having a high percentage of mitochondrial RNAs or low UMI counts were also excluded. Following the second round of processing, six Microglia clusters were identified. Clusters 2 and 3 were merged due to their similar inflammatory functions and their small size, which limited downstream power of analysis. 32,090 nuclei were used for further analysis (per microglia cluster, 44 individuals, 90 samples and 15,966 nuclei in Homeostatic, 7 individuals, 11 samples and 2,645 nuclei in Inflammatory-IRM, 5 individuals, 9 samples and 2,011 nuclei in DAM, 11 individuals, 25 samples and 5,467 nuclei in Lipid, and 7 individuals, 17 samples and 6,001 nuclei in MHCII-recovery; per lesion type, 12 individuals, 13 samples and 3,045 nuclei in control white matter, 2 individuals, 16 samples and 3,338 nuclei in normal appearing white matter, 14 individuals, 21 samples and 5,900 nuclei in active lesions, 8 individuals, 17 samples and 5,965 nuclei in chronic active lesions, 4 individuals, 13 samples and 3,091 nuclei in chronic inactive lesions, 5 individuals, 11 samples and 3,106 nuclei in remyelinating lesions, 13 individuals, 14 samples and 1,909 nuclei in control gray matter, 8 individuals, 15 samples and 1,671 nuclei in normal appearing gray matter and 12 individuals, 32 samples and 4,065 nuclei in gray matter lesions). The marker genes used to annotate the newly clustered microglia subsets are included in **Supplementary Table 4**

For the *Mathys et al.*[38] dataset, the 1,906 nuclei annotated as microglia (Mic) in the original dataset (676 for Healthy, 349 for MCI and 881 for AD) were taken for downstream analysis without further processing. The marker genes used to annotate the newly clustered microglia subsets are included in **Supplementary Table 4.**

Neuroinflammatory cytokine signature calculations

The genes identified in the mouse model were mapped to human genes using gene ortholog information as provided by the NCBI Gene Database[39], downloaded on 18 October 2023 from ftp://ftp.ncbi.nlm.nih.gov/gene/DATA/gene_orthologs.gz. Genes for which no human ortholog was found were assigned manually to human orthologs or excluded.

To calculate a summary expression score for either the CSF or the parenchymal lesion datasets, samples were pseudobulked at the individual id (for CSF and *Mathys et al.*[38]) or individual id and lesion type/cluster (for *Macnair et al.*[37]) for downstream analysis. The counts of all cells were summed and log-normalized to the number of UMIs in the pseudosample with most UMIs to minimize the impact of the pseudocount. This was done using the NormalizeData function with the parameter "scale.factor" set as the most UMIs as identified using the base R max function per dataset. To compute the cytokine-induced gene signature, the mean of the log-normalized values of the genes of the signature was calculated per pseudosample. Pseudosamples with less than ten cells were discarded from the analysis. No differences were found between male and female samples in the *Macnair et al.*[37] dataset in any of the analysed subsets (p-value > 0.98).

To control for technical variations potentially affecting gene expression levels across samples (**Extended Data Fig. 10**), one million random gene sets with the same number of genes of each of the four cytokine induced gene lists were averaged as explained above (resulting in four random

distributions, one per cytokine). Then, the mean of the resulting random distribution was subtracted from its corresponding cytokine-induced gene signature value.

Plotting of cytokine-induced gene signatures

To plot the cytokine-induced gene signatures in the human data, samples were normalized by subtracting the median value of their respective control dataset: IIH for internal and *Schafflick et al.*[35] CSF RIS/MS samples, healthy controls for *Piehl et al.*[36] samples, homeostatic microglia for microglia clusters, control white matter for white matter lesions and control gray matter for gray matter lesions.

SUPPLEMENTARY METHOD REFERENCES

1. LaFleur, M.W., Nguyen, T.H., Coxe, M.A., Yates, K.B., Trombley, J.D., Weiss, S.A., Brown, F.D., Gillis, J.E., Coxe, D.J., Doench, J.G., et al. (2019). A CRISPR-Cas9 delivery system for in vivo screening of genes in the immune system. Nat Commun 10, 1668. 10.1038/s41467-019-09656-2.
2. Erwig, M.S., Hesse, D., Jung, R.B., Uecker, M., Kusch, K., Tenzer, S., Jahn, O., and Werner, H.B. (2019). Myelin: Methods for Purification and Proteome Analysis. Oligodendrocytes. 10.1007/978-1-4939-9072-6_3.
3. Gutscher, M., Pauleau, A.-L., Marty, L., Brach, T., Wabnitz, G.H., Samstag, Y., Meyer, A.J., Dick, T.P., Gutscher, M., Pauleau, A.-L., et al. (2008). Real-time imaging of the intracellular glutathione redox potential. Nature Methods 5. 10.1038/nmeth.1212.
4. Chin, M.Y., Patwardhan, A.R., Ang, K.-H., Wang, A.L., Alquezar, C., Welch, M., Nguyen, P.T., Grabe, M., Molofsky, A.V., Arkin, M.R., and Kao, A.W. (2021). Genetically Encoded, pH-Sensitive mTFP1 Biosensor for Probing Lysosomal pH. ACS Sensors 6. 10.1021/acssensors.0c02318.
5. Kendirli, A., de la Rosa, C., Lammle, K.F., Eglseer, K., Bauer, I.J., Kavaka, V., Winklmeier, S., Zhuo, L., Wichmann, C., Gerdes, L.A., et al. (2023). A genome-wide in vivo CRISPR screen identifies essential regulators of T cell migration to the CNS in a multiple sclerosis model. Nat Neurosci 26, 1713-1725. 10.1038/s41593-023-01432-2.
6. Galaxy, C. (2024). The Galaxy platform for accessible, reproducible, and collaborative data analyses: 2024 update. Nucleic Acids Res. 10.1093/nar/gkae410.
7. Girardot, C., Scholtalbers, J., Sauer, S., Su, S.Y., and Furlong, E.E. (2016). Je, a versatile suite to handle multiplexed NGS libraries with unique molecular identifiers. BMC Bioinformatics 17, 419. 10.1186/s12859-016-1284-2.
8. Martin, M. (2011). Cutadapt removes adapter sequences from high-throughput sequencing reads. 2011 17, 3. 10.14806/ej.17.1.200.
9. Bolger, A.M., Lohse, M., and Usadel, B. (2014). Trimmomatic: a flexible trimmer for Illumina sequence data. Bioinformatics 30, 2114-2120. 10.1093/bioinformatics/btu170.

10. Li, W., Xu, H., Xiao, T., Cong, L., Love, M.I., Zhang, F., Irizarry, R.A., Liu, J.S., Brown, M., and Liu, X.S. (2014). MAGeCK enables robust identification of essential genes from genome-scale CRISPR/Cas9 knockout screens. Genome Biol 15, 554. 10.1186/s13059-014-0554-4.
11. Team, R.C. (2021). R: A Language and Environment for Statistical Computing. https://www.R-project.org/.
12. Dobin, A., Davis, C.A., Schlesinger, F., Drenkow, J., Zaleski, C., Jha, S., Batut, P., Chaisson, M., and Gingeras, T.R. (2013). STAR: ultrafast universal RNA-seq aligner. Bioinformatics 29, 15-21. 10.1093/bioinformatics/bts635.
13. Anders, S., Pyl, P.T., and Huber, W. (2015). HTSeq--a Python framework to work with high-throughput sequencing data. Bioinformatics 31, 166-169. 10.1093/bioinformatics/btu638.
14. Love, M.I., Huber, W., and Anders, S. (2014). Moderated estimation of fold change and dispersion for RNA-seq data with DESeq2. Genome Biol 15, 550. 10.1186/s13059-014-0550-8.
15. Butler, A., Hoffman, P., Smibert, P., Papalexi, E., and Satija, R. (2018). Integrating single-cell transcriptomic data across different conditions, technologies, and species. Nat Biotechnol 36, 411-420. 10.1038/nbt.4096.
16. Hao, Y., Hao, S., Andersen-Nissen, E., Mauck, W.M., 3rd, Zheng, S., Butler, A., Lee, M.J., Wilk, A.J., Darby, C., Zager, M., et al. (2021). Integrated analysis of multimodal single-cell data. Cell 184, 3573-3587 e3529. 10.1016/j.cell.2021.04.048.
17. Hao, Y., Stuart, T., Kowalski, M.H., Choudhary, S., Hoffman, P., Hartman, A., Srivastava, A., Molla, G., Madad, S., Fernandez-Granda, C., and Satija, R. (2024). Dictionary learning for integrative, multimodal and scalable single-cell analysis. Nat Biotechnol 42, 293-304. 10.1038/s41587-023-01767-y.
18. Satija, R., Farrell, J.A., Gennert, D., Schier, A.F., and Regev, A. (2015). Spatial reconstruction of single-cell gene expression data. Nat Biotechnol 33, 495-502. 10.1038/nbt.3192.
19. Stuart, T., Butler, A., Hoffman, P., Hafemeister, C., Papalexi, E., Mauck, W.M., 3rd, Hao, Y., Stoeckius, M., Smibert, P., and Satija, R. (2019). Comprehensive Integration of Single-Cell Data. Cell 177, 1888-1902 e1821. 10.1016/j.cell.2019.05.031.
20. Young, M.D., and Behjati, S. (2020). SoupX removes ambient RNA contamination from droplet-based single-cell RNA sequencing data. Gigascience 9. 10.1093/gigascience/giaa151.
21. Korsunsky, I.F., J.; Slowikowski, K.; Zhang, F.; Wei, K.; Baglaenko, Y.; Brenner, M.; Loh, P.; Raychaudhuri, S. (2019). Fast, sensitive, and accurate integration of single cell data with Harmony. bioRxiv. https://doi.org/10.1101/461954.
22. Zappia, L., and Oshlack, A. (2018). Clustering trees: a visualization for evaluating clusterings at multiple resolutions. Gigascience 7. 10.1093/gigascience/giy083.
23. Cao, J., Spielmann, M., Qiu, X., Huang, X., Ibrahim, D.M., Hill, A.J., Zhang, F., Mundlos, S., Christiansen, L., Steemers, F.J., et al. (2019). The single-cell transcriptional landscape of mammalian organogenesis. Nature 566, 496-502. 10.1038/s41586-019-0969-x.
24. Qiu, X., Mao, Q., Tang, Y., Wang, L., Chawla, R., Pliner, H.A., and Trapnell, C. (2017). Reversed graph embedding resolves complex single-cell trajectories. Nat Methods 14, 979-982. 10.1038/nmeth.4402.
25. Trapnell, C., Cacchiarelli, D., Grimsby, J., Pokharel, P., Li, S., Morse, M., Lennon, N.J., Livak, K.J., Mikkelsen, T.S., and Rinn, J.L. (2014). The dynamics and regulators of cell

fate decisions are revealed by pseudotemporal ordering of single cells. Nat Biotechnol 32, 381-386. 10.1038/nbt.2859.
26. Venables, W.N.R., B.D. (2002). Modern Applied Statistics with S, Fourth Edition (Springer).
27. Subramanian, A., Tamayo, P., Mootha, V.K., Mukherjee, S., Ebert, B.L., Gillette, M.A., Paulovich, A., Pomeroy, S.L., Golub, T.R., Lander, E.S., and Mesirov, J.P. (2005). Gene set enrichment analysis: a knowledge-based approach for interpreting genome-wide expression profiles. Proc Natl Acad Sci U S A 102, 15545-15550. 10.1073/pnas.0506580102.
28. Mootha, V.K., Lindgren, C.M., Eriksson, K.F., Subramanian, A., Sihag, S., Lehar, J., Puigserver, P., Carlsson, E., Ridderstrale, M., Laurila, E., et al. (2003). PGC-1alpha-responsive genes involved in oxidative phosphorylation are coordinately downregulated in human diabetes. Nat Genet 34, 267-273. 10.1038/ng1180.
29. Kolberg, L., Raudvere, U., Kuzmin, I., Adler, P., Vilo, J., and Peterson, H. (2023). g:Profiler-interoperable web service for functional enrichment analysis and gene identifier mapping (2023 update). Nucleic Acids Res 51, W207-W212. 10.1093/nar/gkad347.
30. Cui, A., Huang, T., Li, S., Ma, A., Perez, J.L., Sander, C., Keskin, D.B., Wu, C.J., Fraenkel, E., and Hacohen, N. (2024). Dictionary of immune responses to cytokines at single-cell resolution. Nature 625, 377-384. 10.1038/s41586-023-06816-9.
31. Thompson, A.J., Baranzini, S.E., Geurts, J., Hemmer, B., and Ciccarelli, O. (2018). Multiple sclerosis. The Lancet 391. 10.1016/S0140-6736(18)30481-1.
32. Lebrun-Frénay, C., Okuda, D.T., Siva, A., Landes-Chateau, C., Azevedo, C.J., Mondot, L., Carra-Dallière, C., Zephir, H., Louapre, C., Durand-Dubief, F., et al. (2023). The radiologically isolated syndrome: revised diagnostic criteria. Brain 146. 10.1093/brain/awad073.
33. Lublin, F.D., Reingold, S.C., Cohen, J.A., Cutter, G.R., Sørensen, P.S., Thompson, A.J., Wolinsky, J.S., Balcer, L.J., Banwell, B., Barkhof, F., et al. (2014). Defining the clinical course of multiple sclerosis. Neurology 83. 10.1212/WNL.0000000000000560.
34. Okuda, D.T., Mowry, E.M., Beheshtian, A., Waubant, E., Baranzini, S.E., Goodin, D.S., Hauser, S.L., Pelletier, D., Okuda, D.T., Mowry, E.M., et al. (2009). Incidental MRI anomalies suggestive of multiple sclerosis. Neurology 72. 10.1212/01.wnl.0000335764.14513.1a.
35. Schafflick, D., Xu, C.A., Hartlehnert, M., Cole, M., Schulte-Mecklenbeck, A., Lautwein, T., Wolbert, J., Heming, M., Meuth, S.G., Kuhlmann, T., et al. (2020). Integrated single cell analysis of blood and cerebrospinal fluid leukocytes in multiple sclerosis. Nat Commun 11, 247. 10.1038/s41467-019-14118-w.
36. Piehl, N., van Olst, L., Ramakrishnan, A., Teregulova, V., Simonton, B., Zhang, Z., Tapp, E., Channappa, D., Oh, H., Losada, P.M., et al. (2022). Cerebrospinal fluid immune dysregulation during healthy brain aging and cognitive impairment. Cell 185, 5028-5039 e5013. 10.1016/j.cell.2022.11.019.
37. Macnair, W., Calini, D., Agirre, E., Bryois, J., Jäkel, S., Smith, R.S., Kukanja, P., Stokar-Regenscheit, N., Ott, V., Foo, L.C., et al. (2025). snRNA-seq stratifies multiple sclerosis patients into distinct white matter glial responses. Neuron 113. 10.1016/j.neuron.2024.11.016.
38. Mathys, H., Davila-Velderrain, J., Peng, Z., Gao, F., Mohammadi, S., Young, J.Z., Menon, M., He, L., Abdurrob, F., Jiang, X., et al. (2019). Single-cell transcriptomic analysis of Alzheimer's disease. Nature 570, 332-337. 10.1038/s41586-019-1195-2.

39. Sayers, E.W., Bolton, E.E., Brister, J.R., Canese, K., Chan, J., Comeau, D.C., Connor, R., Funk, K., Kelly, C., Kim, S., et al. (2022). Database resources of the national center for biotechnology information. Nucleic Acids Res 50, D20-D26. 10.1093/nar/gkab1112.
