# Supplementary material for: In vivo CRISPR screen reveals regulation of macrophage states in neuroinflammation
Source: Nat Neurosci. 2025 Dec 4;29(2):493–509. doi: 10.1038/s41593-025-02151-6 (PMC12880918; doi:10.1038/s41593-025-02151-6)
Supplement: Supplementary file 2 — Reporting Summary [file 41593_2025_2151_MOESM2_ESM.pdf]

nature portfolio

Corresponding author(s): Martin Kerschensteiner, Arek Kendirli

Last updated by author(s): Oct 15, 2025

# Reporting Summary

Nature Portfolio wishes to improve the reproducibility of the work that we publish. This form provides structure for consistency and transparency in reporting. For further information on Nature Portfolio policies, see our Editorial Policies and the Editorial Policy Checklist.

## Statistics

For all statistical analyses, confirm that the following items are present in the figure legend, table legend, main text, or Methods section.

| n/a | Confirmed | |
|---|---|---|
| ☐ | ☒ | The exact sample size (*n*) for each experimental group/condition, given as a discrete number and unit of measurement |
| ☐ | ☒ | A statement on whether measurements were taken from distinct samples or whether the same sample was measured repeatedly |
| ☐ | ☒ | The statistical test(s) used AND whether they are one- or two-sided<br>*Only common tests should be described solely by name; describe more complex techniques in the Methods section.* |
| ☐ | ☒ | A description of all covariates tested |
| ☐ | ☒ | A description of any assumptions or corrections, such as tests of normality and adjustment for multiple comparisons |
| ☐ | ☒ | A full description of the statistical parameters including central tendency (e.g. means) or other basic estimates (e.g. regression coefficient) AND variation (e.g. standard deviation) or associated estimates of uncertainty (e.g. confidence intervals) |
| ☐ | ☒ | For null hypothesis testing, the test statistic (e.g. *F*, *t*, *r*) with confidence intervals, effect sizes, degrees of freedom and *P* value noted<br>*Give P values as exact values whenever suitable.* |
| ☒ | ☐ | For Bayesian analysis, information on the choice of priors and Markov chain Monte Carlo settings |
| ☒ | ☐ | For hierarchical and complex designs, identification of the appropriate level for tests and full reporting of outcomes |
| ☐ | ☒ | Estimates of effect sizes (e.g. Cohen's *d*, Pearson's *r*), indicating how they were calculated |

*Our web collection on statistics for biologists contains articles on many of the points above.*

## Software and code

Policy information about availability of computer code

| | |
|---|---|
| Data collection | Details in methods. Instruments: Leica SP8X STED 3D DLS WLL, ClarioSTAR plate reader, Leica SP8 confocal, Olympus RS-FV4000 confocal, Leica SP8 WLL DIVE FALCON confocal, Illumina NextSeq 1000, Illumina NextSeq 2000, Agilent Bioanalyzer, Qubit 4, FACS Aria III (BD), FACS Fusion (BD), Fortessa (BD), Cytoflex S (Beckman Coulter) |
| Data analysis | Details in methods. Software: GraphPad Prism (v7 and v9), R (v4.0.0+), Je-Demultiplex, cutadapt, trimmomatic, MAGeCK, Galaxy, RNA STAR (v2.7.2b), HTSeq count (v1.0.0), DeSeq2 (v2.11.40.7+), Fiji/ImageJ, Seurat (v4.0.0+), Adobe Illustrator 2025, Imaris software (v9.7.2) |

For manuscripts utilizing custom algorithms or software that are central to the research but not yet described in published literature, software must be made available to editors and reviewers. We strongly encourage code deposition in a community repository (e.g. GitHub). See the Nature Portfolio guidelines for submitting code & software for further information.

## Data

Policy information about availability of data

All manuscripts must include a data availability statement. This statement should provide the following information, where applicable:

- Accession codes, unique identifiers, or web links for publicly available datasets
- A description of any restrictions on data availability
- For clinical datasets or third party data, please ensure that the statement adheres to our policy

All data generated or analysed during this study is included in the published article as supplementary materials, in Zenodo 10.5281/zenodo.15808138 or is available from the corresponding authors upon reasonable request.

## Research involving human participants, their data, or biological material

Policy information about studies with human participants or human data. See also policy information about sex, gender (identity/presentation), and sexual orientation and race, ethnicity and racism.

| | |
|---|---|
| Reporting on sex and gender | n/a |
| Reporting on race, ethnicity, or other socially relevant groupings | n/a |
| Population characteristics | CSF sampling was performed either to confirm the diagnosis of Relapsing-Remitting MS according to the 2017 revision of the McDonald criteria(31) (RRMS), for diagnostic evaluation of suspected Radiologically Isolated Syndrome (RIS) according the proposed RIS-diagnostic criteria(32), or for therapeutic CSF removal in people with Idiopathic Intracranial Hypertension (IIH). Persons with RRMS had not received prior disease modifying therapy. They had experienced a clinical relapse in a median of 59 days (interquartile range 21,5 – 170,25) before CSF sampling. 5 patients had received steroid-based relapse therapy up until a median of 92 days (minimum 24, maximum 1621) in advance. RRMS in all individuals was considered "active" according to Lublin et al., 2014(33), as either a clinical relapse, or a new or unequivocally enlarging or Gadolinium-enhancing lesion on brain or spinal cord magnetic resonance imaging was present within 365 days prior to sampling. People with white matter lesions were referred for CSF analysis to evaluate RIS. |
| Recruitment | Samples were collected at the Institute of Clinical Neuroimmunology at the LMU Klinikum Munich, Germany.<br>Recruitment of individuals took place from August 2020 to January 2021 |
| Ethics oversight | CSF sampling was performed to confirm the diagnosis of relapsing-remitting MS according to the revised McDonald criteria for all individuals included in the study.<br>Collection of CSF was approved by the local ethics committees of the LMU, Munich (ethical vote: 163-16).<br>Written informed consent was obtained from all subjects according to the Declaration of Helsinki |

Note that full information on the approval of the study protocol must also be provided in the manuscript.

# Field-specific reporting

Please select the one below that is the best fit for your research. If you are not sure, read the appropriate sections before making your selection.

☒ Life sciences ☐ Behavioural & social sciences ☐ Ecological, evolutionary & environmental sciences

For a reference copy of the document with all sections, see nature.com/documents/nr-reporting-summary-flat.pdf

# Life sciences study design

All studies must disclose on these points even when the disclosure is negative.

| | |
|---|---|
| Sample size | Indicated in figure legend |
| Data exclusions | scRNAseq exclusion criteria for low QC are detailed in the methods. No other data was excluded |
| Replication | All experiments include more than one replicate |
| Randomization | Animals were assigned randomly to experimental groups where applicable, or when not possible, the conditions were appropriately blocked such that there were animals of the same condition in all cages |
| Blinding | Due to the experimental design and animal laws, no blinding was possible during FACS experiments and Hoxb8FL characterization histology<br>The analysis of all histological data was done blinded |

# Reporting for specific materials, systems and methods

We require information from authors about some types of materials, experimental systems and methods used in many studies. Here, indicate whether each material, system or method listed is relevant to your study. If you are not sure if a list item applies to your research, read the appropriate section before selecting a response.

## Materials & experimental systems

| n/a | Involved in the study |
|---|---|
| ☐ | ☒ Antibodies |
| ☐ | ☒ Eukaryotic cell lines |
| ☒ | ☐ Palaeontology and archaeology |
| ☐ | ☒ Animals and other organisms |
| ☒ | ☐ Clinical data |
| ☒ | ☐ Dual use research of concern |
| ☒ | ☐ Plants |

## Methods

| n/a | Involved in the study |
|---|---|
| ☒ | ☐ ChIP-seq |
| ☐ | ☒ Flow cytometry |
| ☒ | ☐ MRI-based neuroimaging |

## Antibodies

Antibodies used

TruStain Fc Block CD16/32 (93) Biolegend 101320 RRID: AB_1574975 1:100
LIVE/DEAD™ Fixable Near-IR Thermo Fisher L10119 1:1000
Arg1-PE (A1exF5) Thermo Fisher 12-3697-80 RRID: AB_2734839 1:100
Arg1-APC (A1exF5) Thermo Fisher 17-3697-82 RRID: AB_2734835 1:100
CD11b-PerCP (M1/70) Biolegend 101230 RRID: AB_2129374 1:100
F4/80-PE (BM8) Biolegend 123109 RRID: AB_893498 1:100
CD45-BV786 (38-F11) Biolegend 103149 RRID: AB_2564590 1:100
iNOS-e450 (CXNFT) Thermo Fisher 48-5920-80 RRID: AB_2802293 1:100
MHCII(I-A/I-E)-APC (M5/114.15.2) Biolegend 107613 RRID: AB_313328 1:100
CD45-APC-Cy7 (38-F11) Biolegend 103115 RRID: AB_312980 1:100
NK1.1-APC-Cy7 (PK136) Biolegend 108723 RRID: AB_830870 1:100
Cd11b-AF647 (M1/70) Biolegend 101218 RRID:AB_389327 1:100
CD107a (1D4B) Biolegend 121602 RRID:AB_572021 1:200
GPNMB-eF660 (CTSREVL) Thermo Fisher 50-5708-82 RRID:AB_2574239 1:100
MBP Thermo Fisher PA1-10008 RRID:AB_1077024 1:200
Lyve1 (ALY7) Thermo Fisher 14-0443-82 RRID:AB_1633414 1:100
Laminin Sigma-Aldrich L9393 RRID:AB_477163 1:200
Fibronectin Sigma-Aldrich AB2033 RRID:AB_2105702 1:200
Perilipin-2/ADFP Novus Biologicals NB110-40877 1:200
CD206 (MR5D3) BioRad MCA2235 RRID:AB_324622 1:100
IBA1 Synaptic Systems HS-234 017 1:200
Goat anti-rabbit Alexa Fluor™ 647 Thermo Fisher Scientific, # A-21245 1:1000
Donkey anti-rat Alexa Fluor™ Plus 647 Thermo Fisher Scientific, # A48272 1:1000
Donkey anti-rat Alexa Fluor™ Plus 405 Thermo Fisher Scientific, # A48268 1:1000
Goat anti-chicken Alexa Fluor™ 647 Thermo Fisher Scientific, # A-21449 1:1000

Validation

Antibodies were chosen based on the validation statements and application on the manufacturer's website.

## Eukaryotic cell lines

Policy information about cell lines and Sex and Gender in Research

Cell line source(s)

HEK293 T cells (ATCC)

Authentication

Cell line was not authenticated after purchase

Mycoplasma contamination

Cell line tested negative for mycoplasma contamination

Commonly misidentified lines
(See ICLAC register)

No commonly misidentified cell lines were used.

## Animals and other research organisms

Policy information about studies involving animals; ARRIVE guidelines recommended for reporting animal research, and Sex and Gender in Research

Laboratory animals

Mice of strains C57BL6/J, C57BL6/J x BiozziABH and 2D2, between eight and thirty weeks old, were used in this study

| | |
|---|---|
| Wild animals | *Provide details on animals observed in or captured in the field; report species and age where possible. Describe how animals were caught and transported and what happened to captive animals after the study (if killed, explain why and describe method; if released, say where and when) OR state that the study did not involve wild animals.* |
| Reporting on sex | Animals of both sexes were used |
| Field-collected samples | *For laboratory work with field-collected samples, describe all relevant parameters such as housing, maintenance, temperature, photoperiod and end-of-experiment protocol OR state that the study did not involve samples collected from the field.* |
| Ethics oversight | All animal experiments were approved by the Regierung Oberbayern |

Note that full information on the approval of the study protocol must also be provided in the manuscript.

# Plants

| | |
|---|---|
| Seed stocks | *Report on the source of all seed stocks or other plant material used. If applicable, state the seed stock centre and catalogue number. If plant specimens were collected from the field, describe the collection location, date and sampling procedures.* |
| Novel plant genotypes | *Describe the methods by which all novel plant genotypes were produced. This includes those generated by transgenic approaches, gene editing, chemical/radiation-based mutagenesis and hybridization. For transgenic lines, describe the transformation method, the number of independent lines analyzed and the generation upon which experiments were performed. For gene-edited lines, describe the editor used, the endogenous sequence targeted for editing, the targeting guide RNA sequence (if applicable) and how the editor was applied.* |
| Authentication | *Describe any authentication procedures for each seed stock used or novel genotype generated. Describe any experiments used to assess the effect of a mutation and, where applicable, how potential secondary effects (e.g. second site T-DNA insertions, mosiacism, off-target gene editing) were examined.* |

# Flow Cytometry

## Plots

Confirm that:

☒ The axis labels state the marker and fluorochrome used (e.g. CD4-FITC).

☒ The axis scales are clearly visible. Include numbers along axes only for bottom left plot of group (a 'group' is an analysis of identical markers).

☒ All plots are contour plots with outliers or pseudocolor plots.

☒ A numerical value for number of cells or percentage (with statistics) is provided.

## Methodology

| | |
|---|---|
| Sample preparation | Detailed in Methods section: Flow Cytometry |
| Instrument | FACS Aria III (BD), FACS Fusion (BD), Fortessa (BD) or Cytoflex S (Beckman Coulter). |
| Software | FlowJo_v10.10.10 |
| Cell population abundance | Indicated in quantifications |
| Gating strategy | Extended Data Figures 1 and 2. Briefly, Lymphocytes > Singlets > Live cells were gated before cell type specific markers were used |

☒ Tick this box to confirm that a figure exemplifying the gating strategy is provided in the Supplementary Information.
